# Indium-Catalyzed Annulation of *o*-Acylanilines with Alkoxyheteroarenes: Synthesis of Heteroaryl[*b*]quinolines and Subsequent Transformation to Cryptolepine Derivatives

**DOI:** 10.3390/molecules23040838

**Published:** 2018-04-05

**Authors:** Kyohei Yonekura, Mika Shinoda, Yuko Yonekura, Teruhisa Tsuchimoto

**Affiliations:** Department of Applied Chemistry, School of Science and Technology, Meiji University, 1-1-1 Higashimita, Tama-ku, Kawasaki 214-8571, Japan; kyohei@meiji.ac.jp (K.Y.); mika.shinoda.21@gmail.com (M.S.); y.tonyfanfan@gmail.com (Y.Y.)

**Keywords:** anti-cancer activity, anti-malarial activity, heteroarenes, indium, Lewis acids, pyridine, one-pot, quindolines, tandem reaction

## Abstract

We disclose herein the first synthetic method that is capable of offering heteroaryl[*b*]quinolines (HA[*b*]Qs) with structural diversity, which include tricyclic and tetracyclic structures with (benzo)thienyl, (benzo)furanyl, and indolyl rings. The target HA[*b*]Q is addressed by the annulation of *o*-acylanilines and MeO–heteroarenes with the aid of an indium Lewis acid that effectively works to make two different types of the N–C and C–C bonds in one batch. A series of indolo[3,2-*b*]quinolines prepared here can be subsequently transformed to structurally unprecedented cryptolepine derivatives. Mechanistic studies showed that the N–C bond formation is followed by the C–C bond formation. The indium-catalyzed annulation reaction thus starts with the nucleophilic attack of the NH_2_ group of *o*-acylanilines to the MeO-connected carbon atom of the heteroaryl ring in an S_N_Ar fashion, and thereby the N–C bond is formed. The resulting intermediate then cyclizes to make the C–C bond through the nucleophilic attack of the heteroaryl-ring-based carbon atom to the carbonyl carbon atom, providing the HA[*b*]Q after aromatizing dehydration.

## 1. Introduction

**Figure 1:**
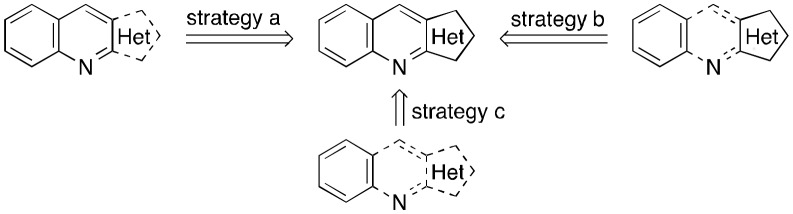
Synthetic strategies for the construction of the heteroaryl[*b*]quinoline (HA[*b*]Q) structure.

Heteroaryl[*b*]quinolines (HA[*b*]Qs), wherein electron-rich heteroaryl rings are fused to the [*b*] site of quinoline, are important frameworks found in natural products [1,2,3] and biologically active molecules [1,4,5,6] as well as functional organic materials [7,8,9]. Due to their significance, numerous synthetic approaches have been developed for the construction of such structural motifs. These approaches could be categorized simply into three strategies on the basis of the ring-constructing method (Figure 1), which are the heteroaryl ring formation (strategy a) [10,11,12,13,14,15,16,17,18,19,20,21,22], the central pyridyl ring formation (strategy b) [23,24,25,26,27,28,29,30,31,32,33,34,35,36,37,38,39], and the formation of both rings (strategy c) [4,40,41,42,43,44,45,46,47,48,49,50,51,52,53]. Although there are advantages and disadvantages to each strategy from various aspects, the strategy b seems to be the most user-friendly in terms of the accessibility of the starting substrates.

**Scheme 1:**
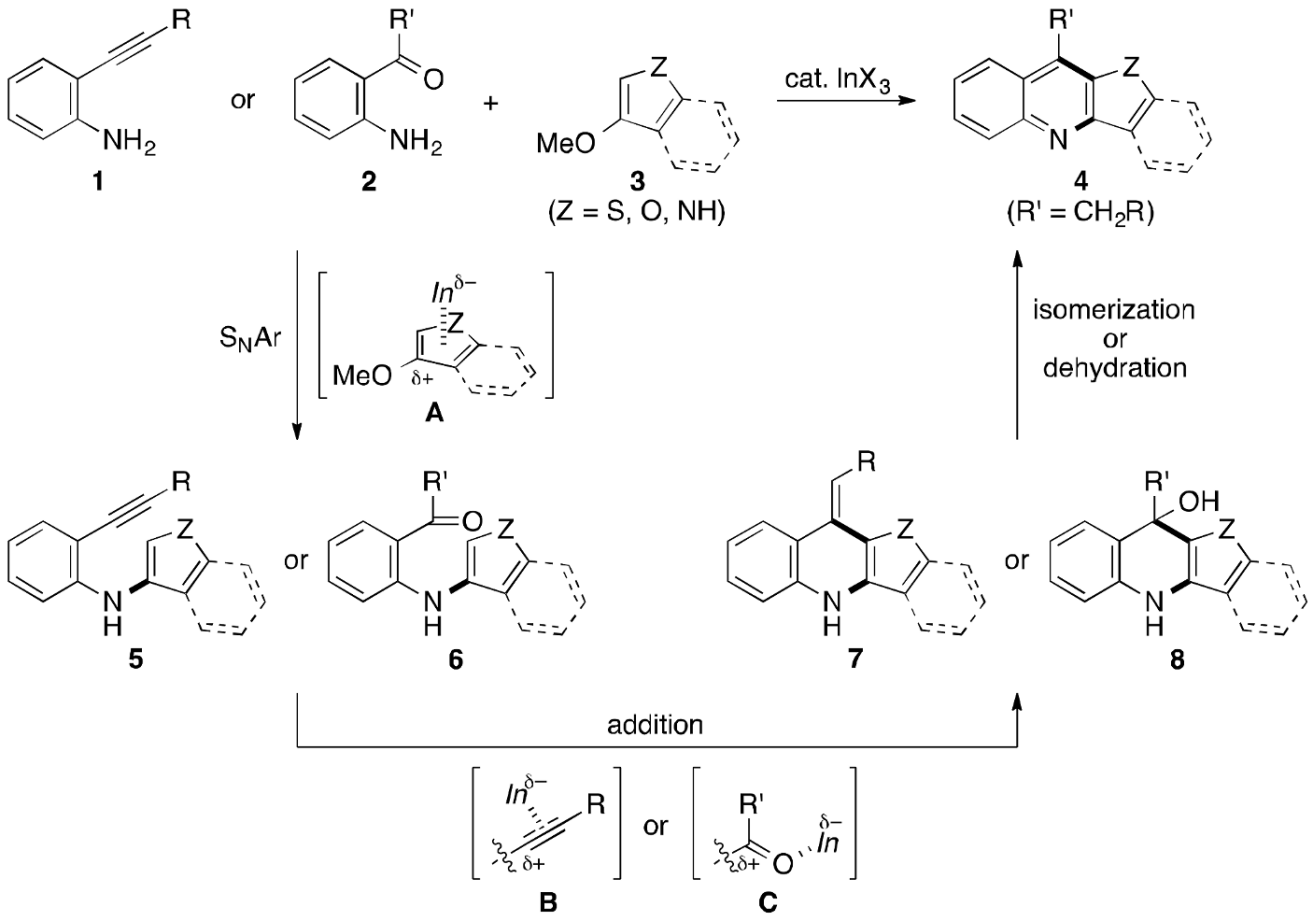
A working hypothesis for the synthesis of HA[*b*]Qs **4**. *In* = InX_3_.

On the other hand, we have recently reported a new C(heteroaryl)–N bond-forming reaction by reacting electron-rich methoxyheteroarenes with amines via a nucleophilic aromatic substitution (S_N_Ar) reaction [54]. In addition to this, we have also developed several new C(heteroaryl)–C bond-forming reactions by reacting alkynes [55,56,57] or carbonyl compounds [58,59,60] with heteroarenes. All of these reactions are effectively catalyzed by a salt of an indium(III) Lewis acid, which has been also employed for various organic transformations by other research groups [61,62,63,64,65,66,67,68,69,70,71]. We therefore envisaged that conducting the two different types of reactions in a tandem fashion would be a new methodology of the strategy b to offer the HA[*b*]Q in an easy way, thereby also leading to the further expansion of our indium-based technology. Our working hypothesis is illustrated more intelligibly in Scheme 1. We thus expected that the synthesis of HA[*b*]Qs **4** could be achieved by mixing *o*-alkynylanilines **1** or *o*-acylanilines **2** with methoxyheteroarenes **3** in the presence of a catalytic indium Lewis acid (InX_3_ = *In*). The first stage is the S_N_Ar-based N–C bond-forming reaction through the nucleophilic attack of the amino group of **1** or **2** to electrophilic complex **A** to afford **5** or **6**, respectively. Intermediate **5** or **6** successively cyclizes by forming the C–C bond in an intramolecular fashion, thus giving **7** or **8**, respectively, via the activation mode of **B** or **C**. The isomerization or dehydration as the final stage results in the formation of desired HA[*b*]Q **4**. We also expected that combining the two indium transformations, both of which are compatible with a broad range of substrates, should lead to the development of the HA[*b*]Q synthesis with good substrate generality. As stated above, a lot of studies that synthesize the HA[*b*]Q have appeared so far in literature, but these studies have been limited to preparing HA[*b*]Qs with one to three types of heteroaryl rings, to the best of our knowledge [72,73,74]. We report herein that an indium salt effectively catalyzes the N–C and C–C bond-forming sequence to afford a range of HA[*b*]Qs including tricyclic and tetracyclic [2,3-*b*] and [3,2-*b*] structures with sulfur-, oxygen-, and nitrogen-based five-membered heteroaryl rings. Among the products, indolo[3,2-*b*]quinolines, which can be easily converted to cryptolepine derivatives that have been known to exhibit anti-malarial and anti-cancer activities, are included [75].

## 2. Results and Discussion

In order to verify the working hypothesis, we first investigated the possibility of whether *o*-ethynylaniline (**1a**) works as a substrate for the synthesis of the HA[*b*]Q under indium catalysis, and selected 3-methoxybenzothiophene (**3a**) as the substrate partner (Table 1). Upon treatment of **1a** and **3a** with 5 mol % of In(NTf_2_)_3_ (Tf = SO_2_CF_3_) in PhCl at 110 °C for 24 h, we were pleased to observe that the desired annulation proceeded to give 11-methyl[1]benzothieno[3,2-*b*]quinoline (**4aa**), albeit in low yield (entry 1). While the screening of other indium salts provided no significant improvements in the yield of **4aa**, a small amount of *o*-acetylaniline (**2a**) was formed along with **4aa** when using In(ONf)_3_ (Nf = SO_2_C_4_F_9_) as a catalyst (entries 2–6). In this context, a wide variety of Lewis acids, including indium salts, have been known to act as catalysts for the hydration of a C≡C bond to create a carbonyl functionality [76,77]. A possible explanation for the formation of **2a** is thus the indium-catalyzed hydration of **1a** with H_2_O, which could have been present in a small quantity in the reaction mixture. Accordingly, we presumed that, as routes for the formation of **4aa**, there would be two possibilities: one is directly from **1a**, and the other is indirectly from **2a** formed in situ after the hydration of **1a**. In order to get an insight into which routes operate here, the following experiments were conducted. Thus, the annulation carried out under the conditions of entry 3, additionally including five molar equivalents of H_2_O, resulted in higher yields of both **4aa** and **2a** (entry 7). Moreover, the prolonged reaction time from 24 h to 36 h raised the yield of **4aa** to 61% with the complete consumption of **2a** (entry 8). These results suggest that **4aa** is likely to be formed through the generation of **2a** by the hydration of **1a**, whereas the contribution of the direct route from **1a** cannot be completely excluded.

On the basis of the above results, we turned our attention to the annulation with **2a** instead of **1a** (Table 2). As expected, under the same reaction conditions as those for entry 3 of Table 1, **4aa** was produced in significantly higher yield of 62% (entry 1). Inspired by this result, we continuously examined the effect of various indium salts other than In(ONf)_3_ for the same annulation reaction of **2a** with **3a**. Thus, In(OTf)_3_ and In(NTf_2_)_3_ with the strong electron-withdrawing ligands as In(ONf)_3_ also catalyzed the annulation, and the yield of **4aa** increased to 74% in the use of In(NTf_2_)_3_ (entries 2 and 3). Among the indium halides examined, InBr_3_ and InI_3_ were found to be highly effective, giving **4aa** in 92% yield in both the cases, in sharp contrast to the inactivity of the fluoride salt (entries 4–7). However, the corresponding hydroxide and acetate salts were totally inactive (entries 8 and 9). Due to the remarkable catalytic activity of InBr_3_, metal bromides of, for instance, Sc, Fe, Co, Pd, Cu, Ag, Zn, Pb, and Bi were tested, but proved to be less effective (entries 10–18). No **4aa** was formed in the absence of a catalyst, which is thus indispensable for the progress of the annulation (entry 19). With InBr_3_ as the promising catalyst, a continuous survey of the solvent effect indicated that PhCl would be the most suitable solvent of choice for the annulation, and that the reaction rate greatly decreases in H_2_O (entries 20–27). While the lowering of the catalyst loading to 1 mol % accompanies the decrease of the reaction rate, the good yield of **4aa** can be secured by extending the reaction time to 96 h (entry 28). Favorably, the annulation can be also carried out under an atmosphere of air instead of argon to afford **4aa** in 88% yield (entry 29).

With the proper reaction conditions in hand, we next examined the scope of the *o*-acylaniline substrate to **3a** (Table 3). Similar to *o*-acetylaniline (**2a**), its derivatives with the OH, OMe, or methylenedioxy group successfully participated in the annulation (**4aa**–**4da**). The formation of **4ba** in such high yield shows that the OH group does not interfere with the progress of the desired annulation by acting as the nucleophilic site, as the NH_2_ group does. No undesired ring fragmentation of the acetal moiety in **4da** was observed, even under the Lewis acidic conditions [78]. The bulkier isopropyl group on the carbonyl carbon atom does not affect the efficiency of the annulation, giving **4ea** in 97% yield. A CF_3_ group, the C–F bond of which is known to increase metabolic stability and membrane permeability, thus leading to improvement in bioavailability [79], can be also installed onto the C11-position of the benzothieno[3,2-*b*]quinoline structure (**4fa** and **4ga**). A commercially available hydrochloride–hydrate adduct of *o*-acylaniline **2g** can be used as a substrate without neutralizing and drying. Our protocol is applicable as well to *o*-acylanilines with a series of aryl groups with different electronic and steric natures, in which the simple phenyl group for **4ha** and **4ia**, *p*-MeOC_6_H_4_ for **4ja**, *p*-FC_6_H_4_ for **4ka**, *o*-MeC_6_H_4_ for **4la**, and *o*-fused-aroylC_6_H_4_ for **4ma** are included. The atmosphere of air was again confirmed to be available on the synthesis of **4ha**. In the reaction of aminoanthraquinone **2m** with two carbonyl moieties, only the one adjacent to the NH_2_ group worked as a reaction site to provide hexacyclic-fused ring system **4ma** in one shot. Of importance to note is that the MeO, Cl, and F groups on the aryl ring are known to behave as leaving groups in the general S_N_Ar reaction, but were found to be compatible with the reaction conditions, thus contributing to the high-yield formation of the target molecules (**4ca**, **4ga**, **4ia**, **4ja**, and **4ka**) [80].

**Figure 2:**
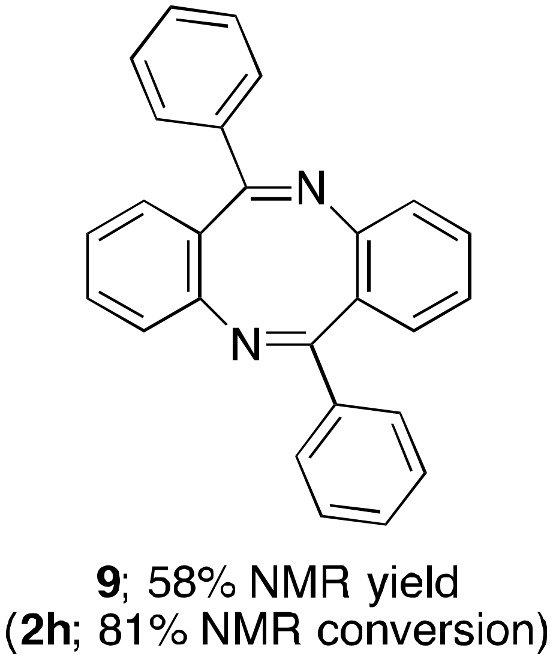
A major byproduct formed in the reaction of **2h** with **3e**.

Besides the benzothieno[3,2-*b*]quinoline, our method is applicable to preparing a range of HA[*b*]Qs by using other sulfur- and oxygen-based methoxyheteroarenes (Table 4). The replacement of **3a** with 2-methoxybenzothiophene (**3b**) enables the switch of the fused-ring orientation from the [3,2-*b*] to the [2,3-*b*], and products **4ab** and **4bb** were obtained in high yields. However, in contrast to the successful construction of thieno[2,3-*b*]quinoline **4ac**, **4hc**, and **4ad**, changing the fused-ring orientation to the [3,2-*b*] in this case resulted in low yield of **4he**. In the reaction of 3-methoxythiophene (**3e**), a self-condensation reaction, in which two molecules of **2h** react with each other to form cyclic diimine **9**, occurred as a major side reaction (Figure 2). This result is likely to be related, at least in part, to the relatively low reaction rate of the desired S_N_Ar process between **2h** and **3e**, and, in fact, 70% of **3e** loaded for the reaction remained unconsumed. In this context, we have previously confirmed that the S_N_Ar amination reaction of 3-methoxythiophene (**3e**) requires a higher loading of an indium catalyst as well as higher temperature compared to those for the reaction of 2-methoxythiophene (**3c**) [54]. In addition to the sulfur-containing HA[*b*]Qs, the tetracyclic and tricyclic oxygen-containing analogues can be addressed by our method in moderate to good yields (**4af**, **4hf**, and **4ag**). When preparing **4ag**, InI_3_ worked as a catalyst more efficiently than InBr_3_. Unfortunately, no annulation reaction of **2a** with 2-methoxy-1-phenylpyrrole (**3h**) for the synthesis of pyrrolo[2,3-*b*]quinoline **4ah** proceeded, due to some undesired side reactions, including *N*-methylation of **2a** by the MeO group of **3h** acting as a source of a methyl group.

As collected separately in Table 5, we successively present the result of constructing the framework of the indolo[3,2-*b*]quinoline, which is alternatively named quindoline, having been known to show cytotoxic activity against human cancer cell lines [81]. As in our preceding S_N_Ar amination [54], commercially unavailable 3-methoxyindole was not required, but rather commercially available 3-acetyloxyindole (**3i**) can be used here again as a substrate. Thus, mixing **2a**, **3i**, and InBr_3_ (5 mol %) in PhCl, and then heating the mixture at 110 °C for 24 h gave **4ai** in 55% yield. Other quindoline derivatives **4di**, **4fi**, and **4gi** could also be synthesized by our method. Unlike the annulation of **2g**–HCl–H_2_O with 3-methoxybenzothiophene (**3a**) (see **4ga** in Table 3), the pre-removal of HCl and H_2_O from **2g**–HCl–H_2_O as a commercial source is required here to obtain **4gi** in reasonable yield. With **2g**–HCl–H_2_O instead, the formation of **4gi** resulted in only 1% NMR yield. These results inspired us to address cryptolepine derivatives, due to their potentialities as anti-malarial and/or anti-cancer drugs.

**Figure 3:**
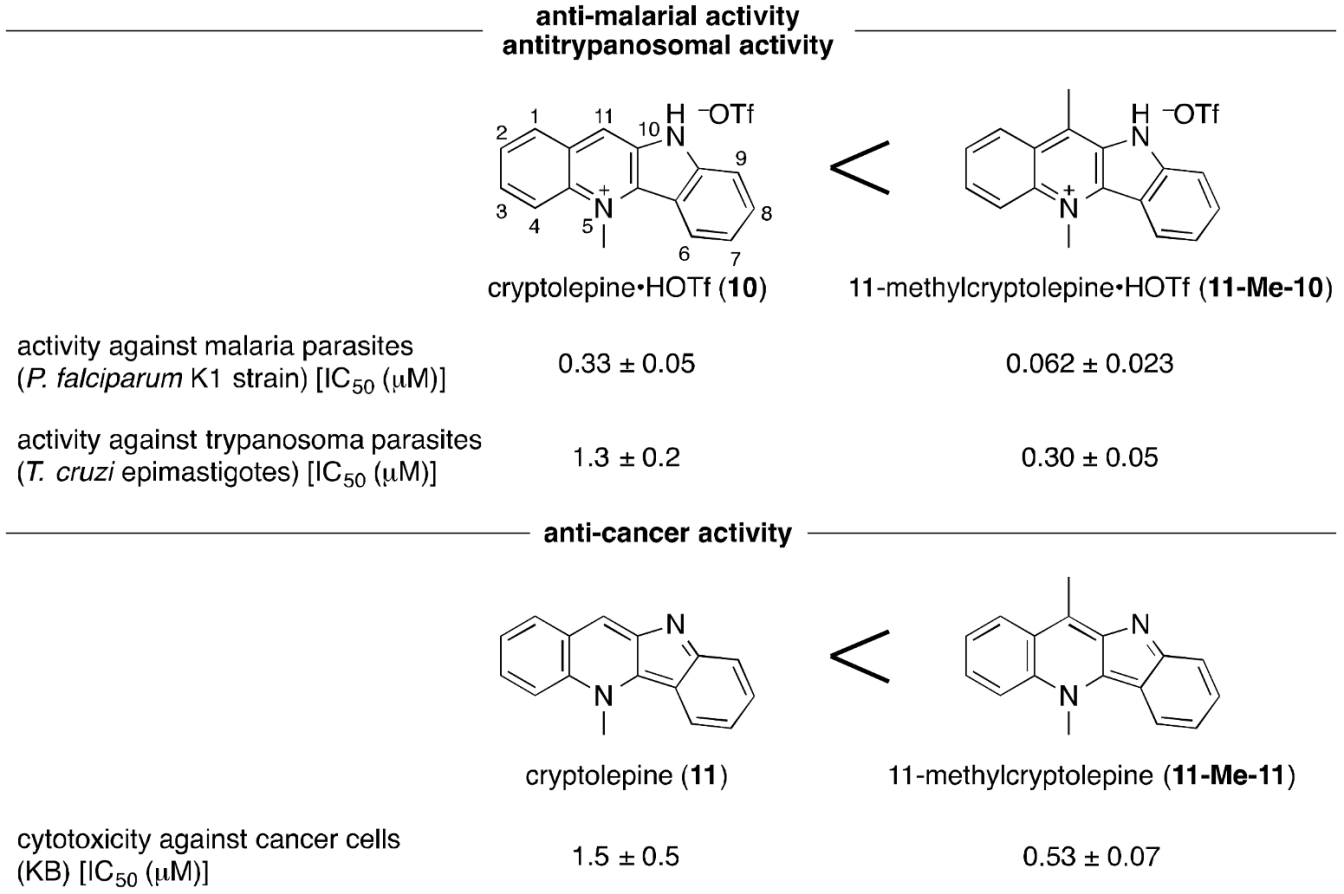
Anti-malarial, antitrypanosomal and anti-cancer activities of cryptolepine, 11-methylcryptolepine, and their HOTf adducts [82].

**Scheme 2:**
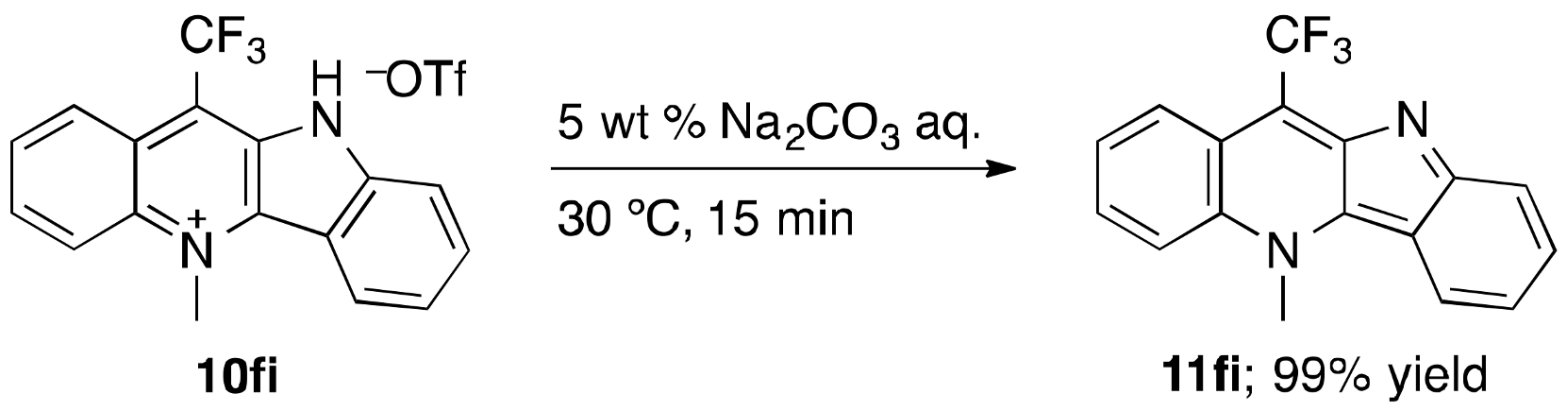
Neutralization of **10fi**.

As previously demonstrated, the HOTf adduct of the 11-methylated cryptolepine (**11-Me-10**) shows higher anti-malarial and antitrypanosomal activities than that of the original cryptolepine (**10**) (Figure 3). Since the *N*-methylation of the pyridine ring of **4ai** with methyl triflate (MeOTf) has been already reported [82], we targeted the synthesis of analogues thereof from other quindoline derivatives **4di**, **4fi**, and **4gi** (Table 6). The *N*-methylation in accordance with the modified literature procedure successfully delivered **10di**, **10fi**, and **10gi**, which are new compounds unreported in the literature [82]. Especially, **10fi**, which has the 11-CF_3_ group instead of the 11-CH_3_ group in **11-Me-10**, might be expected to be promising in view of anti-malarial and antitrypanosomal activities, due to the possible higher bioavailability. Moreover, since the acid-free cryptolepine derivatives have been the focus of examining anti-cancer activity (**11** and **11-Me-11** in Figure 3), there should be a demand for the acid-free form. Accordingly, we confirmed that the neutralization of, for instance, **10fi** with a Na_2_CO_3_ aqueous solution provides **11fi** with no TfOH in quantitative yield (Scheme 2).

**Scheme 3:**
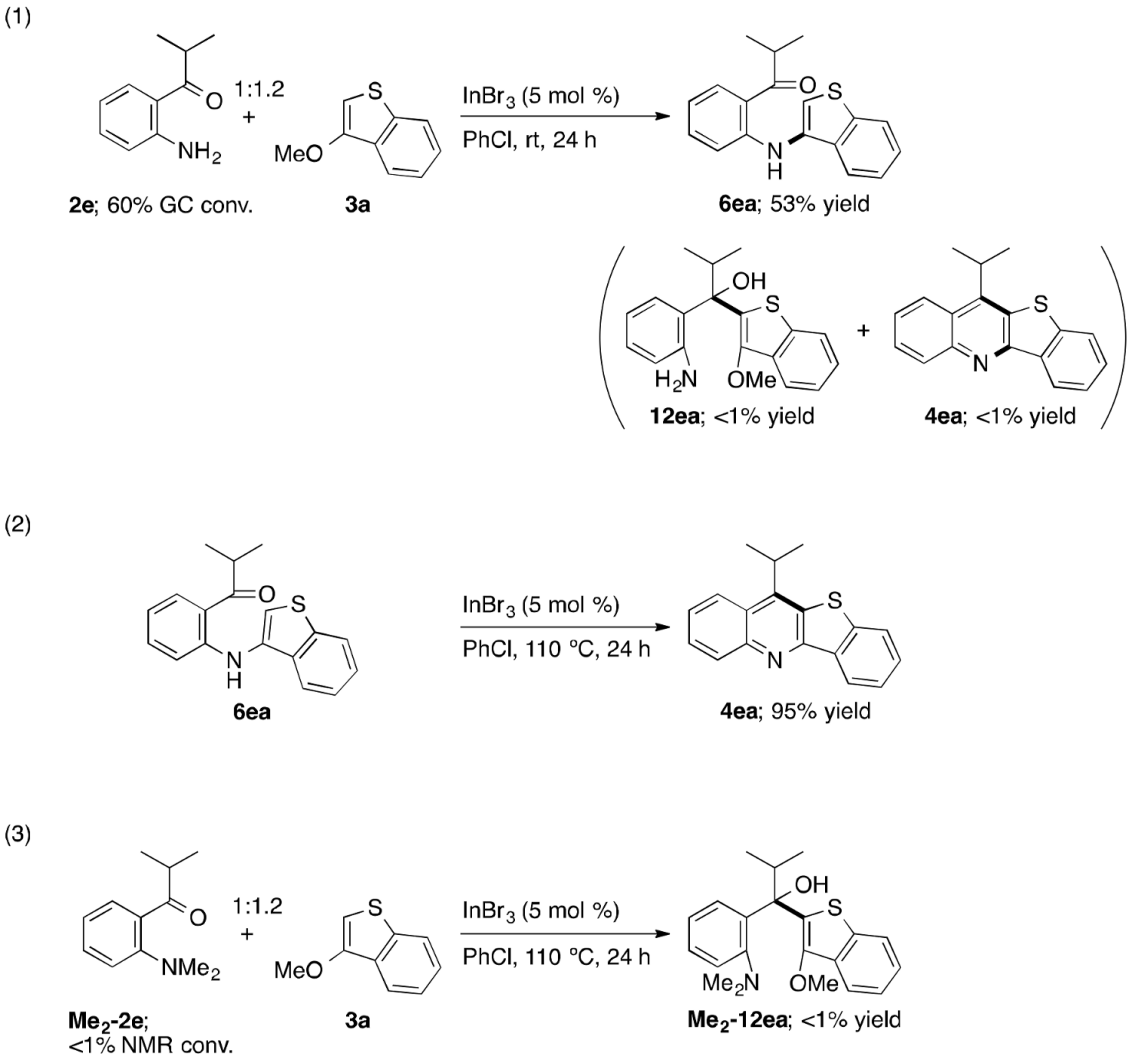
Control experiments for mechanistic studies.

In order to get insight into the reaction pathway of the present annulation reaction, some experiments were performed (Scheme 3). At first, upon treating **2e** with **3a** at room temperature rather than the standard heating temperature, only the S_N_Ar-based intermolecular N–C bond-forming reaction proceeded to furnish **6ea** in 53% yield with 60% conversion of **2e**, thus being not contaminated by **12ea** derived from the C–C bond formation as a possible alternative first stage, and by final annulation product **4ea** [Equation (1) in Scheme 3]. Subsequently, **6ea** isolated from the reaction of Equation (1) was heated under the standard reaction conditions, and thereby **4ea** was obtained highly efficiently via the intramolecular C–C bond-forming annulation [Equation (2) in Scheme 3]. On the other hand, **Me_2_-2e**, wherein the nitrogen atom is dimethylated and would thus no longer act as a nucleophilic site, did not participate in making a C–C bond with **3a**, leading possibly to **Me_2_-12ea**. As a result, **Me_2_-2e** was recovered quantitatively, even under the standard heating reaction conditions [Equation (3) in Scheme 3]. Accordingly, these results strongly suggest that the annulation reaction proceeds in the order of the S_N_Ar-based intermolecular N–C bond formation, followed by the S_E_Ar-based intramolecular C–C bond formation. Experimental procedures for Equations (1) and (2) as well as spectral and analytical data (melting point, NMR, and HRMS), and NMR charts for products **6ea** and **4ea** are provided in Supplementary Materials.

**Scheme 4:**
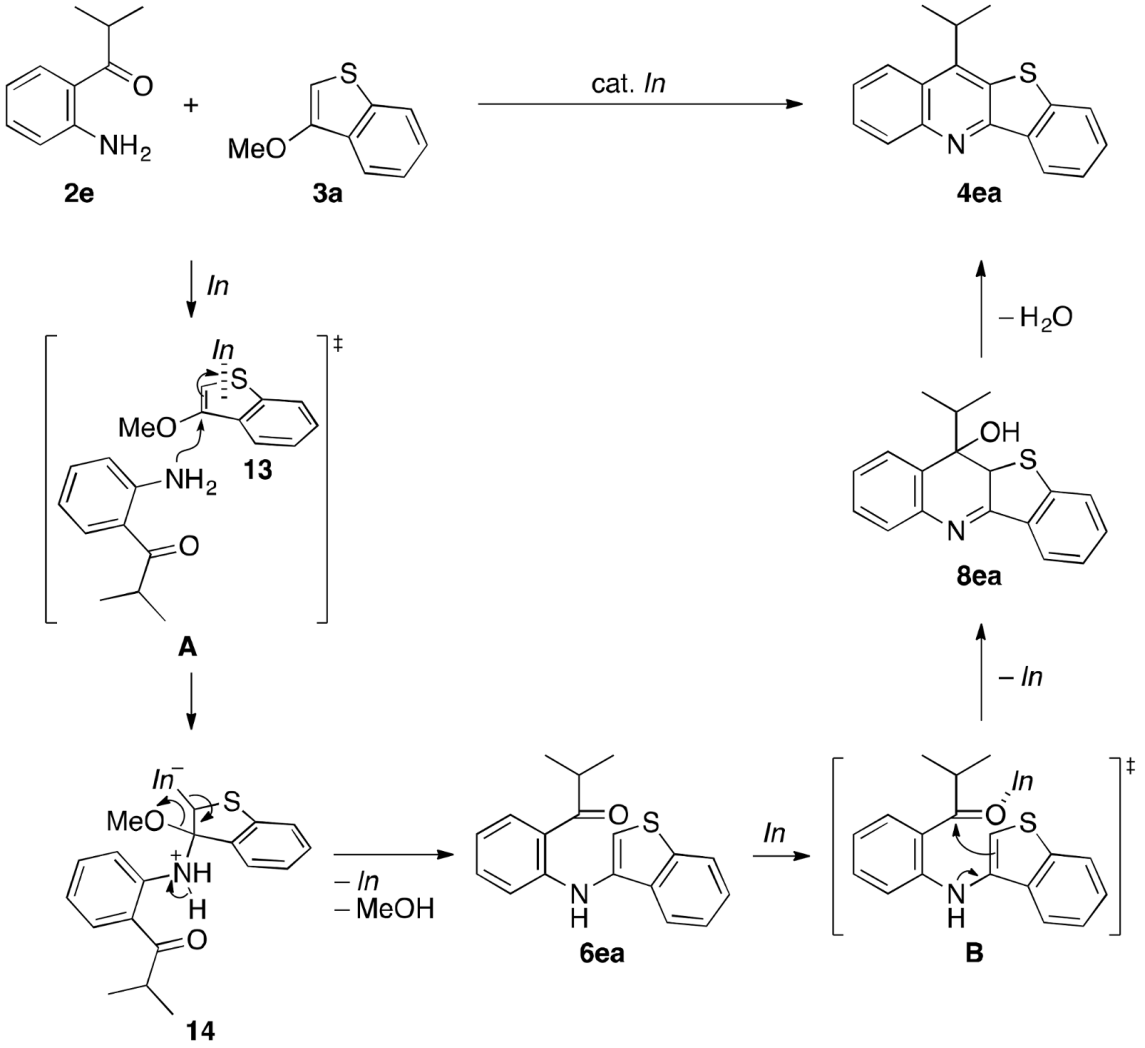
A proposed reaction mechanism.

On the basis of the above experimental results as well as the previous ones, a proposed reaction mechanism is illustrated in Scheme 4 that exemplifies the reaction of **2e** with **3a**. First up is the S_N_Ar-based intermolecular amination of **3a** by the nucleophilic attack of the nitrogen atom of **2e** via previously proposed transition state **A** [54], followed by the release of the indium catalyst (*In*) and MeOH to give intermediate **6ea**. Next is the nucleophilic attack of the thienyl ring to the carbonyl moiety activated by *In* as shown in transition state **B**, hereby providing **8ea**, and then desired structure **4ea** after aromatizing dehydration. The ring-closing C–C bond-forming process might be accelerated by the electron flow from the lone pair on the nitrogen atom. However, due to the fact that **6ea** is the only intermediate confirmed during the annulation process [Equation (1) in Scheme 3], the rate-determining step is likely to be present at the intramolecular C–C bond-forming stage. 

## 3. Materials and Methods

### 3.1. General Remarks

All manipulations were conducted with a standard Schlenk technique under an argon atmosphere. Nuclear magnetic resonance (NMR) spectra were taken on a JEOL JMN-ECA 400 (^1^H, 400 MHz; ^13^C, 100 MHz; ^19^F, 376 MHz) or JEOL JMN-ECA 500 (^1^H, 500 MHz; ^13^C, 125 MHz; ^19^F, 471 MHz) spectrometer (JEOL, Tokyo, Japan) using tetramethylsilane (^1^H and ^13^C) or trichlorofluoromethane (^19^F) as an internal standard. Analytical gas chromatography (GC) was performed on a Shimadzu model GC-2014 instrument with a flame ionization detector (Shimadzu, Kyoto, Japan), equipped with a capillary column of InertCap 5 (5% diphenyl- and 95% dimethylpolysiloxane, 30 m × 0.25 mm × 0.25 µm) (GL Sciences, Tokyo, Japan), using nitrogen as carrier gas. Gas chromatography-mass spectrometry (GC-MS) analyses were performed with a Shimadzu model GCMS-QP2010 instrument (Shimadzu, Kyoto, Japan) equipped with a capillary column of InertCap 5 by electron ionization at 70 eV using helium as the carrier gas. High-resolution mass spectra (HRMS) were obtained with a JEOL JMS-T100GCV spectrometer (JEOL, Tokyo, Japan). All of the melting points were measured with a Yanaco Micro Melting Point MP-500P apparatus (Yanaco, Kyoto, Japan), and are uncorrected. Kugelrohr bulb-to-bulb distillation was carried out with a Sibata glass tube oven GTO-250RS apparatus (Sibata Scientific Technology, Soka, Japan). Chlorobenzene (PhCl), toluene (PhMe), and dichloromethane (CH_2_Cl_2_) were distilled under argon from CaCl_2_ just prior to use. Dibutyl ether (Bu_2_O) and 1,4-dioxane were distilled under argon from sodium just prior to use. 1,2-Diethoxyethane, nitromethane (MeNO_2_), butanol (BuOH) and *o*-dichlorobenzene (*o*-C_6_H_4_Cl_2_) were stored over molecular sieves 4Å (MS 4Å) under argon. Butyronitrile (PrCN) was distilled under argon from P_2_O_5_ just prior to use. MeOH was stored over molecular sieves 3Å (MS 3Å) under argon. The following indium salts and substrates were synthesized according to the respective literature methods: In(NTf_2_)_3_ [83,84], In(ONf)_3_ [56,85], 1-(2-aminophenyl)-2-methyl-1-propanone (**2e**) [86], 1-(2-aminophenyl)-2,2,2-trifluoroethanone (**2f**) [87], (2-aminophenyl)(4-methoxyphenyl)methanone (**2j**) [88], (2-aminophenyl)(2-methylphenyl)methanone (**2l**) [88], 3-methoxybenzo[*b*]thiophene (**3a**) [89], 2-methoxybenzo[*b*]thiophene (**3b**) [54], 2-methoxy-5-methylthiophene (**3d**) [54], 2-methoxybenzo[*b*]furan (**3f**) [54], 2-methoxy-5-phenylfuran (**3g**) [54], 2-methoxy-1-phenyl-1*H*-pyrrole (**3h**) [54]. Unless otherwise noted, other substrates and reagents were commercially available, and used as received without further purification.

### 3.2. Synthesis of Substrates

#### 3.2.1. Synthesis of 1-(2-Amino-5-chlorophenyl)-2,2,2-trifluoroethanone (**2g**): Removal of HCl and H_2_O from **2g**–HCl–H_2_O

A hydrochloride–hydrate adduct of **2g** (407 mg, 1.46 mmol) was placed in a 15-mL screw-cap vial. To this, a saturated NaHCO_3_ aqueous solution (2.0 mL) was added, and the resulting mixture was stirred at room temperature for 3 min. The aqueous phase was extracted with EtOAc (5 mL × 3). The combined organic layer was washed with brine (2 mL) and then dried over anhydrous sodium sulfate (Na_2_SO_4_). Filtration and evaporation of the solvent left a residue, which was successively passed through a pad of silica gel using EtOAc to give analytically pure **2g** in 99% yield (324 mg) as a yellow solid (m.p. 92–94 °C). Compound **2g** has already appeared in the literature [87], and its spectral and analytical data are in good agreement with those reported. Accordingly, only the ^1^H-NMR data are provided here. ^1^H-NMR (400 MHz, CDCl_3_) δ 6.47 (bs, 2H), 6.70 (d, *J* = 8.9 Hz, 1H), 7.33 (dd, *J* = 9.0, 2.4 Hz, 1H), 7.66–7.75 (m, 1H).

#### 3.2.2. Synthesis of 1-[2-(Dimethylamino)phenyl]-2-methyl-1-propanone (**Me_2_-2e**)

On the basis of the literature procedure that has been used when dimethylating closely related 1-(2-aminophenyl)ethanone derivatives [90], **Me_2_-2e** was prepared using the following reagents and conditions: **2e** (163 mg, 1.00 mmol), MeI (426 mg, 3.00 mmol), K_2_CO_3_ (346 mg, 2.50 mmol), *N*,*N*-dimethylformamide (0.60 mL), 80 °C, 8 h, and was isolated by column chromatography on silica gel (*n*-hexane/EtOAc = 30/1) in 71% yield (136 mg) as a pale yellow oil. Compound **Me_2_-2e** has already appeared in the literature [91], and its spectral and analytical data are in good agreement with those reported. Accordingly, only the ^1^H-NMR data are provided here. ^1^H-NMR (500 MHz, CDCl_3_) δ 1.12 (d, *J* = 6.9 Hz, 6H), 2.76 (s, 6H), 3.66 (sept, *J* = 6.9 Hz, 1H), 6.95 (td, *J* = 7.4, 0.9 Hz, 1H), 7.00 (dd, *J* = 8.3, 0.6 Hz, 1H), 7.28 (dd, *J* = 7.7, 1.7 Hz, 1H), 7.34 (ddd, *J* = 8.6, 7.3, 1.7 Hz, 1H).

### 3.3. Indium-Catalyzed Annulation of o-Acylanilines with Alkoxyheteroarenes: An Experimental Procedure Exemplified by the Synthesis of ***4aa***

InBr_3_ (4.43 mg, 12.5 µmol) was placed in a 20-mL Schlenk tube, which was heated at 80 °C in vacuo for 15 min. The tube was cooled down to room temperature, and filled with argon. PhCl (0.20 mL) was added to the tube, and the mixture was then stirred at room temperature for 3 min. To this, 3-methoxybenzothiophene (**3a**) (49.3 mg, 0.300 mmol) and 1-(2-aminophenyl)ethanone (**2a**) (33.8 mg, 0.250 mmol) were added in that order, and the mixture was stirred at 110 °C for 24 h, followed by adding a saturated NaHCO_3_ aqueous solution (0.5 mL). The resulting mixture was stirred for 20 min, and the aqueous phase was then extracted with EtOAc (5 mL × 3). The combined organic layer was washed with brine (1 mL), and then dried over anhydrous sodium sulfate (Na_2_SO_4_). Filtration and evaporation of the solvent followed by column chromatography on silica gel (n-hexane/EtOAc = 10/1) gave 11-methyl[1]benzothieno[3,2-*b*]quinoline (**4aa**) in 90% yield (56.1 mg) as a pale yellow solid (m.p. 145–146 °C). Compound **4aa** was characterized by ^1^H- and ^13^C-NMR spectroscopy and HRMS, as follows: ^1^H-NMR (400 MHz, CDCl_3_) δ 2.94 (s, 3H), 7.53–7.59 (m, 1H), 7.59–7.65 (m, 2H), 7.76 (ddd, J = 8.5, 6.9, 1.5 Hz, 1H), 7.87 (dd, J = 7.8, 0.7 Hz, 1H), 8.10–8.16 (m, 1H), 8.27–8.32 (m, 1H), 8.62–8.67 (m, 1H); ^13^C-NMR (100 MHz, CDCl_3_) δ 17.4, 122.8, 123.0, 124.0, 125.1, 125.9, 126.1, 128.5, 129.8, 130.1, 132.0, 135.1, 137.2, 140.7, 146.7, 153.3. HRMS (FD) Calcd for C_16_H_11_NS: M, 249.0612. Found: m/z 249.0619.

Besides a general experimental procedure for the synthesis of compounds **4**, details of the reaction conditions, purification methods, spectral and analytical data (melting point, NMR, and HRMS), and NMR charts for all products **4** in Table 3, Table 4 and Table 5 are provided in Supplementary Materials.

### 3.4. N-Methylation of Indolo[3,2-b]quinolines with MeOTf: An Experimental Procedure Exemplified by the Synthesis of ***10fi***

Compound **10fi** was synthesized based on the modified literature procedure [82], as follows: A flame-dried 20-mL Schlenk tube was charged with **4fi** (28.6 mg, 0.100 mol) and toluene (0.60 mL). The resulting solution was degassed by three freeze-pump-thaw cycles, and the tube was then filled with argon. To this solution, MeOTf (31.2 mg, 0.190 mmol) that had been distilled by Kugelrohr at 90 °C/500 Pa prior to use was added, and the mixture was then stirred at 50 °C for 24 h. The resulting mixture, including a solid product, was filtered, and the solid was washed with Et_2_O (5 mL). The filtrate was concentrated, and the residue was filtered and then washed with Et_2_O (5 mL). This concentration–filtration–washing sequence was repeated once again, and the combined solid was dried in vacuo to give an analytically pure 5-methyl-11-trifluoromethyl-10H-quindolinium 1,1,1-trifluoromethanesulfonate (**10fi**) in 94% yield (42.4 mg) as a yellow solid (mp 281–282 °C). Compound **10fi** was characterized by ^1^H-, ^13^C- and ^19^F-NMR spectroscopy and HRMS, as follows: ^1^H-NMR (500 MHz, dimethyl sulfoxide-d_6_) δ 5.13 (s, 3H), 7.62 (ddd, J = 8.3, 7.2, 1.1 Hz, 1H), 7.97 (dt, J = 8.3, 0.9 Hz, 1H), 8.06 (ddd, J = 8.3, 7.2, 1.1 Hz, 1H), 8.15 (ddd, J = 8.7, 6.8, 0.9 Hz, 1H), 8.28 (ddd, J = 9.0, 6.9, 1.3 Hz, 1H), 8.51–8.60 (m, 1H), 8.89 (d, J = 8.6 Hz, 1H), 8.97 (d, J = 9.2 Hz, 1H), 12.71 (s, 1H); ^13^C-NMR (125 MHz, dimethyl sulfoxide-d_6_) δ 41.7, 113.5, 113.7, 116.8 (q, J = 32.8 Hz), 119.2, 120.6 (q, J = 322.2 Hz), 121.5, 122.5, 123.2 (q, J = 275.9 Hz), 124.2 (q, J = 3.0 Hz), 127.1, 129.3, 130.8 (q, J = 1.2 Hz), 132.0, 135.1, 135.9, 142.7, 147.3; ^19^F-NMR (376 MHz, dimethyl sulfoxide-d_6_) δ –77.3, –53.4. HRMS (FD) Calcd for C_17_H_12_F_3_N_2_: M^+^, 301.0947. Found: m/z 301.0936.

Besides a general experimental procedure for the synthesis of compounds **10**, details of the reaction conditions, spectral and analytical data (melting point, NMR, and HRMS), and NMR charts for all products **10** in Table 6 are provided in Supplementary Materials.

### 3.5. An Experimental Procedure for the Synthesis of ***11fi*** by Neutralizing ***10fi***

Compound **11fi** was synthesized based on the literature procedure [82], as follows: **10fi** (22.5 mg, 0.0500 mmol) was placed in a 15-mL screw-cap vial. To this, a 5 wt % Na_2_CO_3_ aqueous solution (2.0 mL) was added, and the resulting mixture was stirred at 30 °C for 15 min. The aqueous phase was extracted with CHCl_3_ (4 mL × 4), and the combined organic layer was dried over anhydrous sodium sulfate (Na_2_SO_4_). Filtration and evaporation of the solvent followed by column chromatography on silica gel (CHCl_3_/Et_3_N = 50/1) gave 5-methyl-11-trifluoromethyl-5H-quindoline (**11fi**) in 99% yield (14.9 mg) as a dark navy solid [m.p. 251–253 °C (decomp.)]. Compound **11fi** was characterized by ^1^H-, ^13^C- and ^19^F-NMR spectroscopy and HRMS, as follows: ^1^H-NMR (400 MHz, dimethyl sulfoxide-d_6_) δ 5.02 (s, 3H), 7.12 (ddd, J = 8.4, 6.6, 1.1 Hz, 1H), 7.65 (ddd, J = 8.4, 6.8, 1.1 Hz, 1H), 7.72 (dd, J = 8.6, 0.8 Hz, 1H), 8.86 (ddd, J = 8.6, 6.8, 1.0 Hz, 1H), 7.96 (ddd, J = 8.9, 6.9, 1.1 Hz, 1H), 8.47–8.52 (m, 1H), 8.55 (dd, J = 8.5, 0.7 Hz, 1H), 8.70 (d, J = 8.9 Hz, 1H); ^13^C-NMR (100 MHz, dimethyl sulfoxide-d_6_) δ 40.3, 113.6, 116.1 (q, J = 29.2 Hz), 117.4, 117.9, 119.5, 120.1 (q, J = 1.4 Hz), 124.0 (q, J = 3.8 Hz), 125.0 (q, J = 277.3 Hz), 125.7, 125.9, 127.8, 131.6, 132.7, 142.3, 143.8, 162.9; ^19^F-NMR (471 MHz, dimethyl sulfoxide-d_6_) δ –50.8. HRMS (FD) Calcd for C_17_H_11_F_3_N_2_: M, 300.0874. Found: m/z 300.0870.

## 4. Conclusions

We disclosed here that the indium-catalyzed tandem N–C and C–C bond-forming reaction of *o*-acylanilines with MeO–heteroarenes is a practical methodology to synthesize a range of HA[*b*]Qs with tricyclic and tetracyclic [2,3-*b*] and [3,2-*b*] skeletons fused with sulfur-, oxygen-, and nitrogen-based five-membered heteroaryl rings. Indolo[3,2-*b*]quinolines, which are also the frameworks constructed by our method, were readily converted to cryptolepine derivatives that have not yet been prepared. Mechanistic investigations revealed that the central pyridyl ring is constructed by the sequence of the intermolecular N–C bond-formation, followed by the C–C bond-forming ring closure.

## Figures and Tables

**Table 1:**
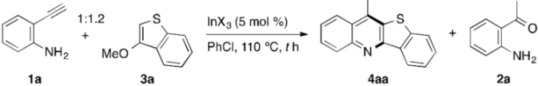
Indium-catalyzed annulation of *o*-ethynylaniline with 3-methoxybenzothiophene *^a^*.

Entry	InX_3_	*t* (h)	Yield of 4aa (%) *^b^*	Yield of 2a (%) *^b^*
1	In(NTf_2_)_3_	24	11	<1
2	In(OTf)_3_	24	9	<1
3	In(ONf)_3_	24	14	2
4	InCl_3_	24	2	<1
5	InBr_3_	24	3	<1
6	InI_3_	24	5	<1
7 *^c^*	In(ONf)_3_	24	26	30
8 *^c^*	In(ONf)_3_	36	61 (61) *^d^*	<1

*^a^* Reagents and conditions (unless otherwise specified): **1a** (0.250 mmol), **3a** (0.300 mmol), InX_3_ (12.5 µmol, 5 mol %), PhCl (0.20 mL), and performed under argon (1 atm). *^b^* Determined by ^1^H-NMR using MeNO_2_ as an internal standard. *^c^* Performed in the presence of H_2_O (1.25 mmol, 5 equiv.). *^d^* The isolated yield of **4aa** is shown in parentheses.

**Table 2:**
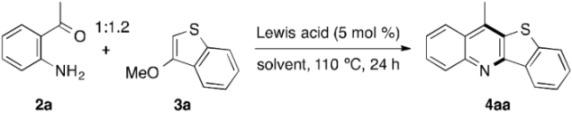
Lewis acid-catalyzed annulation of *o*-acetylaniline with 3-methoxybenzothiophene *^a^*.

Entry	Lewis Acid	Solvent	Conversion of 2a (%) *^b^*	Yield of 4aa (%) *^c^*
1	In(ONf)_3_	PhCl	86	62
2	In(OTf)_3_	PhCl	73	55
3	In(NTf_2_)_3_	PhCl	79	74
4	InF_3_	PhCl	4	<1
5	InCl_3_	PhCl	95	83
6	InBr_3_	PhCl	97	92
7	InI_3_	PhCl	97	92
8	In(OH)_3_	PhCl	<1	<1
9	In(OAc)_3_	PhCl	<1	<1
10	ScBr_3_	PhCl	61	50
11	FeBr_3_	PhCl	86	62
12	CoBr_2_	PhCl	31	22
13	PdBr_2_	PhCl	47	29
14	CuBr_2_	PhCl	28	18
15	AgBr	PhCl	3	<1
16	ZnBr_2_	PhCl	15	5
17	PbBr_2_	PhCl	<1	<1
18	BiBr_3_	PhCl	45	33
19	None	PhCl	5	<1
20	InBr_3_	PhMe	91	82
21	InBr_3_	Bu_2_O	85	74
22	InBr_3_	1,2-Diethoxyethane	91	80
23	InBr_3_	1,4-Dioxane	87	66
24	InBr_3_	MeNO_2_	88	76 *^d^*
25	InBr_3_	PrCN	82	76
26	InBr_3_	BuOH	92	73
27	InBr_3_	H_2_O	48	37
28 *^e^*	InBr_3_	PhCl	89	70
29 *^f^*	InBr_3_	PhCl	97	88

*^a^* Reagents and conditions (unless otherwise specified): **2a** (0.250 mmol), **3a** (0.300 mmol), Lewis acid (12.5 µmol, 5 mol %), solvent (0.20 mL), and performed under argon (1 atm). *^b^* Determined by GC using *n*-dodecane as an internal standard. *^c^* Determined by ^1^H-NMR using MeNO_2_ as an internal standard. *^d^* Determined by ^1^H-NMR using CH_2_Br_2_ instead of MeNO_2_ as an internal standard. *^e^* Performed with InBr_3_ (2.50 µmol, 1 mol %) for 96 h. *^f^* Performed under air (1 atm).

**Table 3:**
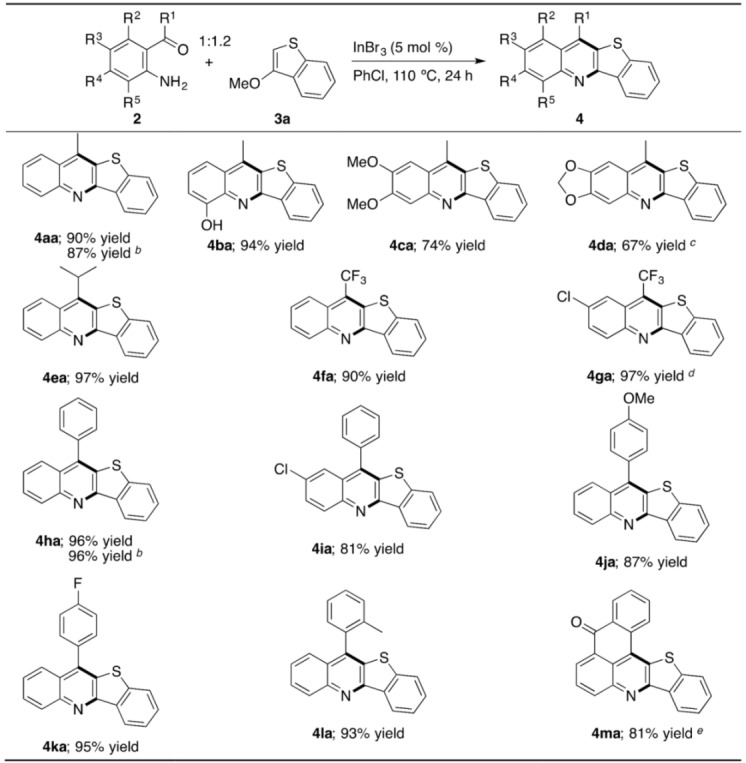
Indium-catalyzed annulation of *o*-acylanilines with 3-methoxybenzothiophene *^a^*.

*^a^* Reagents and conditions (unless otherwise specified): **2** (0.250 mmol), **3a** (0.300 mmol), InBr_3_ (12.5 µmol, 5 mol %), PhCl (0.20 mL), and performed under argon (1 atm). Yields of isolated **4** based on **2** are shown here. *^b^* Yields when performed under air (1 atm). *^c^* Performed in PhCl (0.40 mL) for 36 h. *^d^* Performed with a HCl–H_2_O adduct of **2g**. *^e^* Performed in PhCl (0.50 mL) at 130 °C.

**Table 4:**
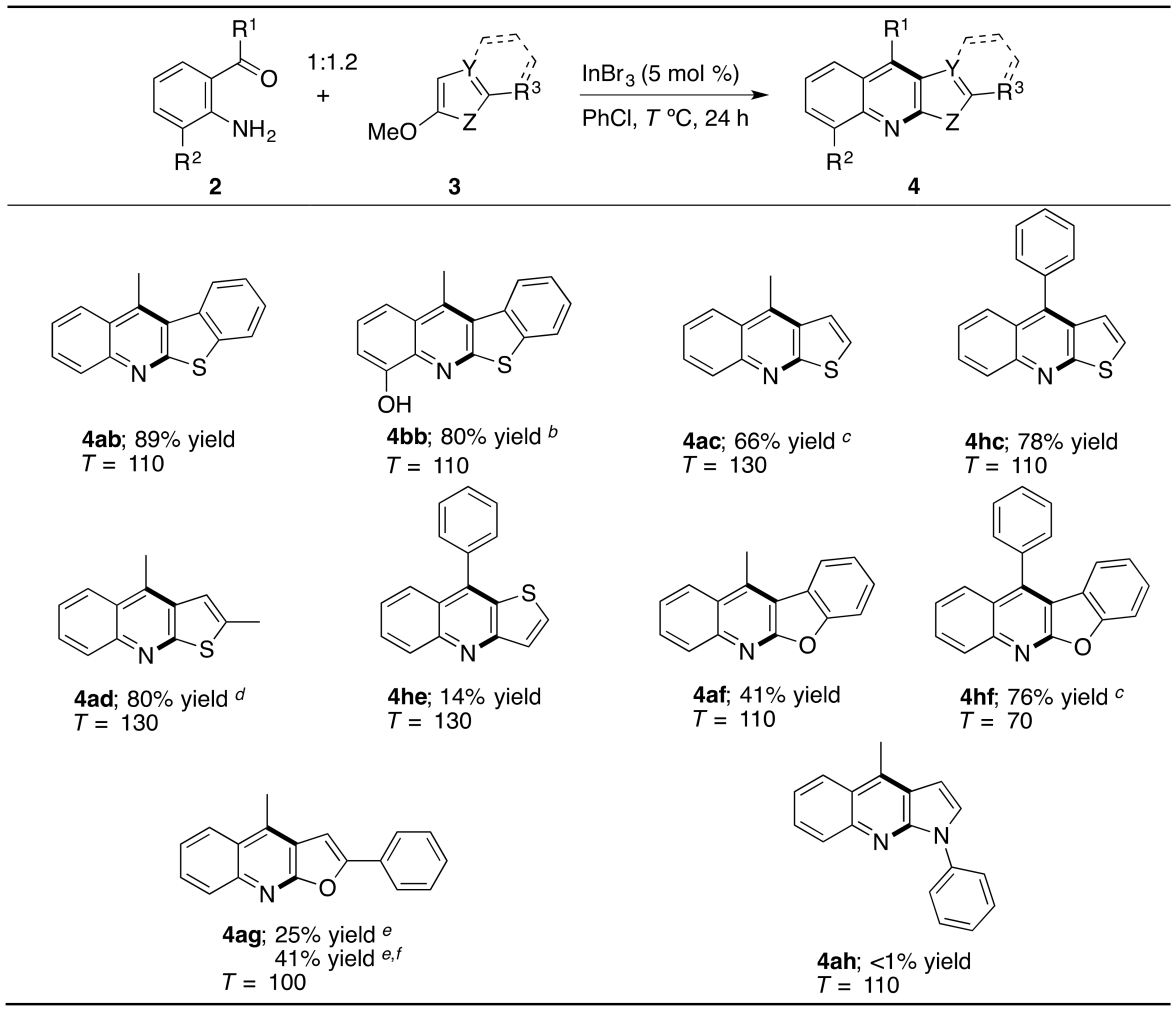
Indium-catalyzed annulation of *o*-acylanilines with methoxyheteroarenes *^a^*.

*^a^* Reagents and conditions (unless otherwise specified): **2** (0.250 mmol), **3** (0.300 mmol), InBr_3_ (12.5 µmol, 5 mol %), PhCl (0.20 mL), and performed under argon (1 atm). Yields of isolated **4** based on **2** are shown here. *^b^* Performed with a 1.2:1 mixture of **2b** (0.300 mmol) and **3b** (0.250 mmol). *^c^* Performed with **3** (0.500 mmol, 2 equiv.). *^d^* Performed with InBr_3_ (37.5 µmol, 15 mol %). *^e^* Performed for 3 h. *^f^* Performed with InI_3_ instead of InBr_3_.

**Table 5:**
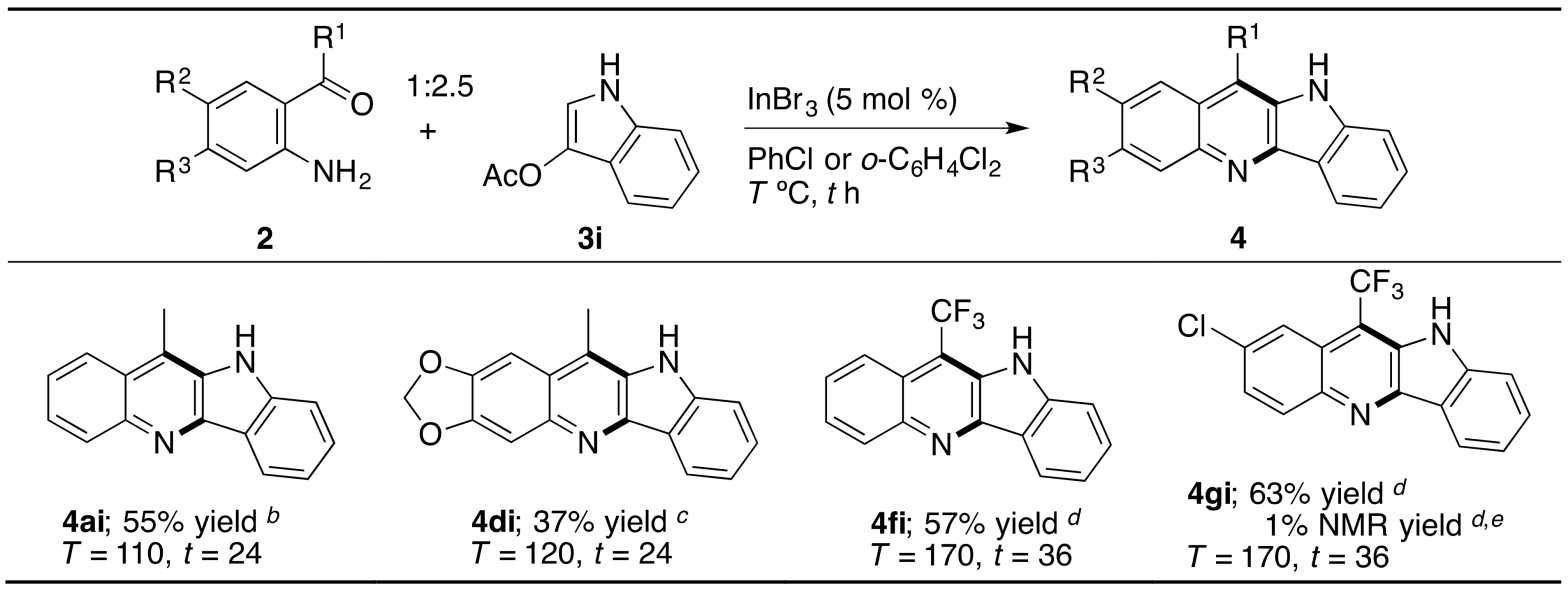
Indium-catalyzed annulation of *o*-acylanilines with 3-acetyloxyindole *^a^*.

*^a^* Reagents and conditions (unless otherwise specified): **2** (0.250 mmol), **3i** (0.625 mmol), InBr_3_ (12.5 µmol, 5 mol %), PhCl or *o*-C_6_H_4_Cl_2_ as a solvent, and performed under argon (1 atm). Yields of isolated **4** based on **2** are shown here. *^b^* Performed on the following larger scale in the presence of H_2_O: **2a** (2.20 mmol), **3i** (5.50 mmol), H_2_O (11.0 mmol), InBr_3_ (110 µmol, 5 mol %), PhCl (1.7 mL). *^c^* Performed in PhCl (0.30 mL). *^d^* Performed in *o*-C_6_H_4_Cl_2_ (0.20 mL). *^e^* Performed with **2g**–HCl–H_2_O as a commercial source instead of **2g**.

**Table 6:**
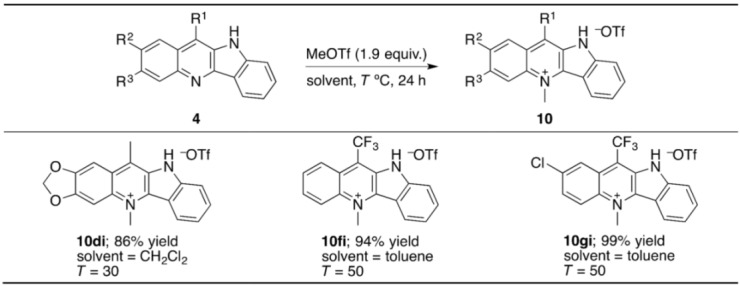
*N*-Methylation of indolo[3,2-*b*]quinolines with MeOTf *^a^*.

*^a^* Reagents: **4** (0.100 mmol), MeOTf (0.190 mmol), solvent [CH_2_Cl_2_ (1.2 mL) or toluene (0.60 mL)]. Yields of isolated **10** based on **4** are shown here.

## Supplementary Materials

Supplementary materials are available online: experimental details for the synthesis of each product as well as ^1^H-, ^13^C- and ^19^F-NMR spectra and HRMS data. References [53,82] are cited in the supplementary materials.
