# Supplementary material for: Indium-Catalyzed Annulation of o-Acylanilines with Alkoxyheteroarenes: Synthesis of Heteroaryl[b]quinolines and Subsequent Transformation to Cryptolepine Derivatives
Source: Molecules. 2018 Apr 5;23(4):838. doi: 10.3390/molecules23040838 (PMC6017974; doi:10.3390/molecules23040838)
Supplement: Supplementary file 1 [file molecules-23-00838-s001.pdf]

Supplementary Materials for

Indium-Catalyzed Annulation of *o*-Acylanilines with Alkoxyheteroarenes: Synthesis of Heteroaryl[*b*]quinolines and Subsequent Transformation to Cryptolepine Derivatives

Kyohei Yonekura, Mika Shinoda, Yuko Yonekura and Teruhisa Tsuchimoto*
Department of Applied Chemistry, School of Science and Technology, Meiji University, 1-1-1 Higashimita, Tama-ku, Kawasaki 214-8571, Japan

A Table of Contents:

I. **Indium-Catalyzed Annulation of *o*-Acylanilines with Alkoxyheteroarenes: A General Procedure for Tables 3–5** S-2

II. **N-Methylation of Indolo[3,2-*b*]quinolines with MeOTf: A General Procedure for Table 6** S-15

III. **Control Experiments for Mechanistic Studies (Scheme 3)**

III-1. **Indium-Catalyzed S_NAr Amination of 3-Methoxybenzothiophene with 1-(2-Aminophenyl)-2-methyl-1-propanone: An Experimental Procedure for Eq. 1 in Scheme 3** S-17

III-2. **Indium-Catalyzed Intramolecular Cyclization of 6ea: An Experimental Procedure for Eq. 2 in Scheme 3** S-18

IV. **^1H, ^{13}C and/or ^{19}F NMR Spectra** S-19

I. Indium-Catalyzed Annulation of *o*-Acylanilines with Alkoxyheteroarenes: A General Procedure for Tables 3–5

$InBr_3$ [(4.43 mg, 12.5 μmol), (13.3 mg, 37.5 μmol) or (39.0 mg, 110 μmol)] or InI_3 (6.19 mg, 12.5 μmol) was placed in a 20 mL Schlenk tube, which was heated at 80 °C in vacuo for 15 min. The tube was cooled down to room temperature and filled with argon or air. PhCl (0.20, 0.30, 0.40, 0.50 or 1.7 mL) or *o*-$C_6H_4Cl_2$ (0.20 mL) was added to the tube, and the mixture was then stirred at room temperature for 3 min. To this were added alkoxyheteroarenes **3** (0.250, 0.300, 0.500, 0.625 or 5.50 mmol) and *o*-acylanilines **2** (0.250, 0.300 or 2.20 mmol) in the order, and the mixture was stirred at 70, 100, 110, 120, 130 or 170 °C. After stirring for 3, 24 or 36 h, a saturated $NaHCO_3$ aqueous solution (0.5 mL) was added at room temperature, and the resulting mixture was stirred for 20 min. The aqueous phase was extracted with EtOAc (5 mL × 3). The combined organic layer was washed with brine (1 mL) and then dried over anhydrous sodium sulfate (Na_2SO_4). Filtration and evaporation of the solvent followed by purification gave product **4**. Unless otherwise noted, the annulation reaction was performed according to the above procedure, and products **4** synthesized here were fully characterized by ^1H and ^{13}C NMR spectroscopy and HRMS. Products **4** with fluorine atoms were characterized additionally by ^{19}F NMR spectroscopy.

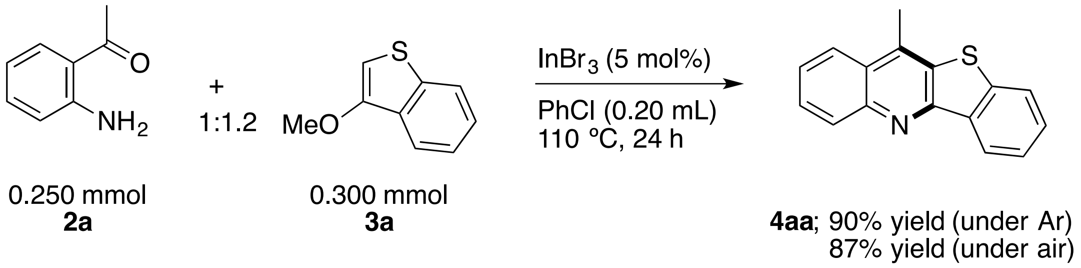

11-Methyl[1]benzothieno[3,2-*b*]quinoline (4aa). Spectral and analytical data of product **4aa** are collected in section 3.3. of the article.

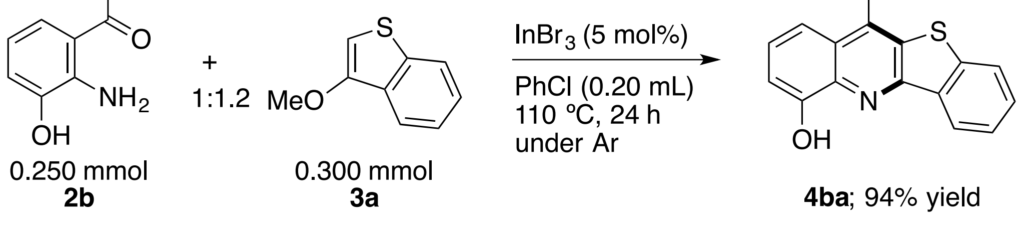

4-Hydroxy-11-methyl[1]benzothieno[3,2-*b*]quinoline (4ba). The title compound was isolated by column chromatography on silica gel (*n*-hexane/EtOAc = 20/1). A pale yellow solid, mp 155–156 ºC. ^{1}H NMR (400 MHz, $CDCl_3$) δ 2.89 (s, 3 H), 7.19–7.24 (m, 1 H), 7.47–7.65 (m, 4 H), 7.82–7.90 (m, 1 H), 8.52–8.63 (m, 2 H); ^{13}C NMR (100 MHz, $CDCl_3$) δ 17.5, 108.6, 113.3, 123.1, 123.8, 125.1, 126.2, 126.9, 129.8, 132.9, 134.5, 136.8, 138.0, 140.7, 150.8, 152.5. HRMS (FD) Calcd for $C_{16}H_{11}NOS$: M, 265.0561. Found: *m*/*z* 265.0554.

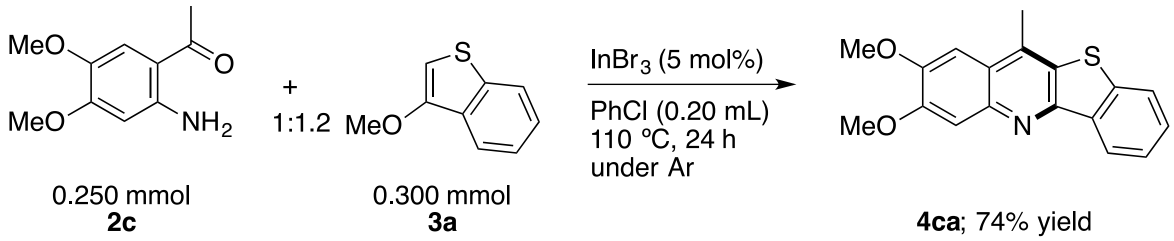

2,3-Dimethoxy-11-methyl[1]benzothieno[3,2-*b*]quinoline (4ca). The title compound was isolated by column chromatography on silica gel (*n*-hexane/EtOAc = 2/1). A yellow solid, mp 191–192 ºC. ^{1}H NMR (400 MHz, $CDCl_3$) δ 2.88 (s, 3 H), 4.09 (s, 3 H), 4.11 (s, 3 H), 7.25–7.27 (m, 1 H), 7.51–7.62 (m, 3 H), 7.83–7.88 (m, 1 H), 8.55–8.60 (m, 1 H); ^{13}C NMR (100 MHz, $CDCl_3$) δ 17.6, 56.0, 56.2, 100.5, 108.2, 121.9, 123.0, 123.3, 124.9, 129.0, 130.7, 135.28, 135.31, 140.1, 144.0, 149.6, 151.3, 151.9. HRMS (FD) Calcd for $C_{18}H_{15}NO_2S$: M, 309.0823. Found: *m*/*z* 309.0808.

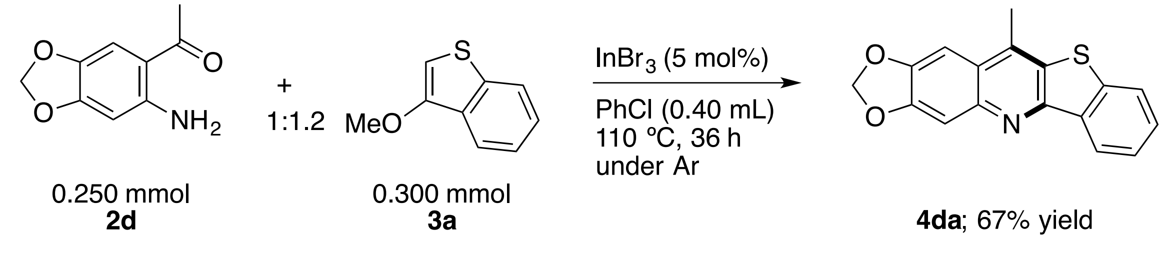

11-Methyl-1,3-dioxolo[4,5-*g*][1]benzothieno[3,2-*b*]quinoline (4da). $CHCl_3$ instead of EtOAc was used to extract an aqueous phase in a work-up process, and the title compound was isolated by column chromatography on silica gel (*n*-hexane/EtOAc = 5/1). A pale yellow solid, mp 224–226 ºC. ^{1}H NMR (500 MHz, $CDCl_3$) δ 2.82 (s, 3 H), 6.15 (s, 2 H), 7.33 (s, 1 H), 7.50–7.62 (m, 3 H), 7.84 (d, J = 7.4 Hz, 1 H), 8.56 (d, J = 7.2 Hz, 1 H); ^{13}C NMR (125 MHz, $CDCl_3$) δ 17.8, 98.3, 101.8, 105.9, 123.0, 123.2, 123.5, 124.9, 129.0, 130.9, 135.2, 135.8, 140.1,

145.2, 147.9, 150.1, 151.2. HRMS (FD) Calcd for $C_{17}H_{11}NO_2S$: M, 293.0510. Found: *m*/*z* 293.0521.

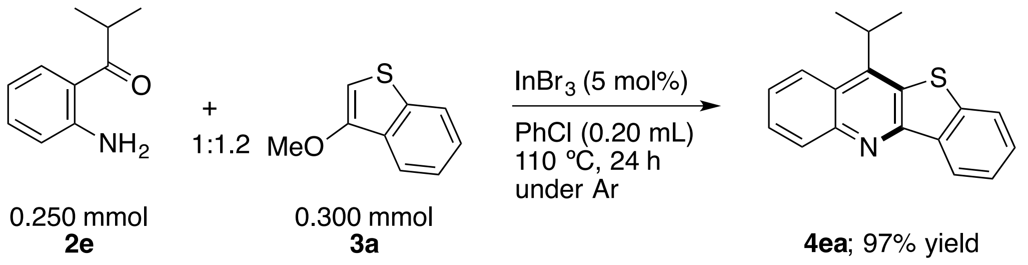

11-(1-Methylethyl)[1]benzothieno[3,2-*b*]quinoline (4ea). $CHCl_3$ instead of EtOAc was used to extract an aqueous phase in a work-up process, and the title compound was isolated by column chromatography on silica gel (*n*-hexane/EtOAc = 40/1). A pale pink solid, mp 109–111 ºC. ^{1}H NMR (400 MHz, $CDCl_3$) δ 1.67 (d, *J* = 7.1 Hz, 6 H), 4.10 (bs, 1 H), 7.51–7.56 (m, 1 H), 7.57–7.63 (m, 2 H), 7.74 (ddd, *J* = 8.4, 6.8, 1.3 Hz, 1 H), 7.81–7.86 (m, 1 H), 8.27–8.34 (m, 2 H), 8.61–8.67 (m, 1 H); ^{1}H NMR (400 MHz, acetone-d_6) δ 1.68 (d, *J* = 7.1 Hz, 6 H), 4.23 (bs, 1 H), 7.62 (ddd, *J* = 8.0, 7.0, 0.9 Hz, 1 H), 7.66–7.74 (m, 2 H), 7.82 (ddd, *J* = 8.4, 6.8, 1.5 Hz, 1 H), 8.03 (dt, *J* = 7.9, 0.8 Hz, 1 H), 8.27 (ddd, *J* = 8.5, 1.4, 0.6 Hz, 1 H), 8.47 (ddd, *J* = 8.7, 0.7, 0.4 Hz, 1 H), 8.60 (ddd, *J* = 7.9, 1.3, 0.6 Hz, 1 H); ^{13}C NMR (100 MHz, $CDCl_3$) δ 20.8, 122.5, 123.1, 123.9, 124.9, 125.7, 128.2, 129.8, 130.5, 134.5, 140.6, 146.7, 147.3, 154.1 (Two aromatic carbon signals are missing due to overlapping, and no methine carbon signal is observed.); ^{13}C NMR (100 MHz, acetone-d_6) δ 21.0, 123.7, 124.20, 124.23, 124.4, 125.7, 126.0, 126.9, 129.3, 131.0, 131.3, 135.3, 141.4, 147.7, 148.3, 154.7 (All aromatic carbon signals are observed, but no methine carbon signal is again observed.). HRMS (FD) Calcd for $C_{18}H_{15}NS$: M, 277.0925. Found: *m*/*z* 277.0913.

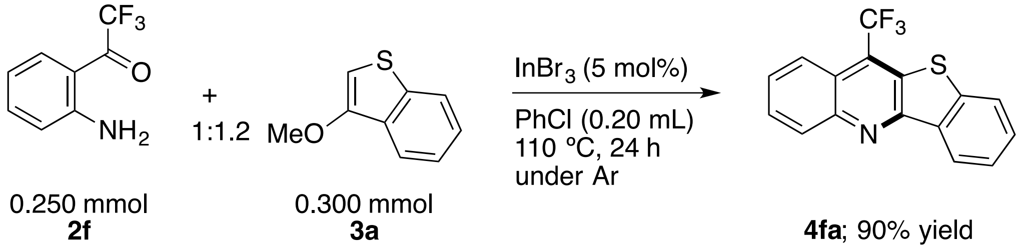

11-Trifluoromethyl[1]benzothieno[3,2-*b*]quinoline (4fa). The title compound was isolated by column chromatography on silica gel (*n*-hexane/EtOAc = 20/1). A pale yellow

solid, mp 141–142 ºC. ^{1}H NMR (400 MHz, $CDCl_3$) δ 7.56–7.62 (m, 1 H), 7.67 (ddd, *J* = 8.1, 7.0, 1.0 Hz, 1 H), 7.72 (ddd, *J* = 8.5, 6.9, 1.3 Hz, 1 H), 7.81–7.88 (m, 2 H), 8.23–8.31 (m, 1 H), 8.35–8.41 (m, 1 H), 8.66 (d, *J* = 7.8 Hz, 1 H); ^{13}C NMR (100 MHz, $CDCl_3$) δ 122.1 (q, *J* = 1.9 Hz), 122.5, 123.3 (q, *J* = 2.9 Hz), 124.1, 124.6 (q, *J* = 276.4 Hz), 125.5, 126.9 (q, *J* = 32.4 Hz), 127.9, 129.2, 129.9 (q, *J* = 1.0 Hz), 130.4, 130.8, 133.1, 140.9 (q, *J* = 3.5 Hz), 146.9, 154.7; ^{19}F NMR (471 MHz, $CDCl_3$) δ –56.5. HRMS (FD) Calcd for $C_{16}H_8F_3NS$: M, 303.0330. Found: *m*/*z* 303.0316.

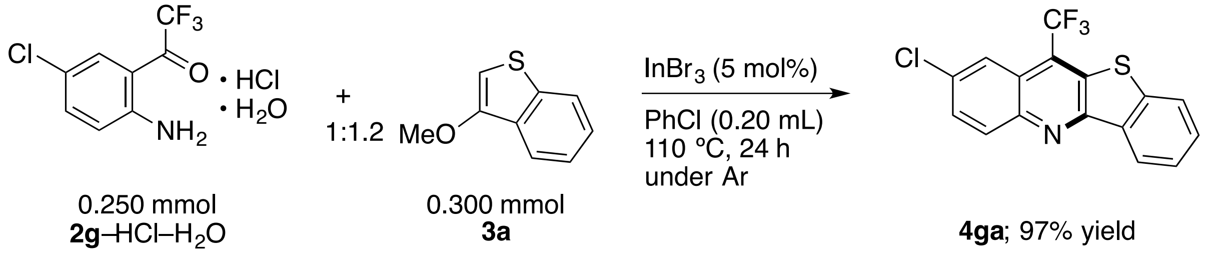

2-Chloro-11-trifluoromethyl[1]benzothieno[3,2-*b*]quinoline (4ga). The title compound was isolated by column chromatography on silica gel (*n*-hexane/EtOAc = 50/1). A pale yellow solid, mp 199–200 ºC. ^{1}H NMR (500 MHz, $CDCl_3$) δ 7.61 (ddd, *J* = 7.9, 7.2, 0.9 Hz, 1 H), 7.70 (ddd, *J* = 7.7, 7.2, 1.3 Hz, 1 H), 7.79 (dd, *J* = 9.2, 2.3 Hz, 1 H), 7.85–7.90 (m, 1 H), 8.22–8.27 (m, 1 H), 8.32 (d, *J* = 8.9 Hz, 1 H), 8.62–8.67 (m, 1 H); ^{13}C NMR (125 MHz, $CDCl_3$) δ 122.3 (q, *J* = 3.0 Hz), 122.5, 122.6, 124.1, 124.2 (q, *J* = 276.1 Hz), 125.7, 126.0 (q, *J* = 33.0 Hz), 130.3, 130.9, 131.0, 131.8, 132.8, 134.0, 140.8 (q, *J* = 3.4 Hz), 145.2, 154.9; ^{19}F NMR (471 MHz, $CDCl_3$) δ –56.7. HRMS (FD) Calcd for $C_{16}H_7ClF_3NS$: M, 336.9940. Found: *m*/*z* 336.9944.

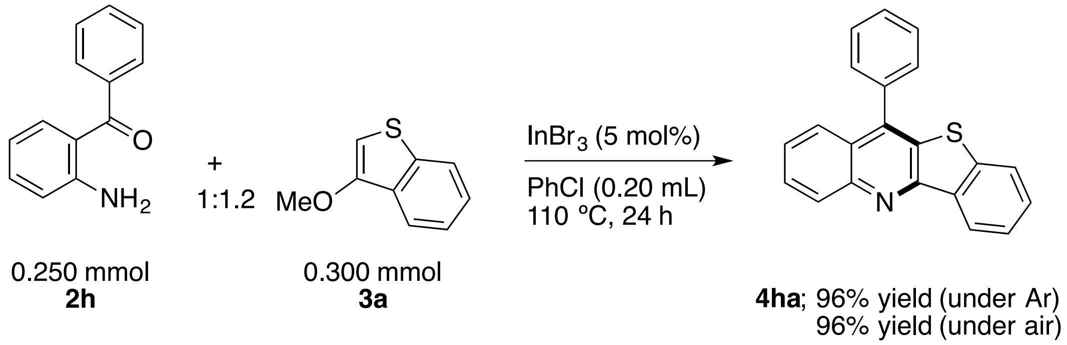

11-Phenyl[1]benzothieno[3,2-*b*]quinoline (4ha). $CHCl_3$ instead of EtOAc was used

to extract an aqueous phase in a work-up process, and the title compound was isolated by column chromatography on silica gel (*n*-hexane/$CHCl_3$ = 1/30). Compound **4ha** has already emerged in the literature [1], and its spectral and analytical data are in good agreement with those reported. Accordingly, only ^{1}H NMR data are provided here. ^{1}H NMR (400 MHz, $CDCl_3$) δ 7.51 (ddd, *J* = 8.4, 6.9, 1.2 Hz, 1 H), 7.54–7.64 (m, 7 H), 7.77 (dd, *J* = 8.5, 7.1, 1.3 Hz, 2 H), 7.83–7.89 (m, 1 H), 8.32–8.39 (m, 1 H), 8.66–8.72 (m, 1 H).

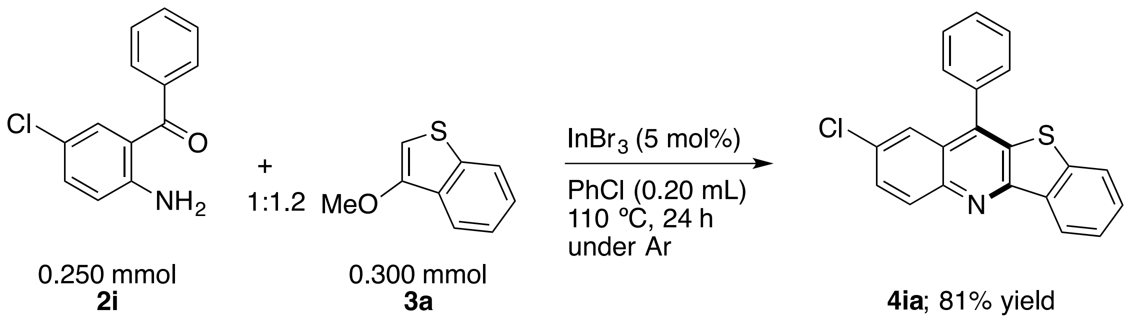

2-Chloro-11-phenyl[1]benzothieno[3,2-*b*]quinoline (4ia). $CHCl_3$ instead of EtOAc was used to extract an aqueous phase in a work-up process, and the title compound was isolated by column chromatography on silica gel (*n*-hexane/$CHCl_3$ = 1/1). A white solid, mp 228–229 ºC. ^{1}H NMR (400 MHz, $CDCl_3$) δ 7.55–7.68 (m, 7 H), 7.69 (dd, *J* = 8.9, 2.3 Hz, 1 H), 7.76–7.80 (m, 1 H), 7.81 (d, *J* = 2.3 Hz, 1 H), 8.28 (d, *J* = 9.2 Hz, 1 H), 8.63–8.68 (m, 1 H); ^{13}C NMR (100 MHz, $CDCl_3$) δ 122.9, 123.7, 124.1, 125.2, 125.9, 129.2, 129.29, 129.32, 129.6, 130.1, 131.3, 132.0, 133.1, 134.5, 136.0, 140.7, 141.6, 145.6, 153.8. HRMS (FD) Calcd for $C_{21}H_{12}ClNS$: M, 345.0379. Found: *m*/*z* 345.0357.

1. Gao, W.-C.; Liu, T.; Cheng, Y.-F.; Chang, H.-H.; Li, X.; Zhou, R.; Wei, W.-L.; Qiao, Y. *J. Org. Chem.* **2017**, *82*, 13459–13467.

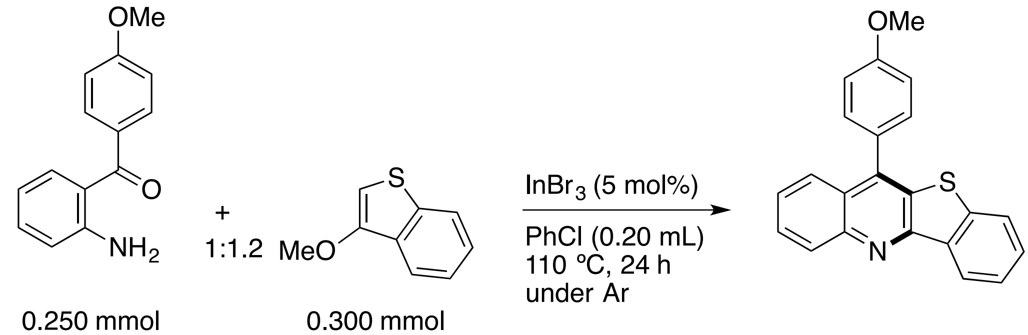

0.250 mmol **2j** 0.300 mmol **3a** **4ja**; 87% yield

11-(4-Methoxyphenyl)[1]benzothieno[3,2-*b*]quinoline (4ja). $CHCl_3$ instead of EtOAc was used to extract an aqueous phase in a work-up process, and the title compound was isolated by column chromatography on silica gel (*n*-hexane/$CHCl_3$ = 1/3). A white solid, mp 200–204 ºC. ^{1}H NMR (400 MHz, $CDCl_3$) δ 3.94 (s, 3 H), 7.15 (dt, *J* = 9.3, 2.5 Hz, 2 H), 7.51 (ddd, *J* = 8.4, 6.8, 1.3 Hz, 1 H), 7.54–7.62 (m, 4 H), 7.73–7.81 (m, 2 H), 7.90 (dd, *J* = 8.5, 0.9 Hz, 1 H), 8.34 (d, *J* = 8.2 Hz, 1 H), 8.64–8.75 (m, 1 H); ^{13}C NMR (100 MHz, $CDCl_3$) δ 55.4, 114.4, 122.9, 124.1, 125.01, 125.03, 125.6, 126.0, 128.6, 128.8, 129.7, 129.8, 130.8, 132.3, 134.8, 141.5, 141.6, 147.3, 153.5, 160.1. HRMS (FD) Calcd for $C_{22}H_{15}NOS$: M, 341.0874. Found: *m*/*z* 341.0879.

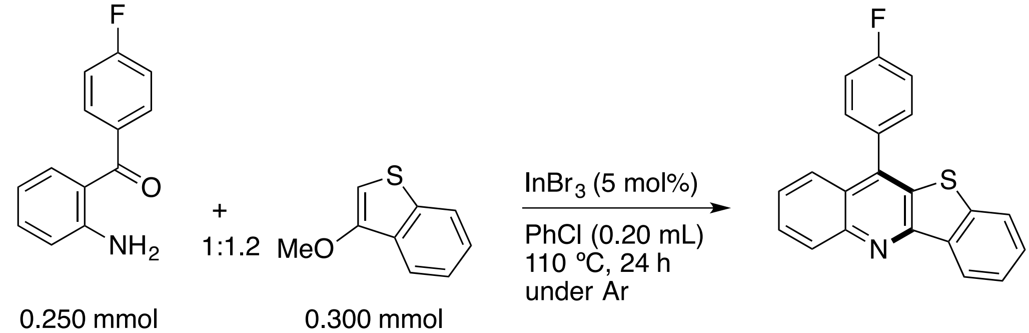

0.250 mmol **2k** 0.300 mmol **3a** **4ka**; 95% yield

11-(4-Fluorophenyl)[1]benzothieno[3,2-*b*]quinoline (4ka). $CHCl_3$ instead of EtOAc was used to extract an aqueous phase in a work-up process, and the title compound was isolated by column chromatography on silica gel (*n*-hexane/EtOAc = 10/1). A white solid, mp 182–183 ºC. ^{1}H NMR (400 MHz, $CDCl_3$) δ 7.29–7.36 (m, 2 H), 7.53 (ddd, *J* = 8.5, 6.9, 1.4 Hz, 1 H), 7.56–7.64 (m, 4 H), 7.75–7.84 (m, 3 H), 8.36 (ddd, *J* = 8.7, 1.2, 0.6 Hz, 1 H), 8.66–8.72 (m, 1 H); ^{13}C NMR (100 MHz, $CDCl_3$) δ 116.2 (d, *J* = 21.6 Hz), 122.9, 124.1, 124.7, 125.2, 125.4, 126.3, 128.8, 129.8, 129.9, 131.4 (d, *J* = 8.6 Hz), 132.3, 132.6 (d, *J* = 3.4 Hz), 134.7, 140.5, 141.4, 147.2, 153.6, 163.1 (d, *J* = 248.7 Hz); ^{19}F NMR (471 MHz, $CDCl_3$) δ –112.5. HRMS

(FD) Calcd for $C_{21}H_{12}FNS$: M, 329.0674. Found: *m*/*z* 329.0671.

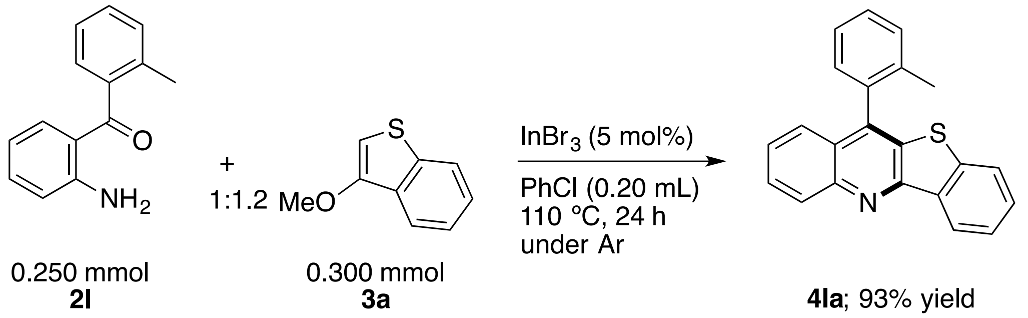

11-(2-Methylphenyl)[1]benzothieno[3,2-*b*]quinoline (4la). $CHCl_3$ instead of EtOAc was used to extract an aqueous phase in a work-up process, and the title compound was isolated by column chromatography on silica gel (*n*-hexane/EtOAc = 10/1). A white solid, mp 175–177 °C. ^{1}H NMR (400 MHz, $CDCl_3$) δ 2.02 (s, 3 H), 7.34 (dd, *J* = 7.4, 1.0 Hz, 1 H), 7.41 (td, *J* = 7.3, 1.7 Hz, 1 H), 7.44–7.52 (m, 3 H), 7.54–7.64 (m, 3 H), 7.75–7.81 (m, 2 H), 8.37 (d, *J* = 8.7 Hz, 1 H), 8.68–8.74 (m, 1 H); ^{13}C NMR (100 MHz, $CDCl_3$) δ 19.6, 123.0, 124.0, 124.9, 125.1, 125.6, 126.2, 126.3, 128.7, 129.1, 129.2, 129.7, 129.9, 130.6, 132.4, 134.8, 136.0, 136.4, 141.5, 147.0, 153.6 (One carbon signal is missing due to overlapping.). HRMS (FD) Calcd for $C_{22}H_{15}NS$: M, 325.0925. Found: *m*/*z* 325.0934.

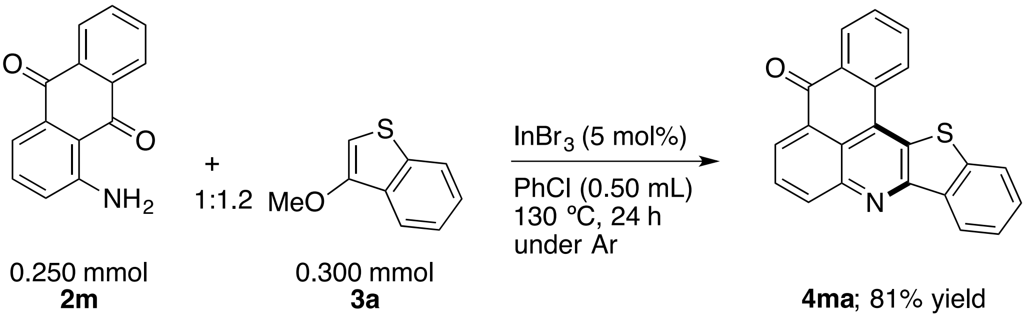

Naphtho[1,2,3-*de*][1]benzothieno[3,2-*b*]quinolin-5(3*H*)-one (4ma). The following work-up procedure was conducted after stirring at 130 °C for 24 h: a saturated $NaHCO_3$ aqueous solution (0.1 mL) was added at room temperature. The resulting mixture was stirred for 20 min and then dried over anhydrous sodium sulfate (Na_2SO_4). The mixture was filtered and eluted with $CHCl_3$ (60 mL). (Note: due to the low solubility of the title compound in $CHCl_3$, a larger amount of $CHCl_3$ should be required.) The solvent was evaporated, and recrystallization from CH_2Cl_2/EtOAc followed by washing with a small amount of EtOAc gave product **4ma**. A

grass green solid, mp 313–316 ºC (decomp.). ^{1}H NMR (400 MHz, $CDCl_3$) δ 7.61 (t, J = 7.1 Hz, 1 H), 7.66–7.74 (m, 2 H), 7.87–7.94 (m, 2 H), 7.97 (t, J = 7.8 Hz, 1 H), 8.59 (d, J = 8.5 Hz, 1 H), 8.62–8.70 (m, 2 H), 8.74 (d, J = 8.2 Hz, 1 H), 8.79 (d, J = 7.1 Hz, 1 H); ^{13}C NMR (100 MHz, $CDCl_3$) δ 122.0, 122.5, 124.2, 125.9, 127.6, 127.8, 128.7, 128.8, 129.0, 129.78, 129.85, 129.9, 130.7, 132.8, 133.3, 133.7, 134.5, 136.9, 140.0, 145.7, 156.0, 182.4. HRMS (FD) Calcd for $C_{22}H_{11}NOS$: M, 337.0561. Found: m/z 337.0574.

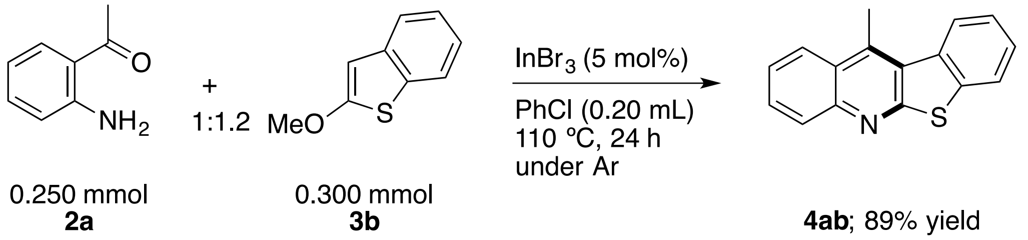

11-Methyl[1]benzothieno[2,3-*b*]quinoline (4ab). The title compound was isolated by column chromatography on silica gel (n-hexane/EtOAc = 20/1). A white solid, mp 174–175 ºC. ^{1}H NMR (400 MHz, $CDCl_3$) δ 3.25 (s, 3 H), 7.49–7.57 (m, 2 H), 7.61 (ddd, J = 8.5, 6.8, 1.3 Hz, 1 H), 7.77 (ddd, J = 8.4, 6.8, 1.3 Hz, 1 H), 7.86–7.92 (m, 1 H), 8.14 (dd, J = 8.5, 0.7 Hz, 1 H), 8.33 (dd, J = 8.6, 0.8 Hz, 1 H), 8.39–8.45 (m, 1 H); ^{13}C NMR (100 MHz, $CDCl_3$) δ 15.9, 123.2, 124.1, 124.9, 125.3, 125.5, 126.0, 126.7, 127.5, 128.7, 129.4, 134.1, 138.5, 140.4, 146.9, 162.9. HRMS (FD) Calcd for $C_{16}H_{11}NS$: M, 249.0612. Found: m/z 249.0607.

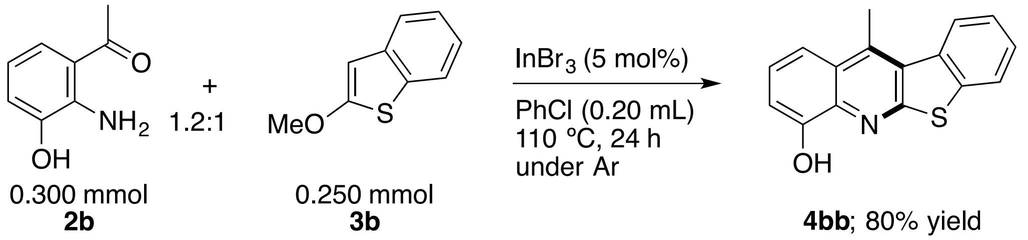

11-Methyl[1]benzothieno[2,3-*b*]quinolin-7-ol (4bb). The title compound was isolated by column chromatography on silica gel (n-hexane/EtOAc = 4/1). A beige solid, mp 206 ºC. ^{1}H NMR (400 MHz, $CDCl_3$) δ 3.23 (s, 3 H), 7.22–7.26 (m, 1 H), 7.48–7.59 (m, 3 H), 7.79 (dd, J = 8.7, 0.9 Hz, 1 H), 7.86–7.94 (m, 1 H), 8.25 (bs, 1 H), 8.41–8.47 (m, 1 H); ^{13}C NMR (100 MHz, $CDCl_3$) δ 16.2, 109.7, 114.6, 123.2, 125.0, 125.8, 126.0, 126.1, 127.4, 127.6, 134.0, 137.3, 138.3, 141.1, 151.4, 160.6. HRMS (FD) Calcd for $C_{16}H_{11}NOS$: M, 265.0561. Found:

m/*z* 265.0586.

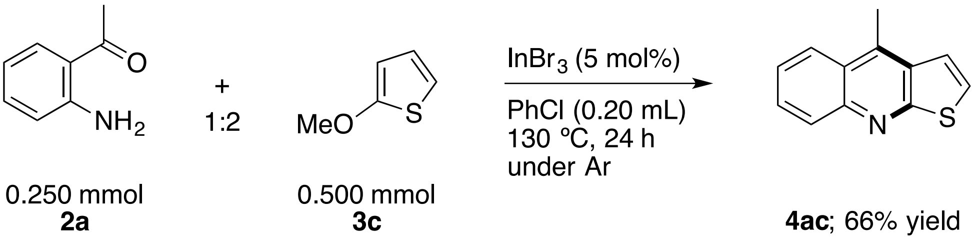

4-Methylthieno[2,3-*b*]quinoline (4ac). The title compound was isolated by column chromatography on silica gel (*n*-hexane/EtOAc = 20/1). A white solid, mp 73–74 ºC. ^{1}H NMR (400 MHz, $CDCl_3$) δ 3.00 (s, 3 H), 7.48 (d, J = 6.2 Hz, 1 H), 7.54–7.62 (m, 2 H), 7.75 (ddd, J = 8.4, 6.8, 1.5 Hz, 1 H), 8.15 (ddd, J = 8.5, 1.2, 0.6 Hz, 1 H), 8.18 (ddd, J = 8.5, 1.4, 0.6 Hz, 1 H); ^{13}C NMR (100 MHz, $CDCl_3$) δ 15.4, 120.0, 124.2, 124.8, 125.1, 127.4, 128.9, 129.1, 130.8, 138.6, 146.5, 162.9. HRMS (FD) Calcd for $C_{12}H_9NS$: M, 199.0456. Found: *m*/*z* 199.0462.

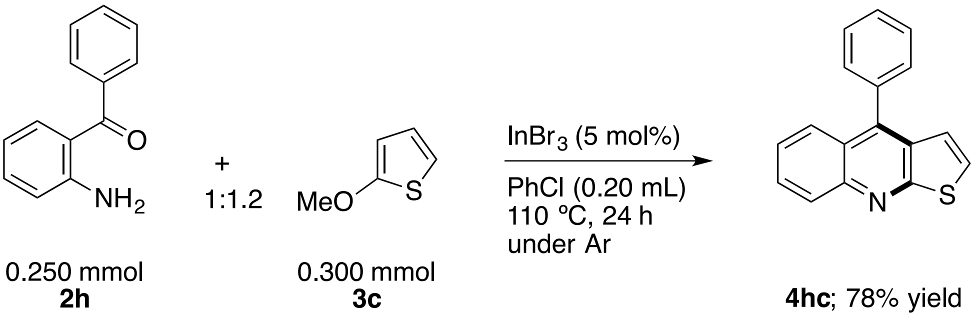

4-Phenylthieno[2,3-*b*]quinoline (4hc). The title compound was isolated by column chromatography on silica gel (*n*-hexane/EtOAc = 10/1). A pale gray solid, mp 116–118 ºC. ^{1}H NMR (400 MHz, $CDCl_3$) δ 7.11 (d, J = 6.2 Hz, 1 H), 7.45–7.62 (m, 7 H), 7.75 (ddd, J = 8.6, 6.8, 1.5 Hz, 1 H), 7.87 (ddd, J = 8.5, 1.4, 0.6 Hz, 1 H), 8.20 (ddd, J = 8.5, 1.2, 0.6 Hz, 1 H); ^{13}C NMR (125 MHz, $CDCl_3$) δ 121.3, 123.9, 125.4, 126.4, 127.9, 128.5, 128.60, 128.65, 129.1, 130.0, 130.3, 136.1, 142.8, 146.9, 163.0. HRMS (FD) Calcd for $C_{17}H_{11}NS$: M, 261.0612. Found: *m*/*z* 261.0615.

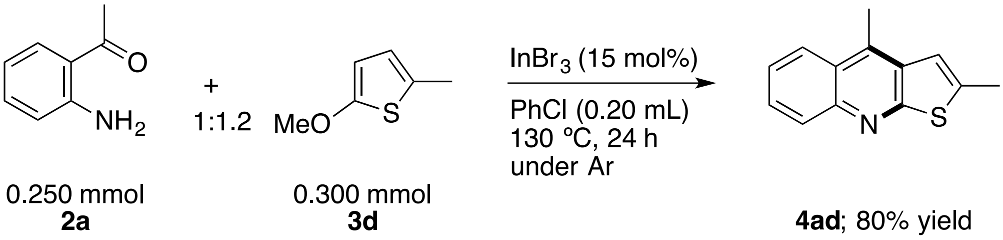

2,4-Dimethylthieno[2,3-*b*]quinoline (4ad). The title compound was isolated by column chromatography on silica gel (*n*-hexane/EtOAc/Et_3N = 20/1/1). A white solid, mp 91–92 ºC. ^{1}H NMR (500 MHz, $CDCl_3$) δ 2.66 (d, *J* = 1.1 Hz, 3 H), 2.91 (s, 3 H), 7.10 (q, *J* = 1.3 Hz, 1 H), 7.55 (ddd, *J* = 8.2, 6.9, 1.1 Hz, 1 H), 7.70 (ddd, *J* = 8.5, 7.1, 1.1 Hz, 1 H), 8.08–8.15 (m, 2 H); ^{13}C NMR (125 MHz, $CDCl_3$) δ 15.3, 17.4, 117.3, 124.1, 125.0, 125.1, 128.4, 128.9, 132.3, 136.5, 141.8, 145.9, 163.5. HRMS (FD) Calcd for $C_{13}H_{11}NS$: M, 213.0612. Found: *m*/*z* 213.0622.

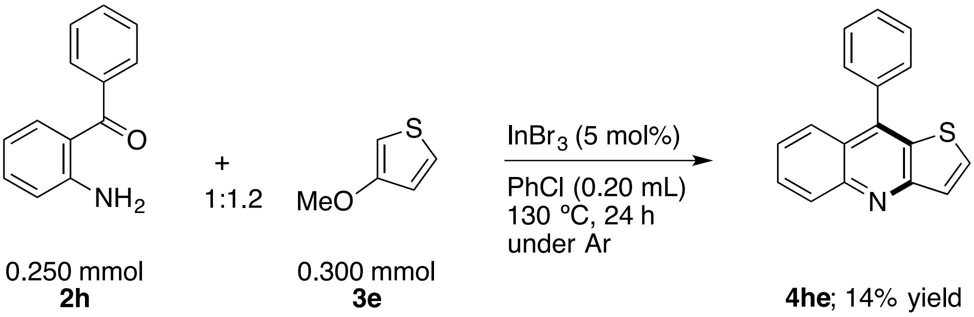

9-Phenylthieno[3,2-*b*]quinoline (4he). The title compound was isolated by column chromatography on silica gel (*n*-hexane/EtOAc = 10/1). A pale yellow solid, mp 116–118 ºC. ^{1}H NMR (400 MHz, $CDCl_3$) δ 7.49 (ddd, *J* = 8.6, 6.8, 1.3 Hz, 1 H), 7.56–7.64 (m, 5 H), 7.68 (d, *J* = 5.7 Hz, 1 H), 7.76 (ddd, *J* = 8.6, 6.8, 1.5 Hz, 1 H), 7.87 (ddd, *J* = 8.6, 1.4, 0.6 Hz, 1 H), 7.93 (d, *J* = 5.7 Hz, 1 H), 8.26 (ddd, *J* = 8.6, 1.1, 0.6 Hz, 1 H); ^{13}C NMR (125 MHz, $CDCl_3$) δ 123.5, 125.2, 125.3, 125.6, 128.96, 128.99, 129.04, 129.4, 129.5, 131.6, 135.2, 136.5, 142.4, 147.7, 157.1. HRMS (FD) Calcd for $C_{17}H_{11}NS$: M, 261.0612. Found: *m*/*z* 261.0615.

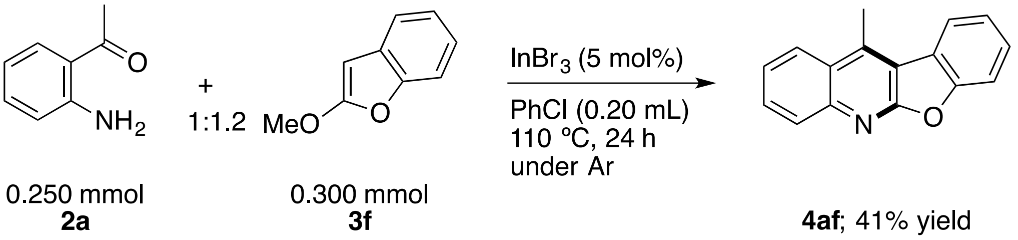

11-Methylbenzofuro[2,3-*b*]quinoline (4af). The title compound was isolated by column chromatography on silica gel (*n*-hexane/EtOAc = 20/1). A beige solid, mp 194–195 ºC. ^{1}H NMR (400 MHz, $CDCl_3$) δ 3.16 (s, 3 H), 7.39–7.46 (m, 1 H), 7.52–7.67 (m, 3 H), 7.77 (ddd, *J* = 8.2, 6.9, 1.1 Hz, 1 H), 8.12–8.16 (m, 2 H), 8.22 (dd, *J* = 8.5, 0.9 Hz, 1 H); ^{13}C NMR (100 MHz, $CDCl_3$) δ 15.2, 112.0, 116.0, 123.2, 123.3, 123.8, 124.8, 126.0, 128.6, 129.1, 129.4, 140.7, 145.9, 155.6, 162.1 (One carbon signal is missing due to overlapping.). HRMS (FD) Calcd for $C_{16}H_{11}NO$: M, 233.0841. Found: *m*/*z* 233.0842.

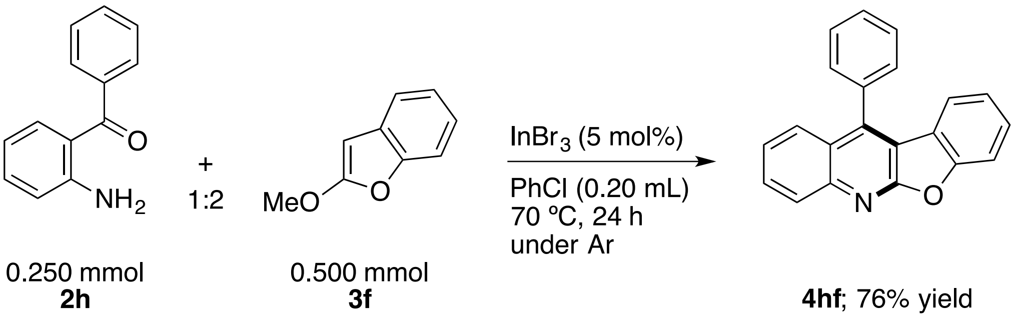

11-Phenylbenzofuro[2,3-*b*]quinoline (4hf). The title compound was isolated by column chromatography on silica gel (*n*-hexane/EtOAc = 20/1). A white solid, mp 216–217 ºC. ^{1}H NMR (400 MHz, $CDCl_3$) δ 7.06–7.10 (m, 1 H), 7.13 (td, *J* = 7.4, 0.8 Hz, 1 H), 7.44–7.51 (m, 2 H), 7.52–7.58 (m, 2 H), 7.58–7.63 (m, 1 H), 7.63–7.72 (m, 3 H), 7.77 (ddd, *J* = 8.5, 6.9, 1.4 Hz, 1 H), 7.81 (ddd, *J* = 8.5, 1.4, 0.7 Hz, 1 H), 8.21 (dd, *J* = 8.5, 0.7 Hz, 1 H); ^{13}C NMR (125 MHz, $CDCl_3$) δ 111.8, 115.6, 122.4, 122.8, 123.1, 125.0, 125.6, 126.1, 128.6, 129.00, 129.02, 129.1, 129.3, 129.5, 135.3, 144.0, 146.2, 155.9, 162.1. HRMS (FD) Calcd for $C_{21}H_{13}NO$: M, 295.0997. Found: *m*/*z* 295.0986.

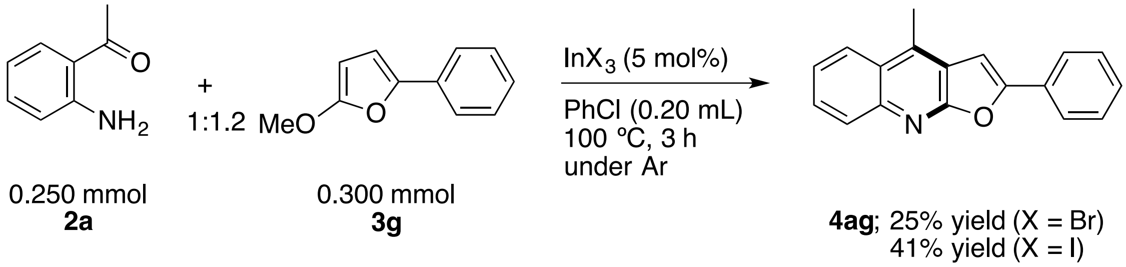

4-Methyl-2-phenylfuro[2,3-*b*]quinoline (4ag). The title compound was isolated by column chromatography on silica gel (*n*-hexane/EtOAc = 10/1). A white solid, mp 133–134 ºC. ^{1}H NMR (400 MHz, $CDCl_3$) δ 2.92 (s, 3 H), 7.19 (s, 1 H), 7.43 (tt, *J* = 7.4, 1.6 Hz, 1 H), 7.48–7.53 (m, 2 H), 7.55 (ddd, *J* = 8.4, 6.9, 1.4 Hz, 1 H), 7.70 (ddd, *J* = 8.4, 6.9, 1.5 Hz, 1 H), 7.97–8.02 (m, 2 H), 8.07–8.14 (m, 2 H); ^{13}C NMR (100 MHz, $CDCl_3$) δ 15.3, 98.7, 121.7, 123.8, 124.6, 125.4, 126.1, 128.5, 129.0, 129.1, 129.5, 129.6, 137.1, 144.9, 156.7, 161.1. HRMS (FD) Calcd for $C_{18}H_{13}NO$: M, 259.0997. Found: *m*/*z* 259.0974.

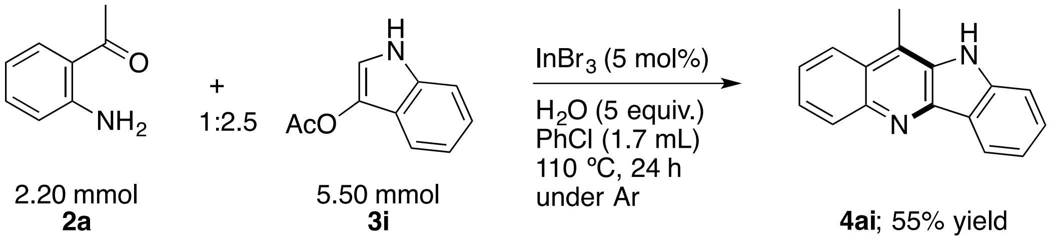

11-Methyl-10*H*-quindoline (4ai). The annulation reaction was conducted after the addition of **3i**, **2a**, and then H_2O (198 mg, 11.0 mmol). The title compound was isolated by column chromatography on silica gel (*n*-hexane/EtOAc/Et_3N = 8/4/1). Compound **4ai** has already emerged in the literature [2], and its spectral and analytical data are in good agreement with those reported. Accordingly, only ^{1}H NMR data are provided here. ^{1}H NMR (500 MHz, $CDCl_3$) δ 2.88 (s, 3 H), 7.32 (t, *J* = 7.6 Hz, 1 H), 7.48 (d, *J* = 8.3 Hz, 1 H), 7.55–7.63 (m, 2 H), 7.68 (t, *J* = 7.4 Hz, 1 H), 8.13 (d, *J* = 8.3 Hz, 1 H), 8.26 (bs, 1 H), 8.34 (d, *J* = 8.3 Hz, 1 H), 8.52 (d, *J* = 7.7 Hz, 1 H).

---

2. Arzel, E.; Rocca, P.; Grellier, P.; Labaeïd, M.; Frappier, F.; Guéritte, F.; Gaspard, C.; Marsais, F.; Godard, A.; Quéguiner, G. *J. Med. Chem.* **2001**, *44*, 949–960.

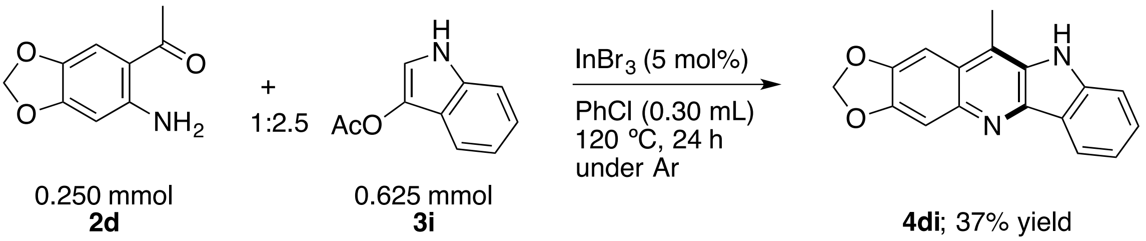

11-Methyl-10*H*-1,3-dioxolo[4,5-*b*]quindoline (4di). $CHCl_3$ instead of EtOAc was used to extract an aqueous phase in a work-up process, and the title compound was isolated by recrystallization from *n*-hexane/EtOAc after column chromatography on silica gel (CH_2Cl_2/MeOH = 100/1). A pale green solid, mp 269–271 ºC (decomp.). ^{1}H NMR (400 MHz, dimethyl sulfoxide-d_6) δ 2.81 (s, 3 H), 6.20 (s, 2 H), 7.23 (ddd, J = 8.0, 4.5, 3.5 Hz, 1 H), 7.49 (s, 1 H), 7.50–7.56 (m, 3 H), 8.24 (d, J = 7.6 Hz, 1 H), 11.21 (bs, 1 H); ^{13}C NMR (100 MHz, dimethyl sulfoxide-d_6) δ 12.8, 98.7, 101.6, 105.0, 111.3, 119.0, 120.7, 121.2, 121.6, 122.8, 128.4, 131.2, 141.4, 142.3, 142.8, 146.8, 147.9. HRMS (FD) Calcd for $C_{17}H_{12}N_2O_2$: M, 276.0899. Found: *m*/*z* 276.0911.

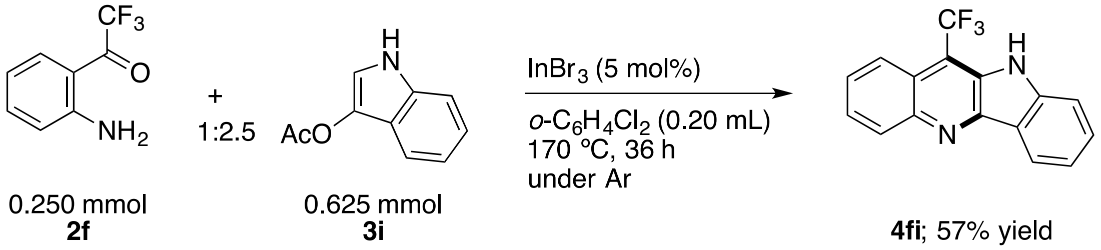

11-Trifluoromethyl-10*H*-quindoline (4fi). The title compound was isolated by column chromatography on silica gel (*n*-hexane/EtOAc = 10/1). A pale green solid, mp 163–164 ºC. ^{1}H NMR (400 MHz, dimethyl sulfoxide-d_6) δ 7.38 (ddd, J = 7.9, 5.0, 2.6 Hz, 1 H), 7.69–7.75 (m, 2 H), 7.76–7.83 (m, 2 H), 8.21 (dq, J = 9.8, 2.3 Hz, 1 H), 8.33–8.37 (m, 1 H), 8.40 (d, J = 7.6 Hz, 1 H), 11.55 (bs, 1 H); ^{13}C NMR (100 MHz, dimethyl sulfoxide-d_6) δ 108.5 (q, J = 31.5 Hz), 112.3, 120.1, 120.7, 121.0 (q, J = 1.9 Hz), 121.5, 122.5 (q, J = 2.4 Hz), 125.0 (q, J = 274.1 Hz), 126.6, 127.4, 128.4 (q, J = 1.9 Hz), 129.9, 131.0, 142.9, 144.9, 147.7; ^{19}F NMR (471 MHz, dimethyl sulfoxide-d_6) δ –53.4. HRMS (FD) Calcd for $C_{16}H_9F_3N_2$: M, 286.0718. Found: *m*/*z* 286.0727.

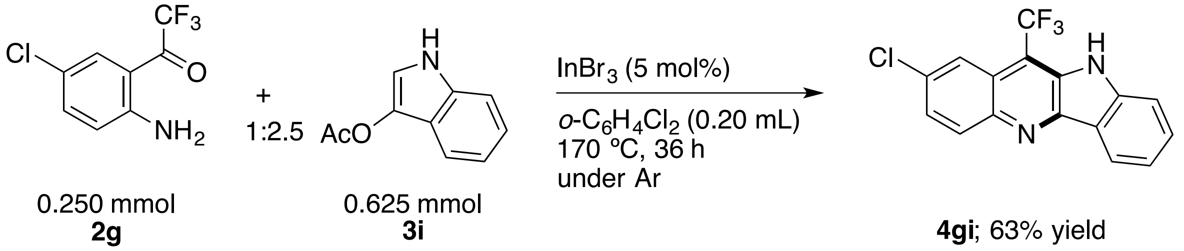

2-Chloro-11-trifluoromethyl-10*H*-quindoline (4gi). The title compound was isolated by column chromatography on silica gel (*n*-hexane/EtOAc = 10/1). A pale green solid, mp 236–238 ºC. ^{1}H NMR (400 MHz, dimethyl sulfoxide-d_6) δ 7.40 (ddd, J = 7.8, 6.4, 1.5 Hz, 1 H), 7.69–7.77 (m, 2 H), 7.81 (dd, J = 9.0, 1.9 Hz, 1 H), 8.08–8.14 (m, 1 H), 8.33–8.41 (m, 2 H), 11.67 (bs, 1 H); ^{13}C NMR (100 MHz, dimethyl sulfoxide-d_6) δ 107.5 (q, J = 32.1 Hz), 112.4, 119.8, 121.0, 121.0 (q, J = 2.9 Hz), 121.5, 121.6 (q, J = 1.4 Hz), 124.7 (q, J = 273.9 Hz), 127.0, 128.8 (q, J = 1.9 Hz), 131.3, 131.92, 131.94, 141.1, 145.1, 148.1; ^{19}F NMR (471 MHz, dimethyl sulfoxide-d_6) δ –53.7. HRMS (FD) Calcd for $C_{16}H_8ClF_3N_2$: M, 320.0328. Found: *m*/*z* 320.0343.

II. N-Methylation of Indolo[3,2-*b*]quinolines with MeOTf: A General Procedure for Table 6

Compounds **10di**, **10fi** and **10gi** were synthesized based on the modified literature procedure [2], as follows: A flame-dried 20 mL Schlenk tube was charged with **4** (0.100 mmol) and solvent CH_2Cl_2 (1.2 mL) or solvent toluene (0.60 mL). The resulting solution was degassed by three freeze-pump-thaw cycles, and the tube was then filled with argon. To this solution was added MeOTf (31.2 mg, 0.190 mmol) that had been distilled by Kugelrohr at 90 ºC/500 Pa prior to use, and the mixture was then stirred at 30 or 50 ºC for 24 h. The resulting mixture including a solid product was filtered, and the solid was washed with Et_2O (5 mL). The filtrate was concentrated, and the residue was filtered and then washed with Et_2O (5 mL). This concentration–filtration–washing sequence was repeated once again, and the combined solid was dried in vacuo to give analytically pure product **10**. Compounds **10** synthesized here were fully characterized by ^{1}H, ^{13}C and ^{19}F NMR spectroscopy and HRMS.

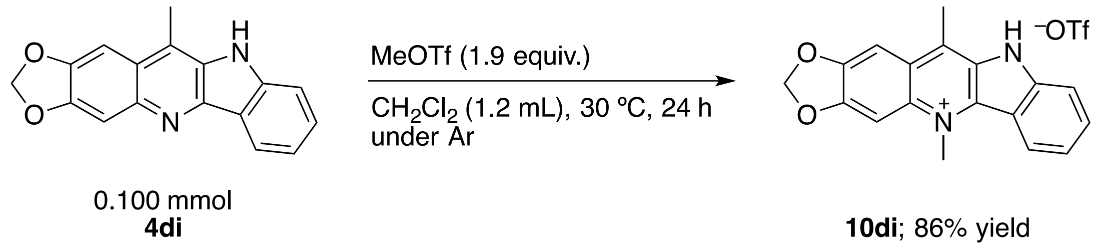

5,11-Dimethyl-10*H*-1,3-dioxolo[4,5-*b*]quindolinium 1,1,1-Trifluoromethanesulfonate (10di). A yellow solid, mp 335–337 ºC (decomp.). ^{1}H NMR (500 MHz, dimethyl sulfoxide-d_6) δ 3.08 (s, 3 H), 4.86 (s, 3 H), 6.44 (s, 2 H), 7.46 (ddd, *J* = 8.5, 6.9, 1.0 Hz, 1 H), 7.78 (ddd, *J* = 8.4, 1.6, 0.9 Hz, 1 H), 7.81–7.86 (m, 1 H), 7.94 (d, *J* = 1.0 Hz, 1 H), 8.21 (d, *J* = 1.0 Hz, 1 H), 8.68 (d, *J* = 8.4 Hz, 1 H), 12.59 (bs, 1 H); ^{13}C NMR (100 MHz, dimethyl sulfoxide-d_6) δ 14.5, 40.4, 95.7, 100.6, 103.9, 112.7, 114.1, 120.7 (q, *J* = 322.4 Hz), 121.0, 123.0, 124.9, 131.6, 131.9, 133.1, 134.3, 134.5, 143.4, 148.2, 153.3; ^{19}F NMR (376 MHz, dimethyl sulfoxide-d_6) δ –77.3. HRMS (FD) Calcd for $C_{18}H_{15}N_2O_2$: M^+, 291.1128. Found: *m*/*z* 291.1124.

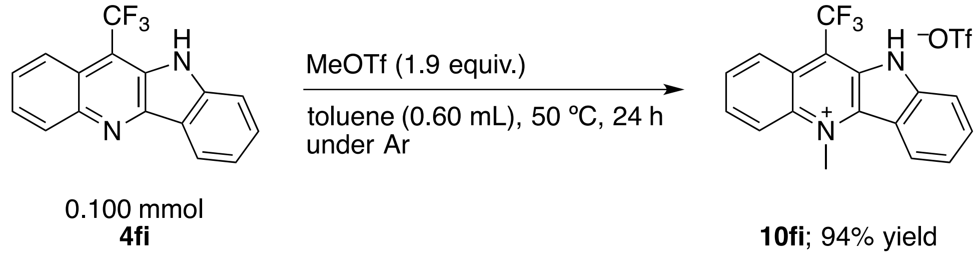

11-Trifluoromethyl-5-methyl-10*H*-quindolinium 1,1,1-Trifluoromethanesulfonate (10fi). Spectral and analytical data of compound **10fi** are collected in section 3.4. of the article.

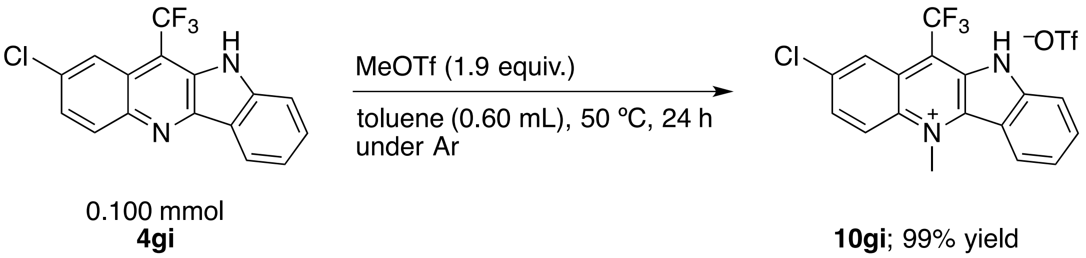

2-Chloro-11-trifluoromethyl-5-methyl-10*H*-quindolinium 1,1,1-Trifluoromethanesulfonate (10gi). An orange solid, mp 321–322 ºC. ^{1}H NMR (500 MHz, dimethyl sulfoxide-d_6) δ 5.13 (s, 3 H), 7.63 (ddd, *J* = 8.6, 6.9, 0.9 Hz, 1 H), 7.96 (d, *J* =

8.3 Hz, 1 H), 8.09 (ddd, J = 8.7, 6.7, 1.1 Hz, 1 H), 8.32 (dd, J = 9.5, 2.0 Hz, 1 H), 8.40–8.48 (m, 1 H), 8.89 (d, J = 8.6 Hz, 1 H), 9.02 (d, J = 9.7 Hz, 1 H), 12.81 (s, 1 H); ^{13}C NMR (125 MHz, dimethyl sulfoxide-d_6) δ 42.1, 113.5, 113.9, 115.5 (q, J = 33.0 Hz), 120.6 (q, J = 322.3 Hz), 121.8, 122.3, 122.4 (q, J = 3.4 Hz), 122.8, 122.9 (q, J = 275.9 Hz), 127.3, 131.7 (q, J = 1.8 Hz), 131.9, 133.8, 134.3, 136.4, 143.2, 147.6; ^{19}F NMR (376 MHz, dimethyl sulfoxide-d_6) δ –77.3, –53.7. HRMS (FD) Calcd for $C_{17}H_{11}ClF_3N_2$: M^+, 335.0557. Found: m/z 335.0546.

III. Control Experiments for Mechanistic Studies (Scheme 3)

III-1. Indium-Catalyzed S_NAr Amination of 3-Methoxybenzothiophene with 1-(2-Aminophenyl)-2-methyl-1-propanone: An Experimental Procedure for Eq. 1 in Scheme 3

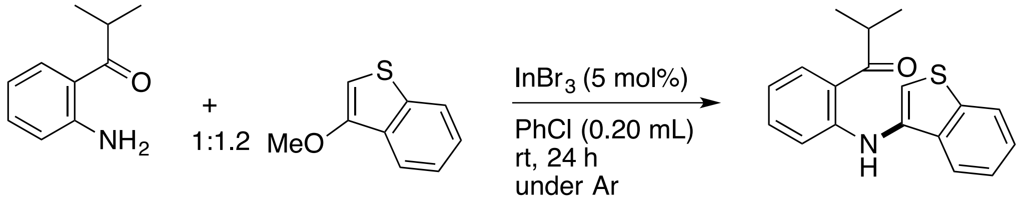

0.250 mmol
2e

0.300 mmol
3a

6ea; 53% yield

The reaction of **2e** with **3a** for the synthesis of **6ea** was carried out in a similar way to the procedure described in section "**I. Indium-Catalyzed Annulation of *o*-Acylanilines with Alkoxyheteroarenes: A General Procedure for Tables 3–5**". Compound **6ea** was fully characterized by ^{1}H and ^{13}C NMR spectroscopy and HRMS.

1-[2-(Benzo[*b*]thien-3-ylamino)phenyl]-2-methyl-1-propanone (6ea). The title compound was isolated by column chromatography on silica gel (*n*-hexane/EtOAc = 40/1). A yellow solid, mp 96–97 ºC. ^{1}H NMR (500 MHz, $CDCl_3$) δ 1.29 (d, J = 6.9 Hz, 6 H), 3.72 (sept, J = 6.8 Hz, 1 H), 6.77 (dd, J = 8.0, 7.2 Hz, 1 H), 7.19 (d, J = 8.6 Hz, 1 H), 7.19 (s, 1 H), 7.31–7.35 (m, 1 H), 7.37–7.43 (m, 2 H), 7.75–7.81 (m, 1 H), 7.83–7.88 (m, 1 H), 7.94 (dd, J = 8.0, 0.9 Hz, 1 H), 10.97 (bs, 1 H); ^{13}C NMR (100 MHz, $CDCl_3$) δ 19.8, 35.6, 113.3, 114.9, 116.6, 117.4, 121.3, 123.0, 124.2, 125.0, 131.5, 133.0, 134.6, 135.2, 138.5, 149.1, 207.9. HRMS (FD) Calcd for $C_{18}H_{17}NOS$: M, 295.1031. Found: m/z 295.1044.

III-2. Indium-Catalyzed Intramolecular Cyclization of 6ea: An Experimental Procedure for Eq. 2 in Scheme 3

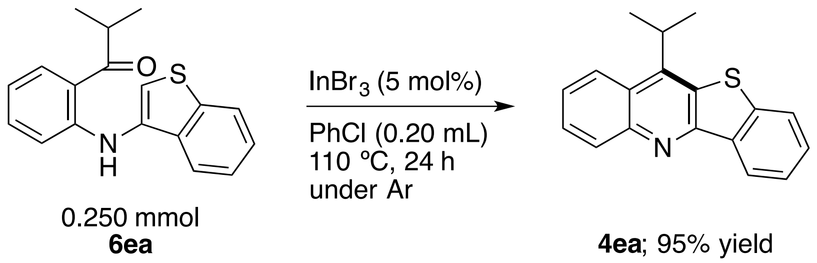

$InBr_3$ (4.43 mg, 12.5 μmol) was placed in a 20 mL Schlenk tube, which was heated at 80 °C in vacuo for 15 min. The tube was cooled down to room temperature and filled with argon. PhCl (0.20 mL) was added to the tube, and the mixture was then stirred at room temperature for 3 min. To this was added **6ea** (73.8 mg, 0.250 mmol), and the mixture was stirred at 110 ºC for 24 h. A saturated $NaHCO_3$ aqueous solution (0.5 mL) was added at room temperature, and the resulting mixture was stirred for 20 min. The aqueous phase was extracted with EtOAc (5 mL × 3). The combined organic layer was washed with brine (1 mL) and then dried over anhydrous sodium sulfate (Na_2SO_4). Filtration and evaporation of the solvent followed by column chromatography on silica gel (*n*-hexane/EtOAc = 40/1) gave 11-(1-methylethyl)[1]benzothieno[3,2-*b*]quinoline (**4ea**) in 95% yield (65.9 mg) as a pale pink solid. Compound **4ea** has already appeared in this Supplementary Materials, and its spectral and analytical data are thus collected there (*vide supra*).

IV. ^{1}H, ^{13}C and/or ^{19}F NMR Spectra

NMR spectra of substrates and products are collected in the following pages. Only ^{1}H NMR spectra are provided in the case of compounds for which ^{1}H NMR, ^{13}C NMR, and HRMS or elemental analysis data have been already reported in the literature. Both of ^{1}H and ^{13}C NMR spectra are provided in the case of compounds that have not been reported in the literature. In the case of compounds with fluorine atoms that have not been reported in the literature, ^{19}F NMR spectra are also provided along with ^{1}H and ^{13}C NMR spectra.

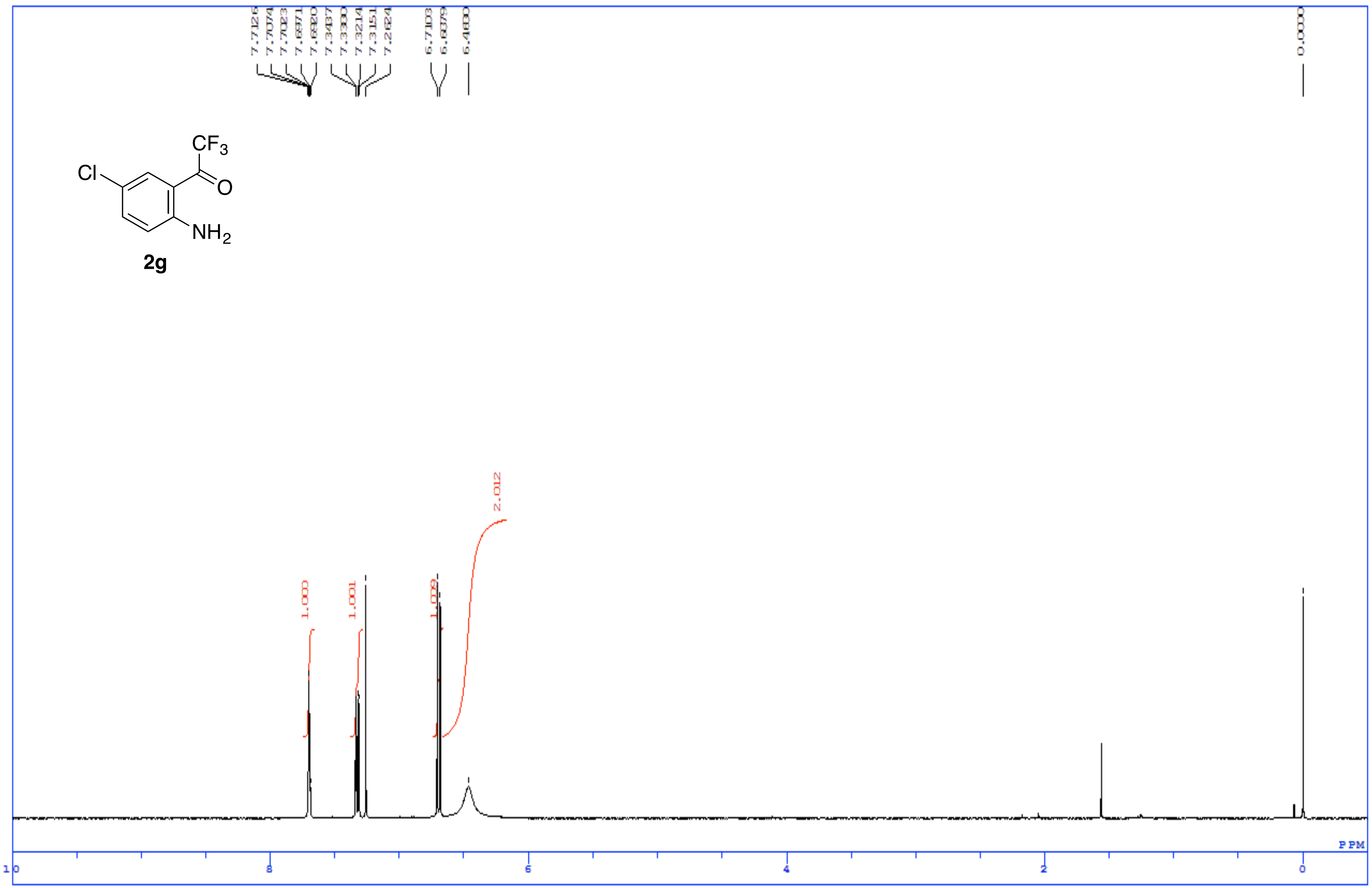

Figure S1. ^{1}H NMR spectrum of compound **2g** in $CDCl_3$.

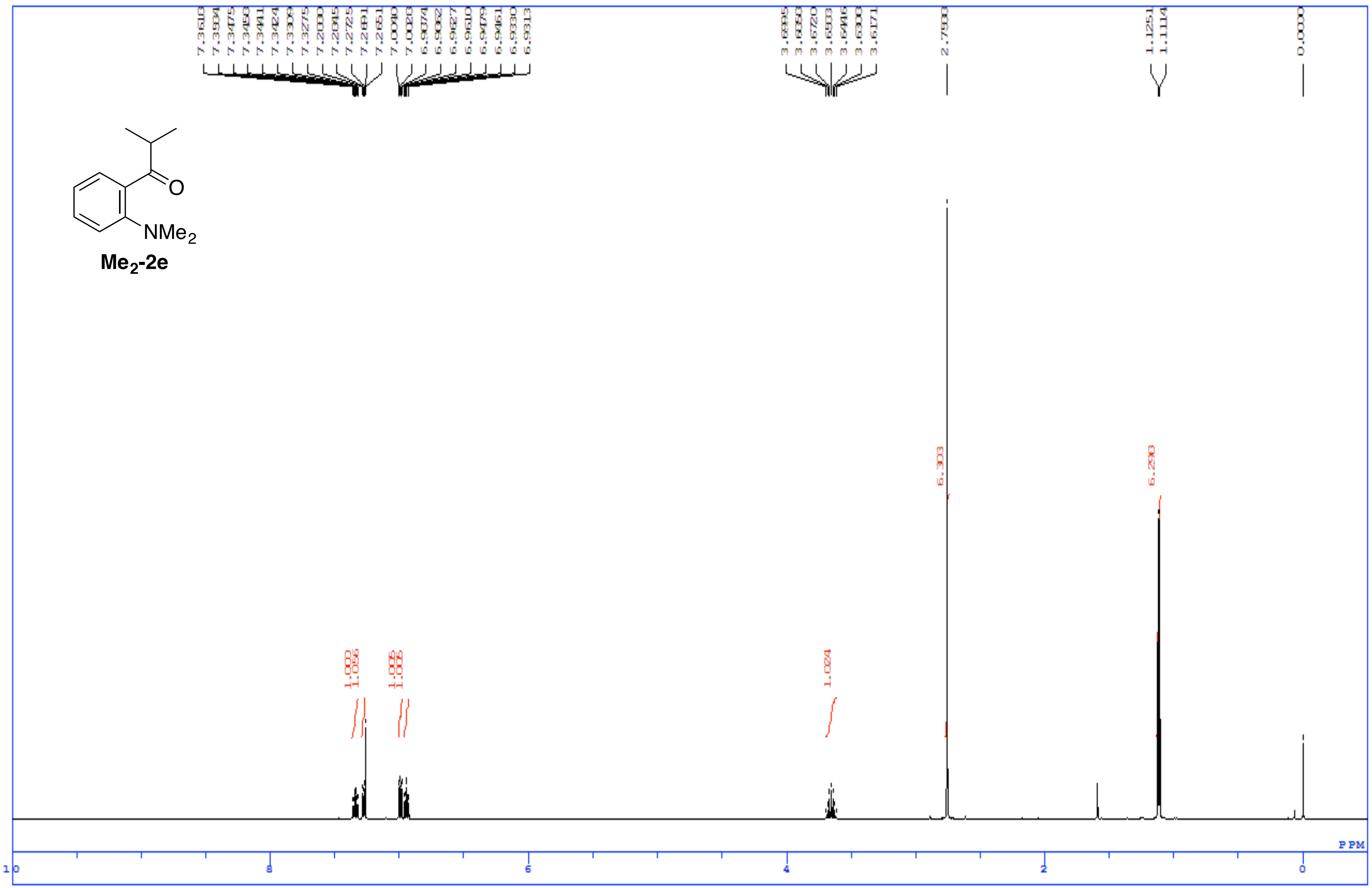

Figure S2. ^{1}H NMR spectrum of compound **Me₂-2e** in $CDCl_3$.

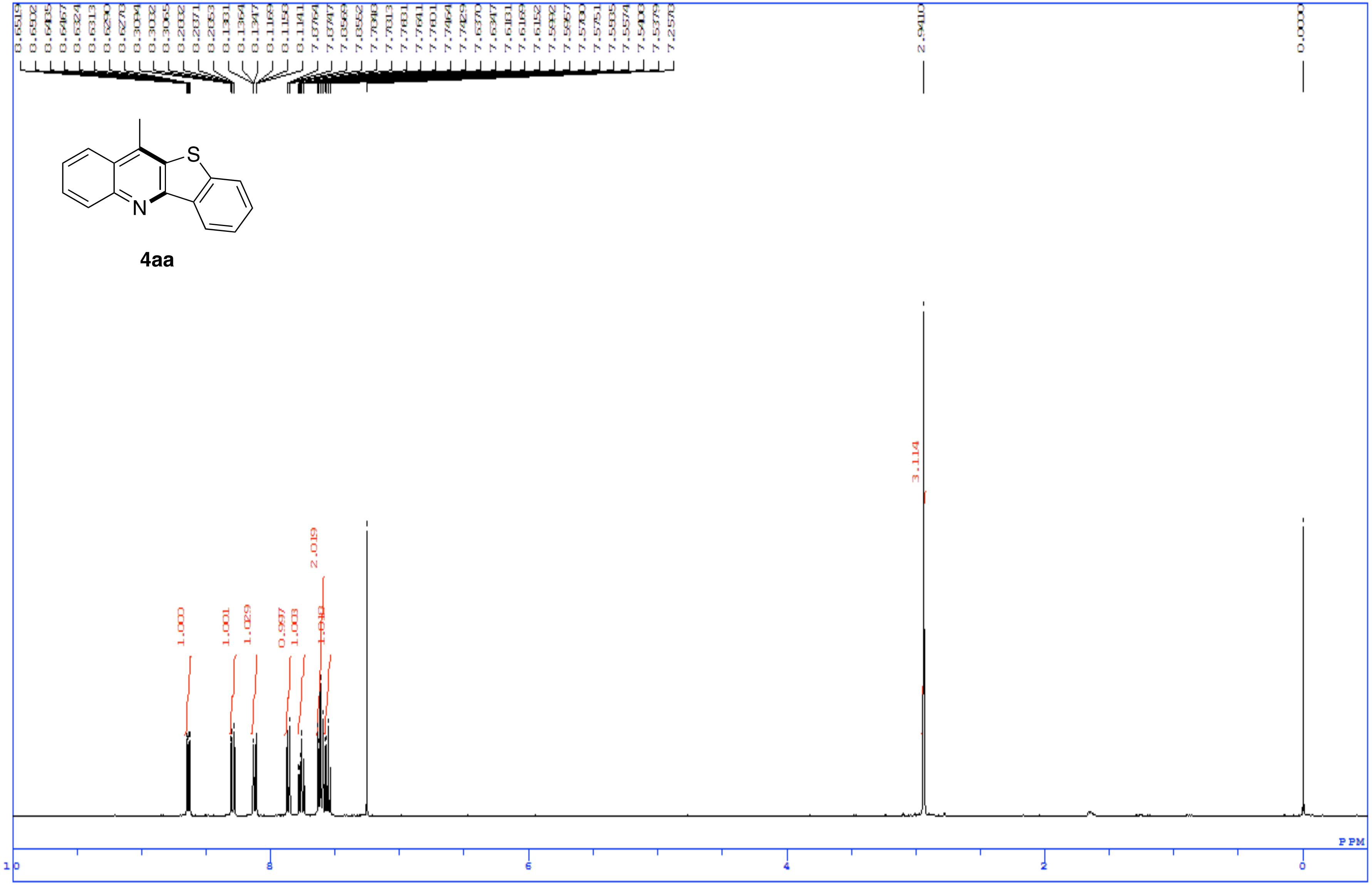

Figure S3. ^{1}H NMR spectrum of compound **4aa** in $CDCl_3$.

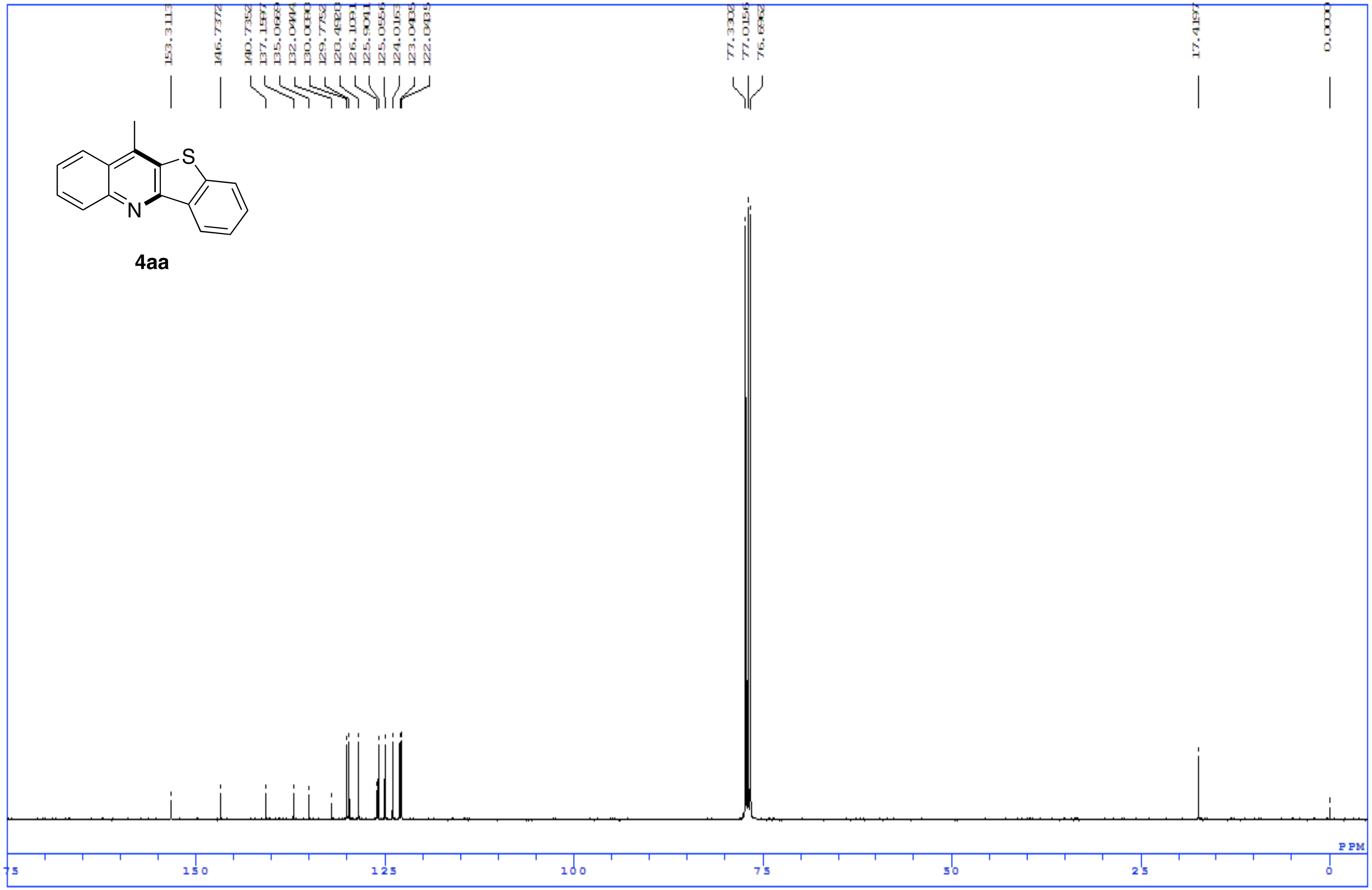

Figure S4. ^{13}C NMR spectrum of compound **4aa** in $CDCl_3$.

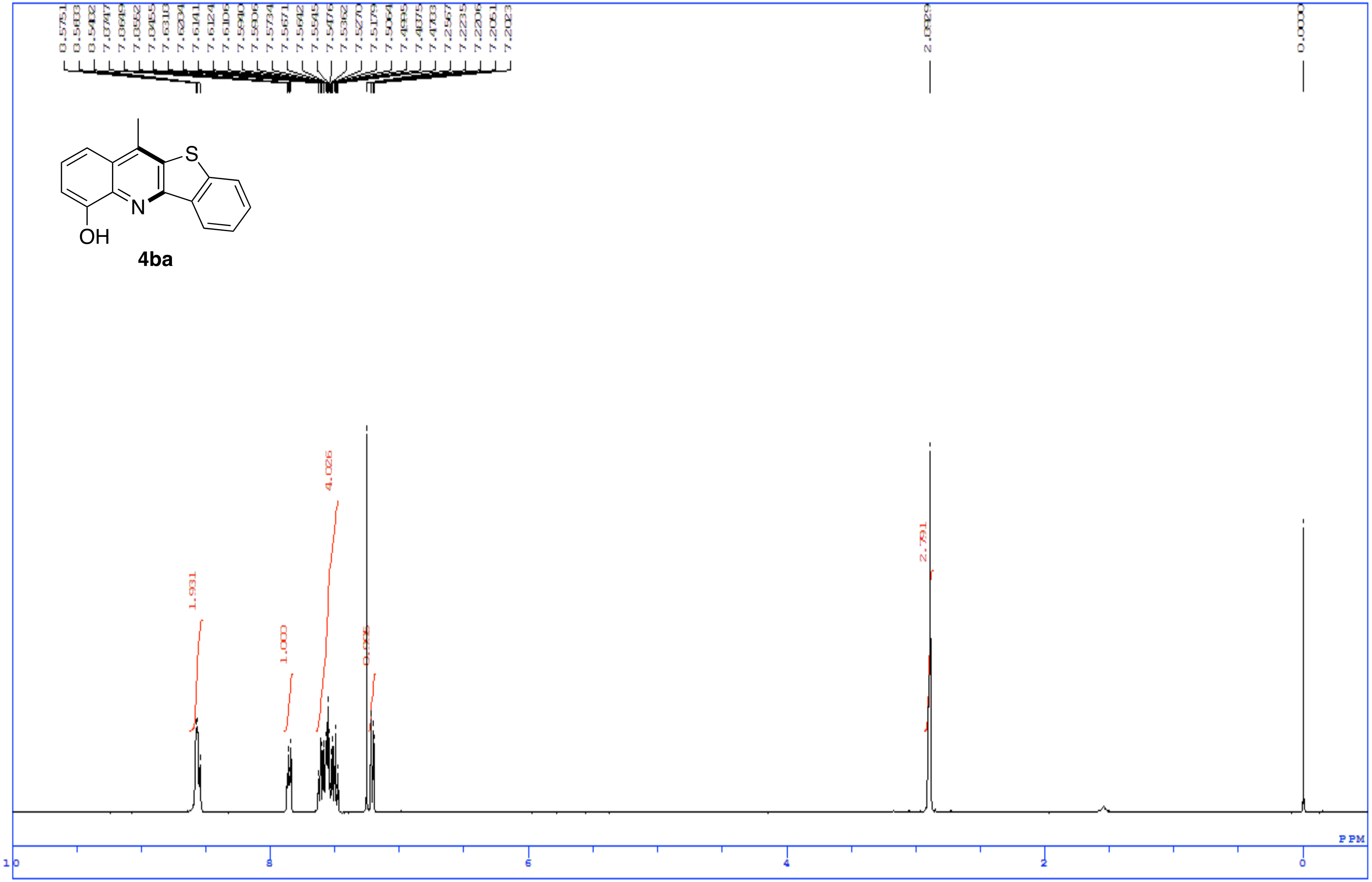

Figure S5. ^{1}H NMR spectrum of compound **4ba** in $CDCl_3$.

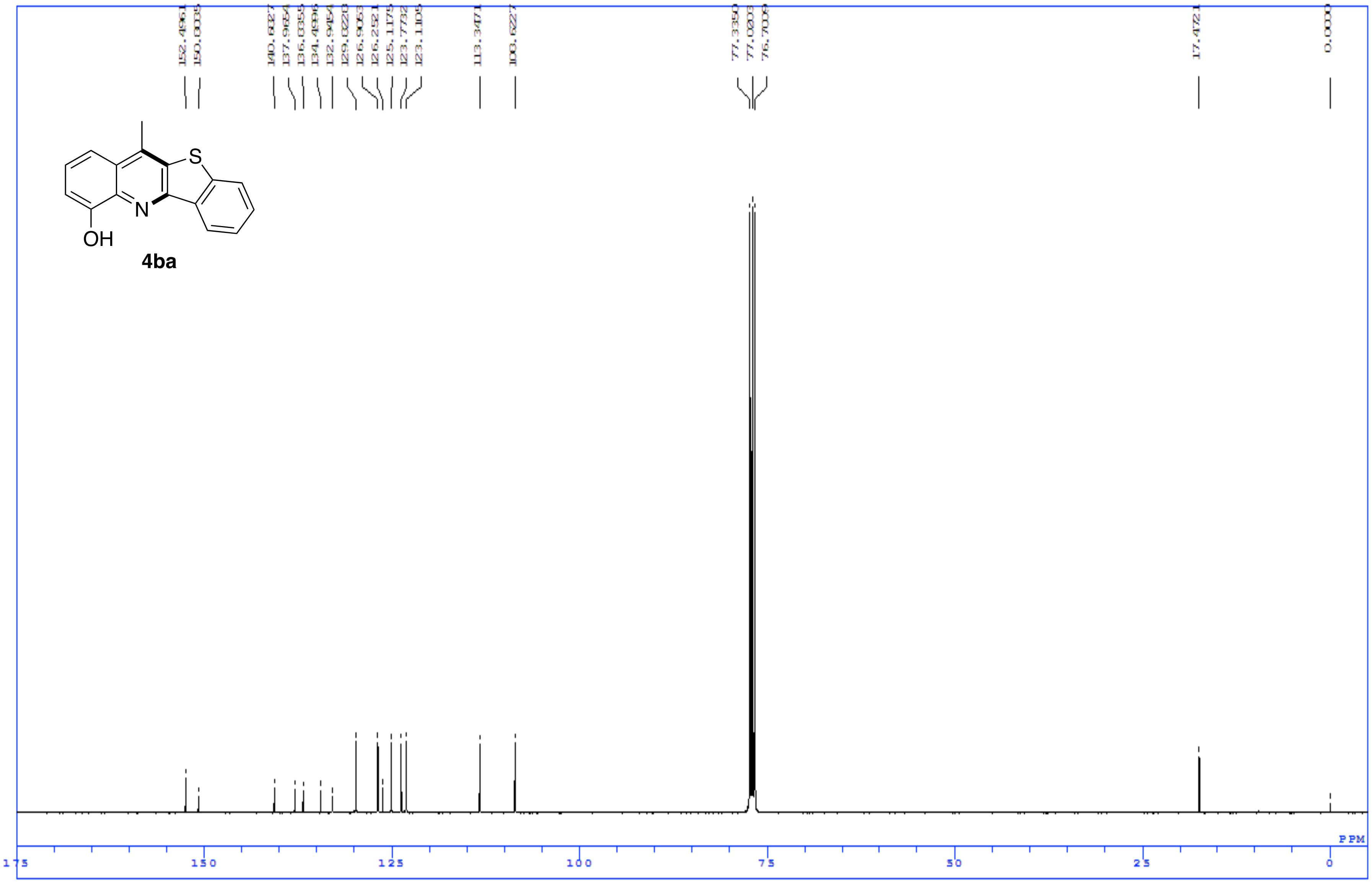

Figure S6. ^{13}C NMR spectrum of compound **4ba** in $CDCl_3$.

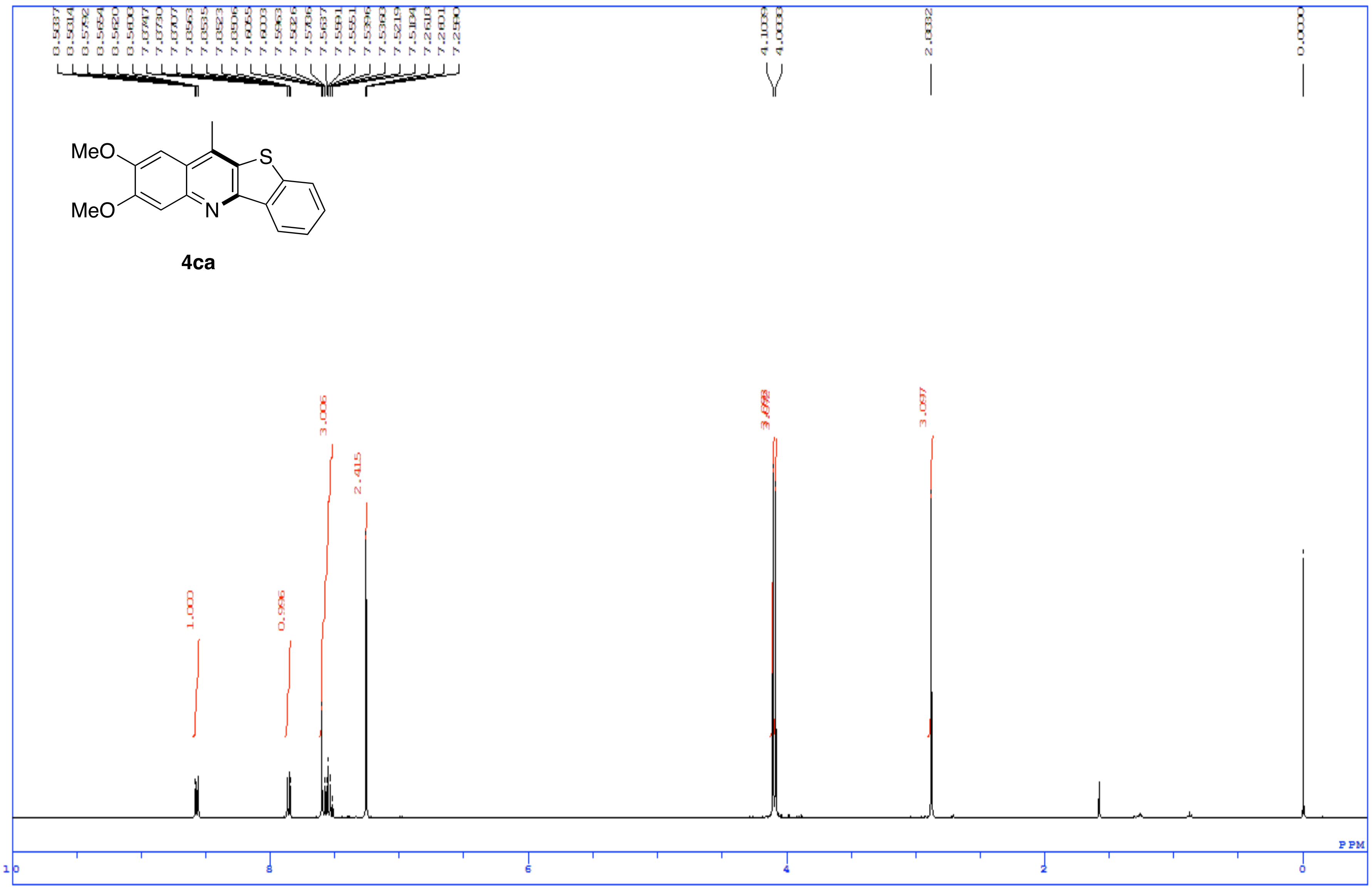

Figure S7. ^{1}H NMR spectrum of compound **4ca** in $CDCl_3$.

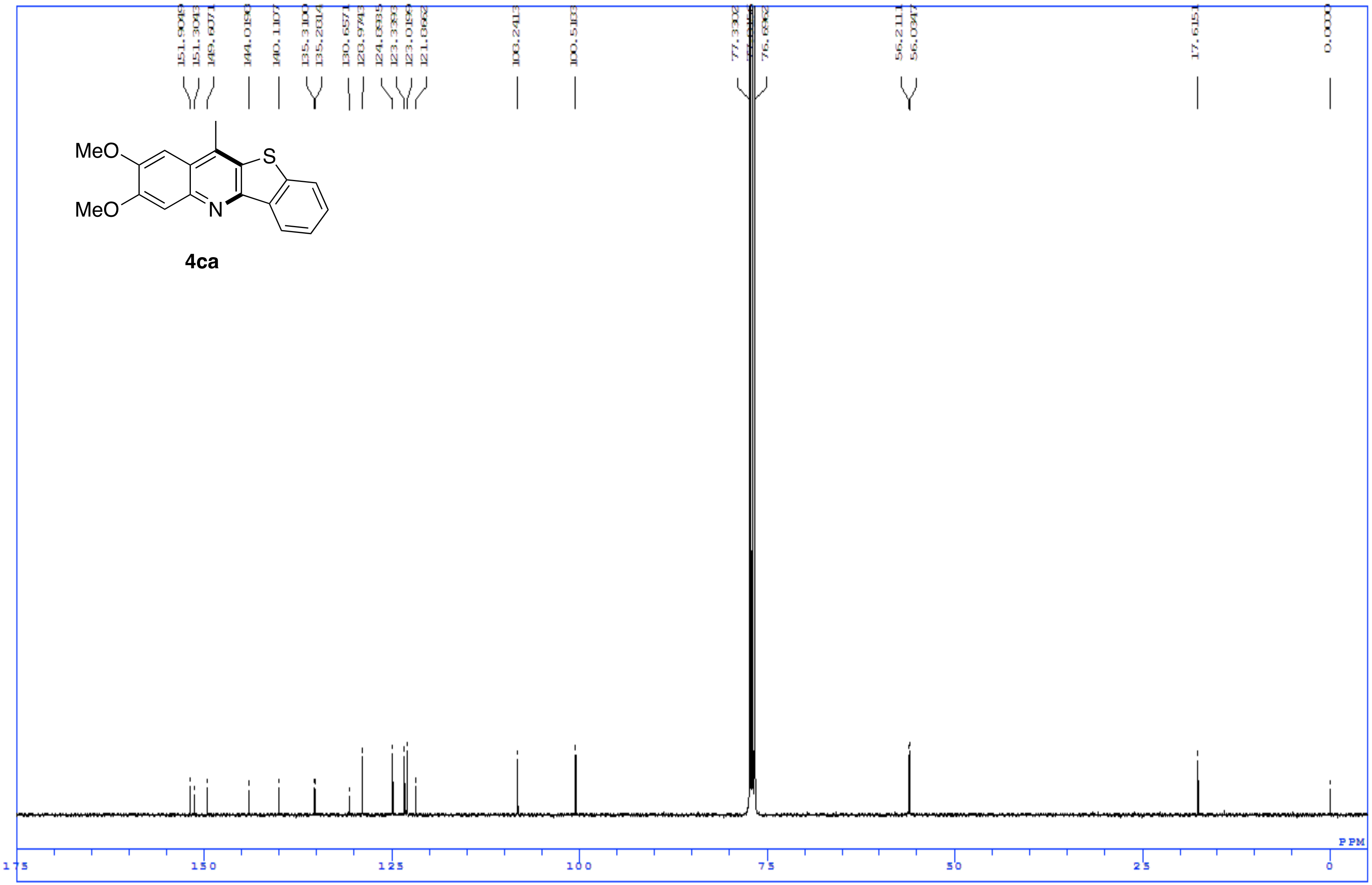

Figure S8. ^{13}C NMR spectrum of compound **4ca** in $CDCl_3$.

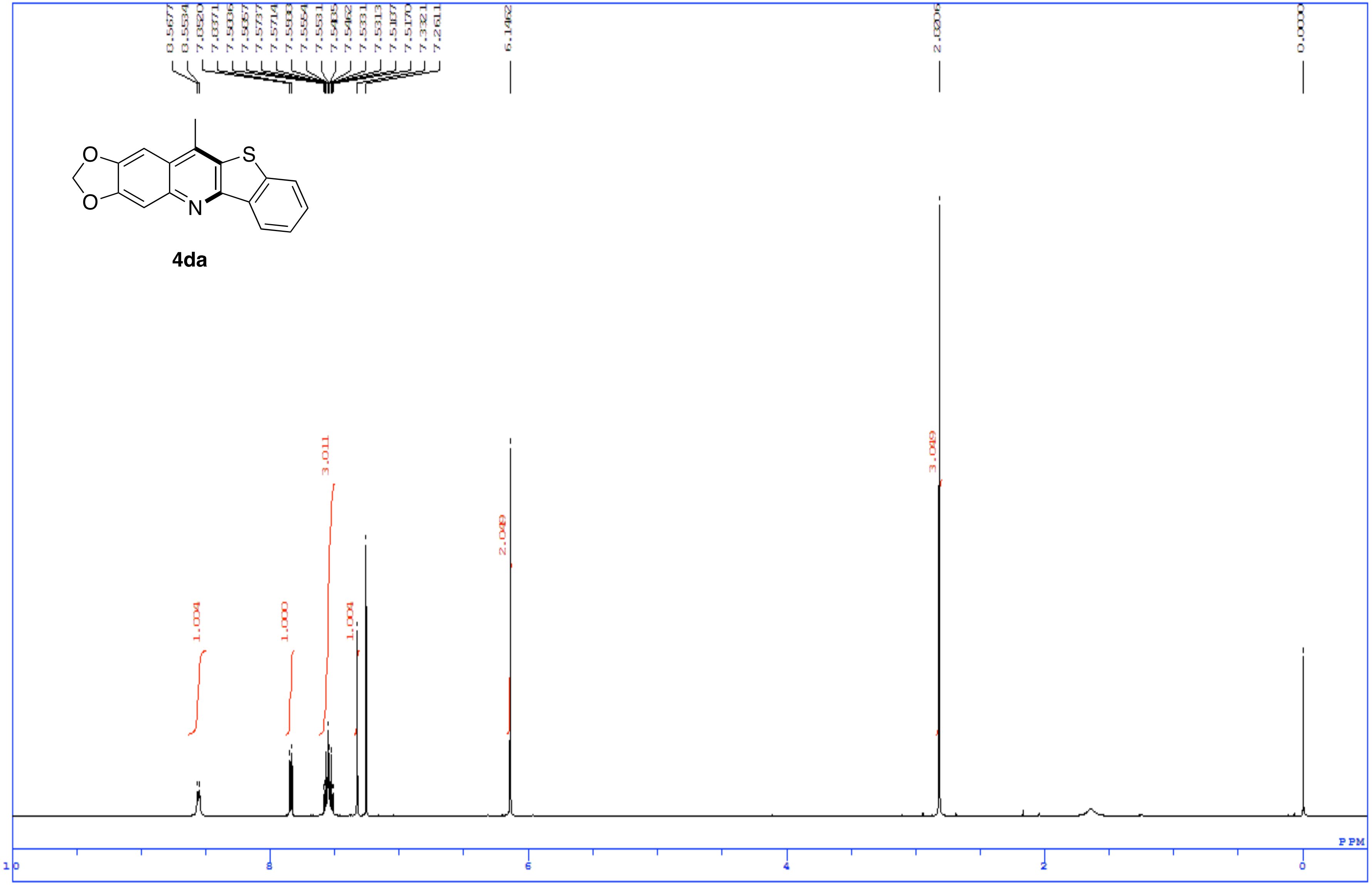

Figure S9. ^{1}H NMR spectrum of compound **4da** in $CDCl_3$.

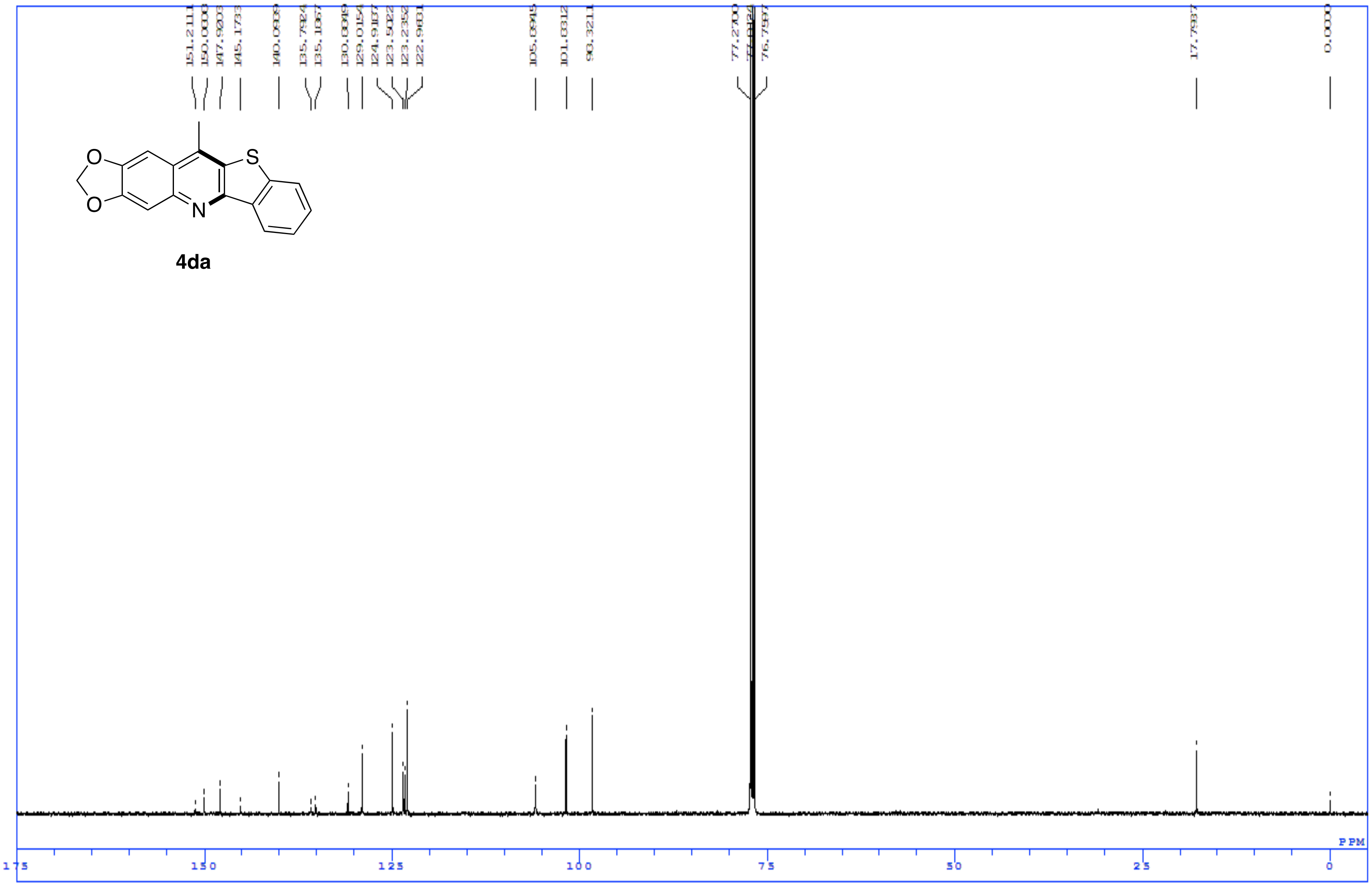

Figure S10. ^{13}C NMR spectrum of compound **4da** in $CDCl_3$.

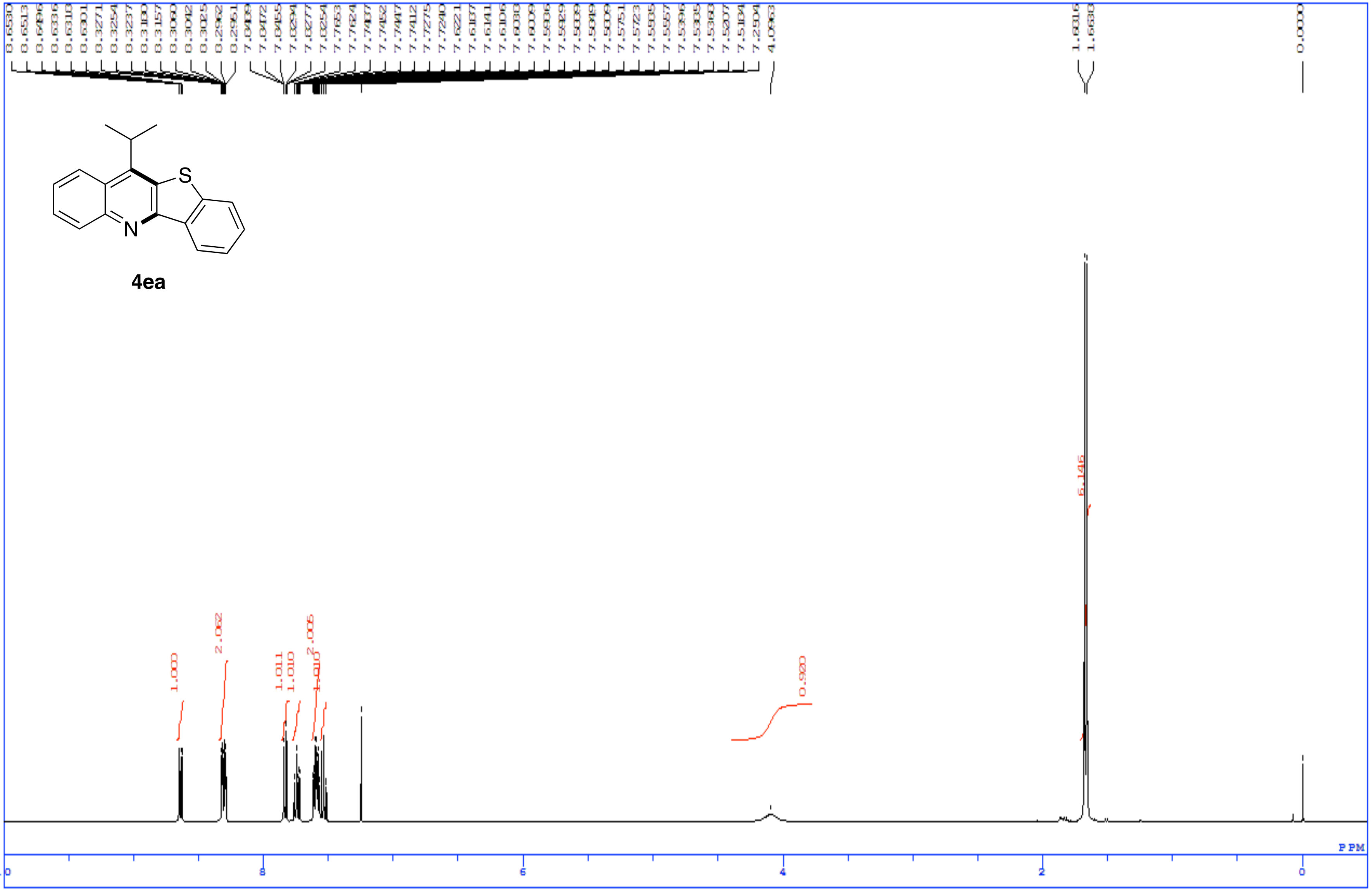

Figure S11. ^{1}H NMR spectrum of compound **4ea** in $CDCl_3$.

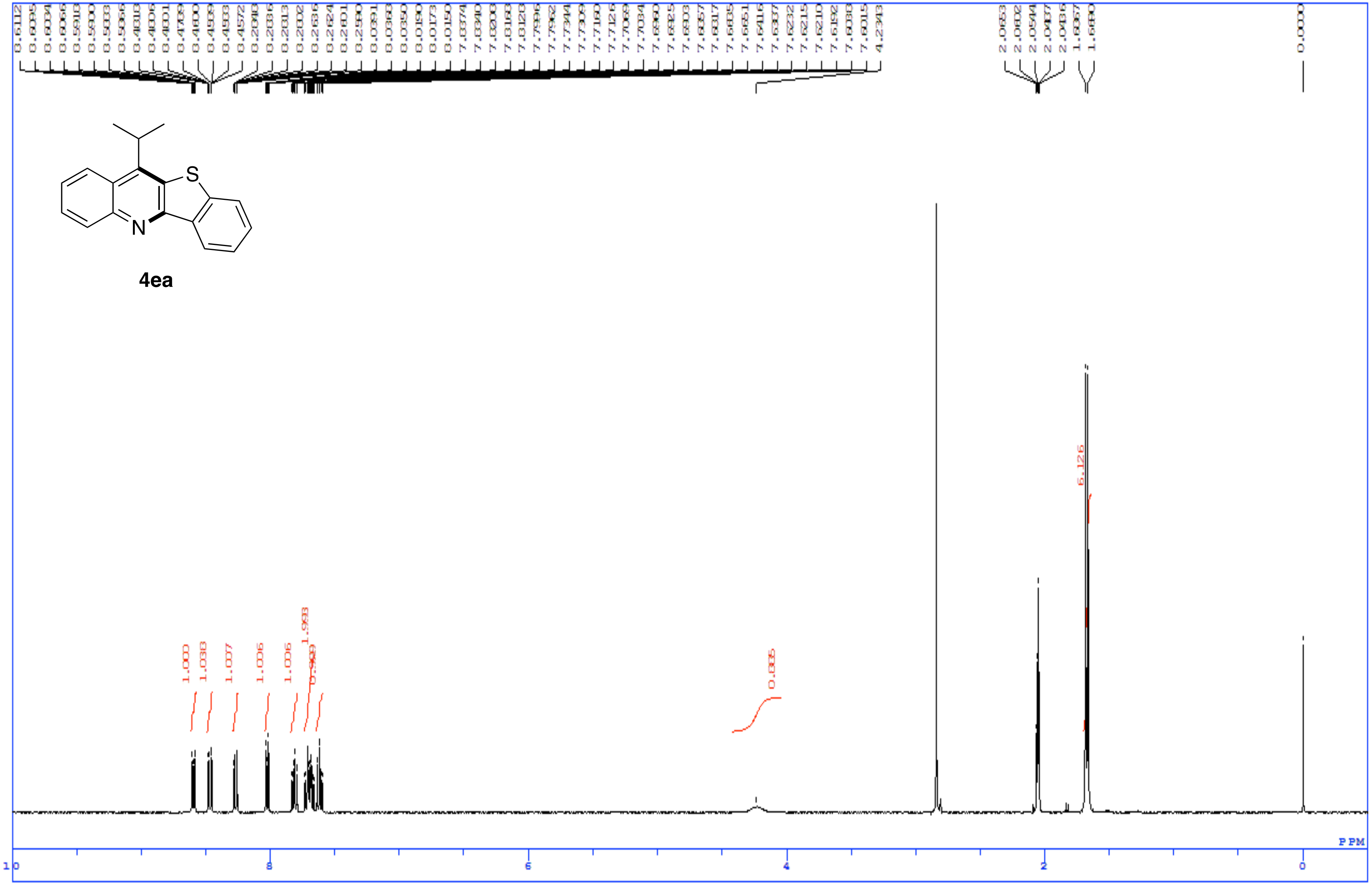

Figure S12. ^{1}H NMR spectrum of compound **4ea** in acetone-d_6.

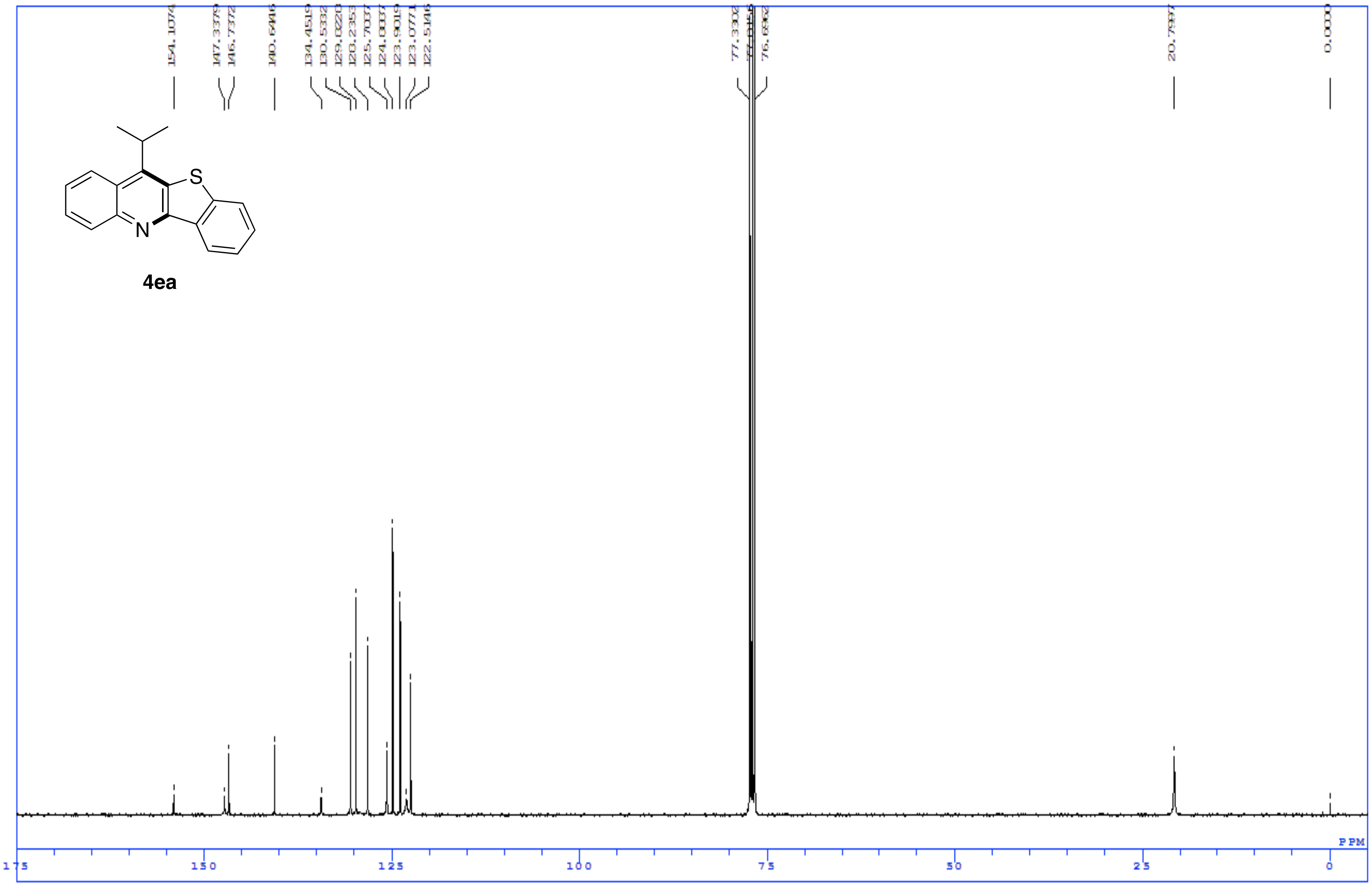

Figure S13. ^{13}C NMR spectrum of compound **4ea** in $CDCl_3$.

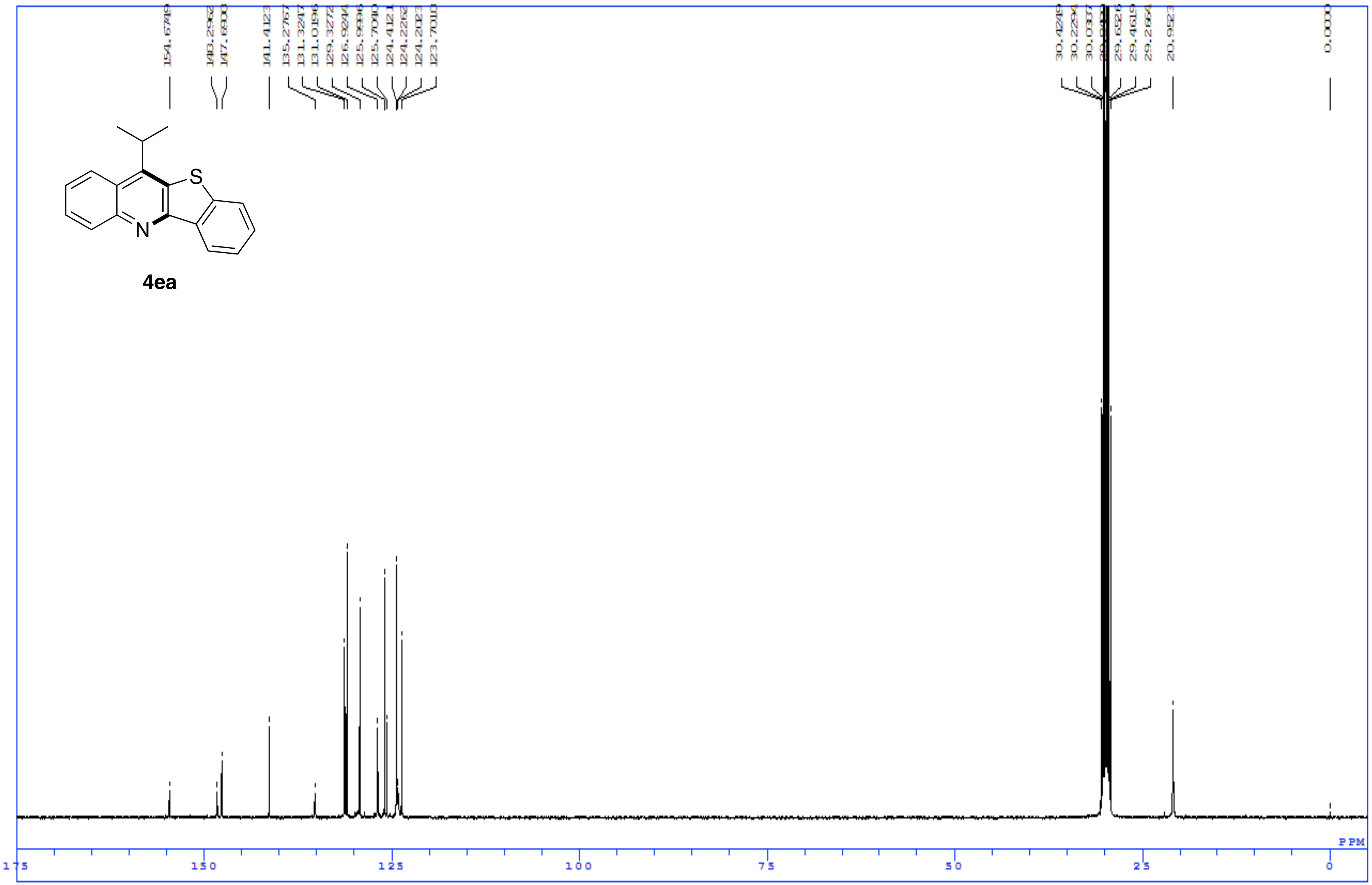

Figure S14. ^{13}C NMR spectrum of compound **4ea** in acetone-d_6.

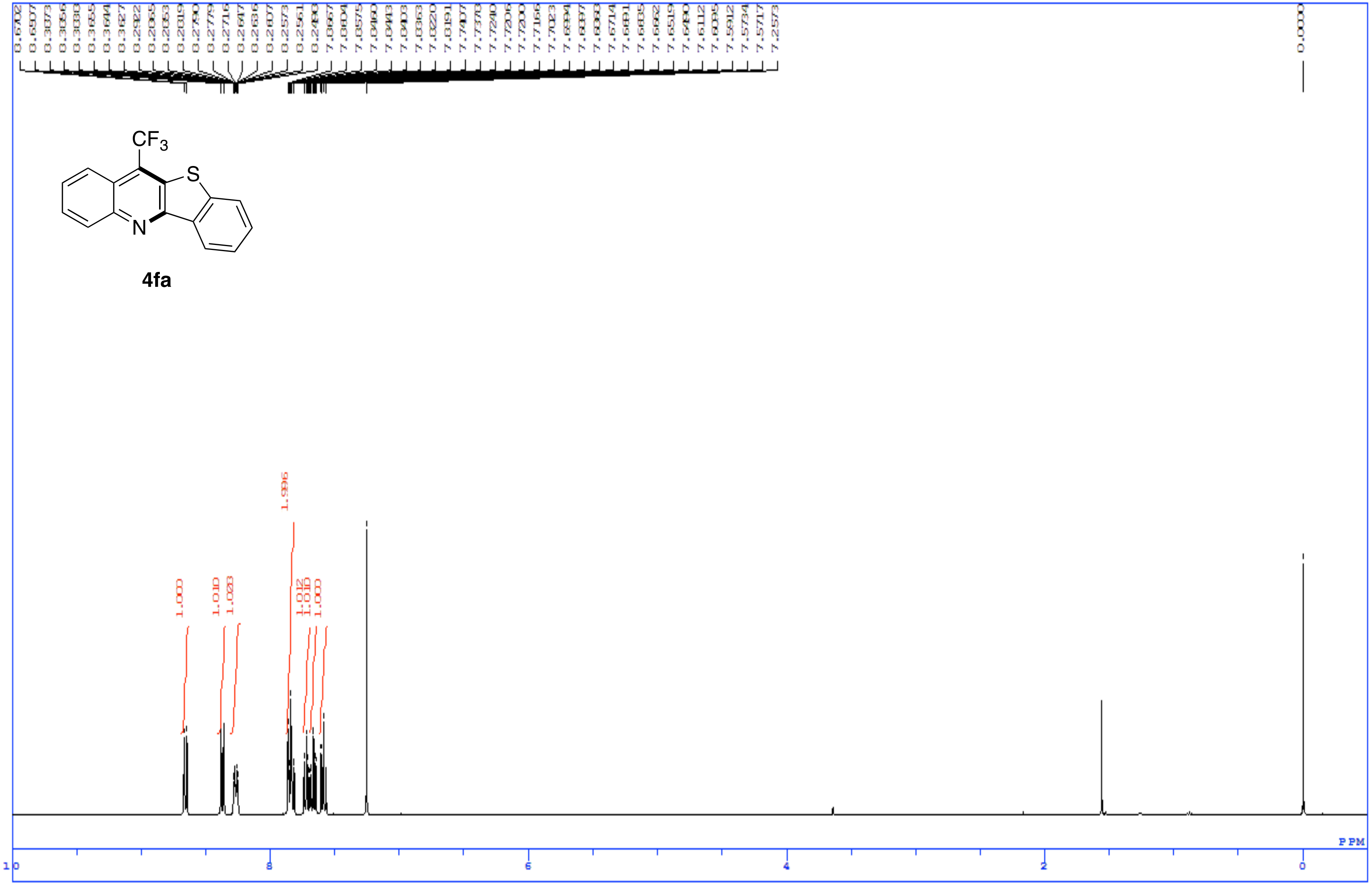

Figure S15. ^{1}H NMR spectrum of compound **4fa** in $CDCl_3$.

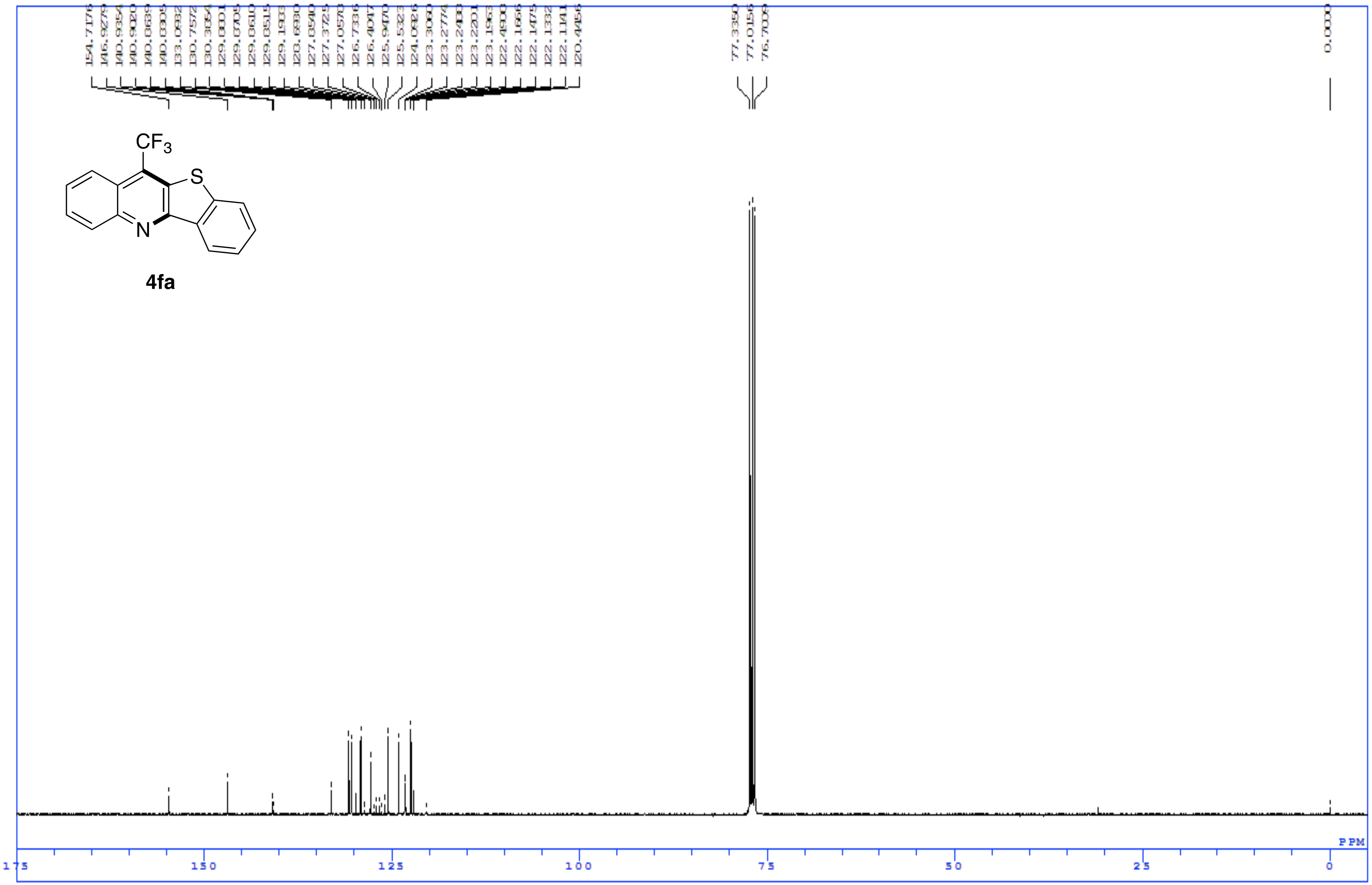

Figure S16. ^{13}C NMR spectrum of compound **4fa** in $CDCl_3$.

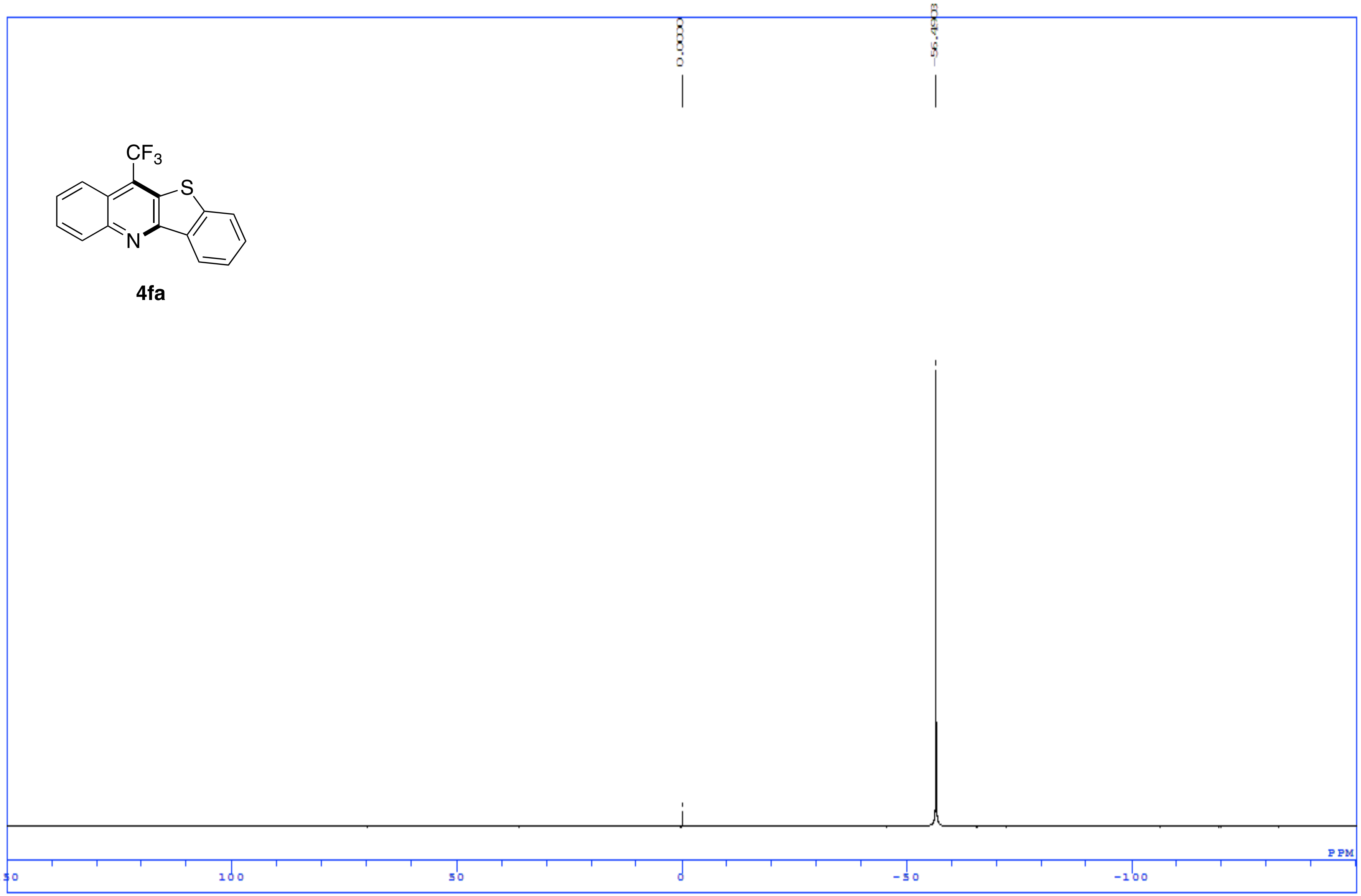

Figure S17. ^{19}F NMR spectrum of compound **4fa** in $CDCl_3$.

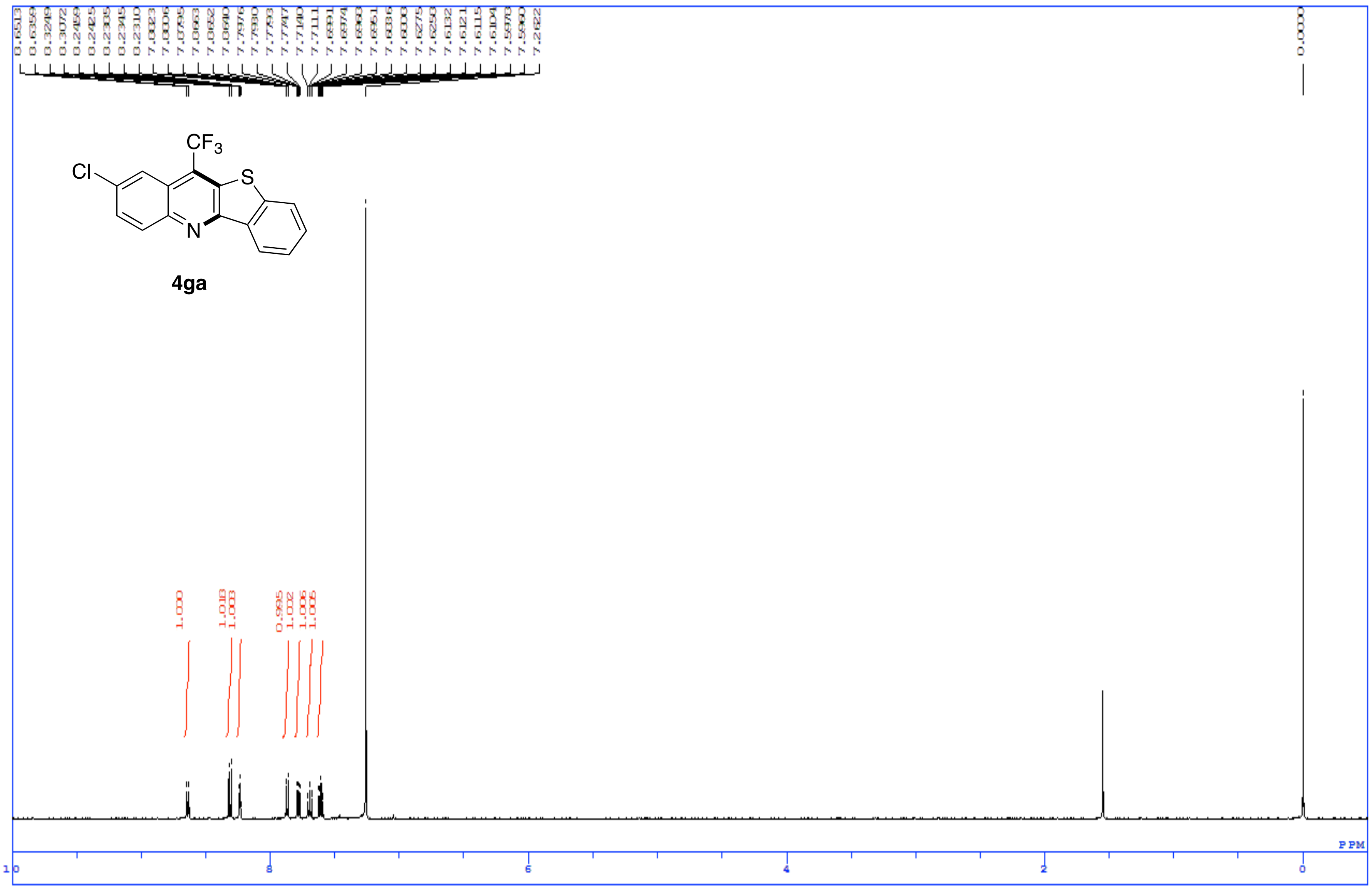

Figure S18. ^{1}H NMR spectrum of compound **4ga** in $CDCl_3$.

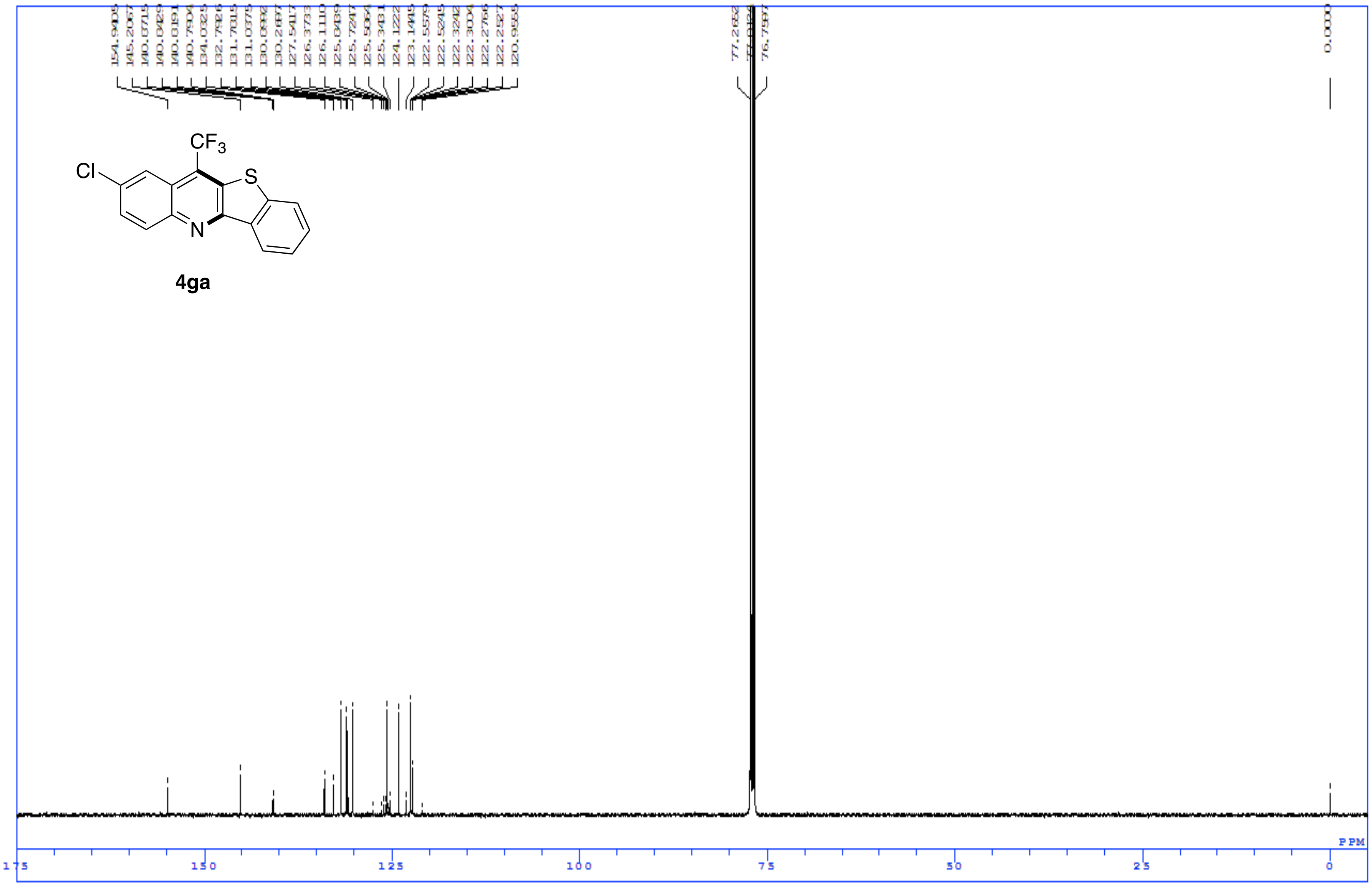

Figure S19. ^{13}C NMR spectrum of compound **4ga** in $CDCl_3$.

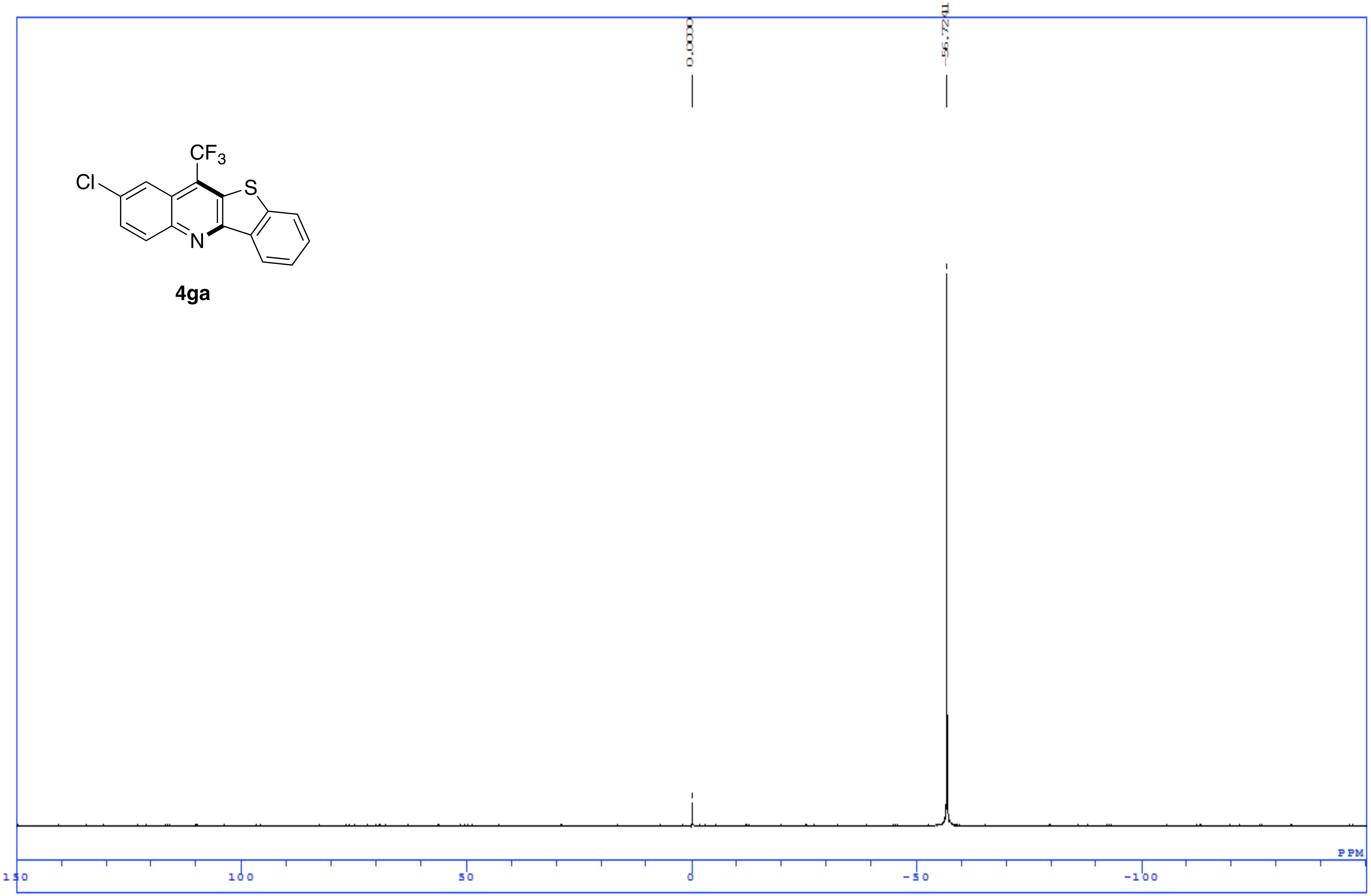

Figure S20. ^{19}F NMR spectrum of compound **4ga** in $CDCl_3$.

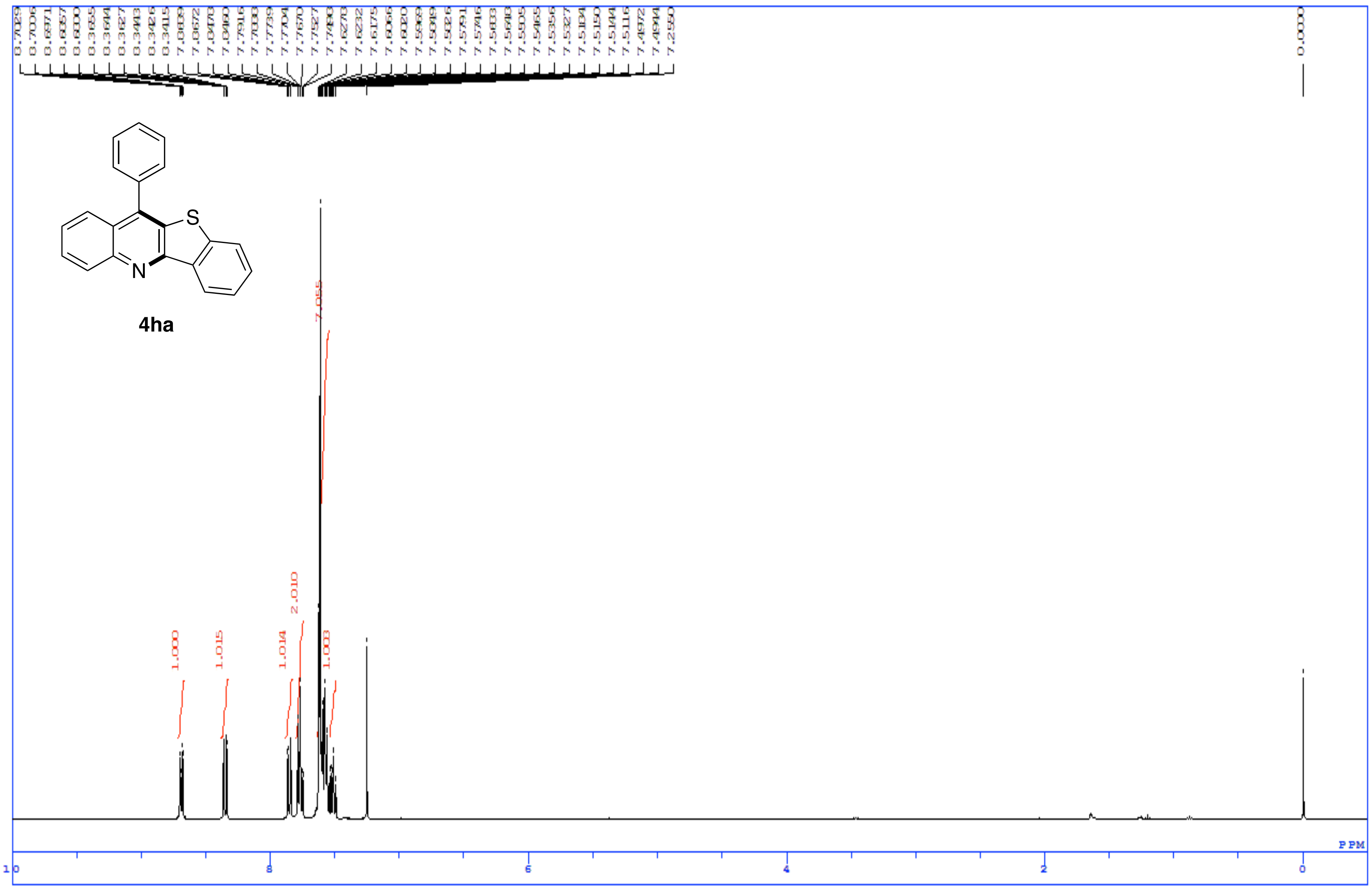

Figure S21. ^{1}H NMR spectrum of compound **4ha** in $CDCl_3$.

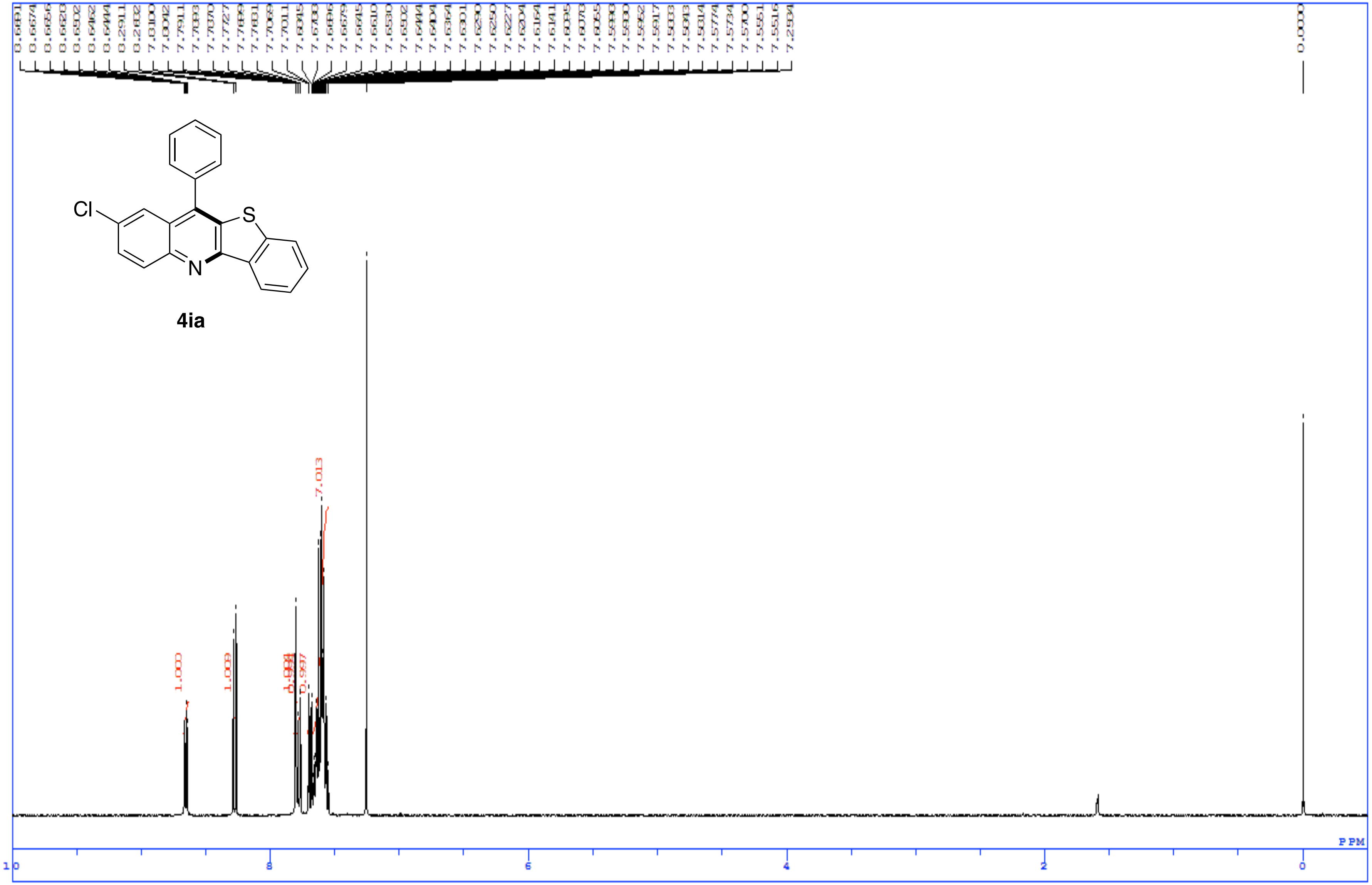

Figure S22. ^{1}H NMR spectrum of compound **4ia** in $CDCl_3$.

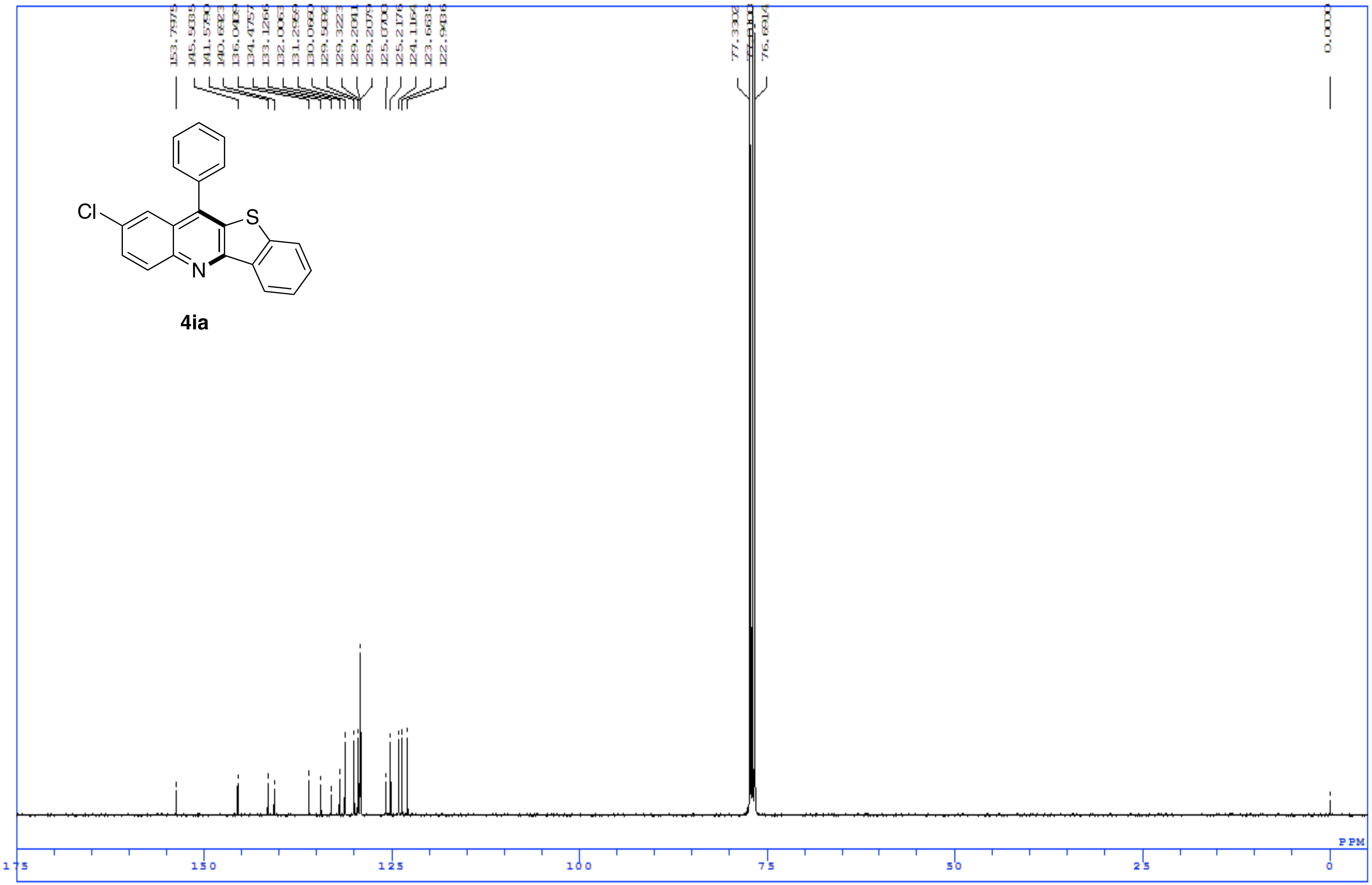

Figure S23. ^{13}C NMR spectrum of compound **4ia** in $CDCl_3$.

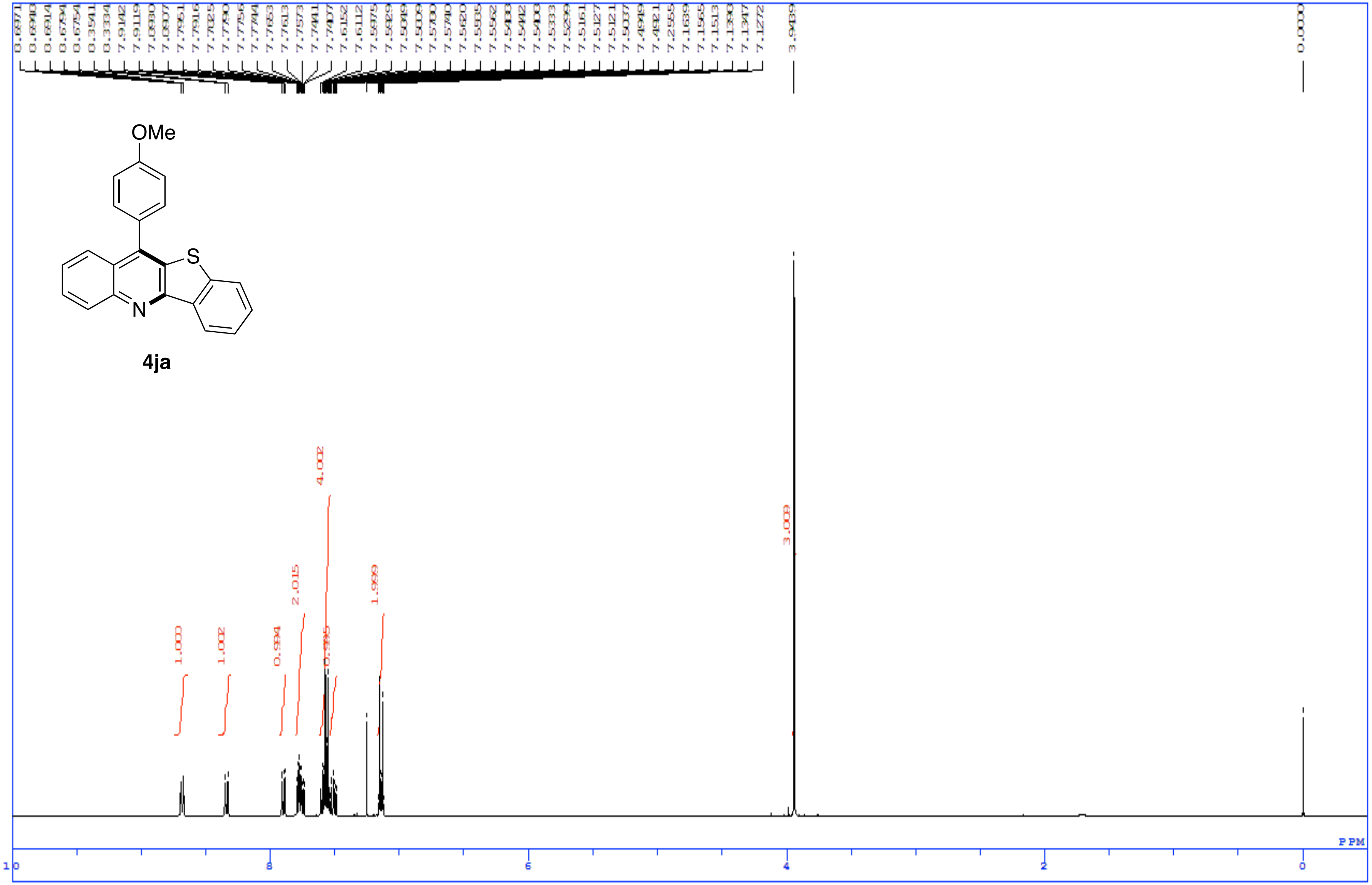

Figure S24. ^{1}H NMR spectrum of compound **4ja** in $CDCl_3$.

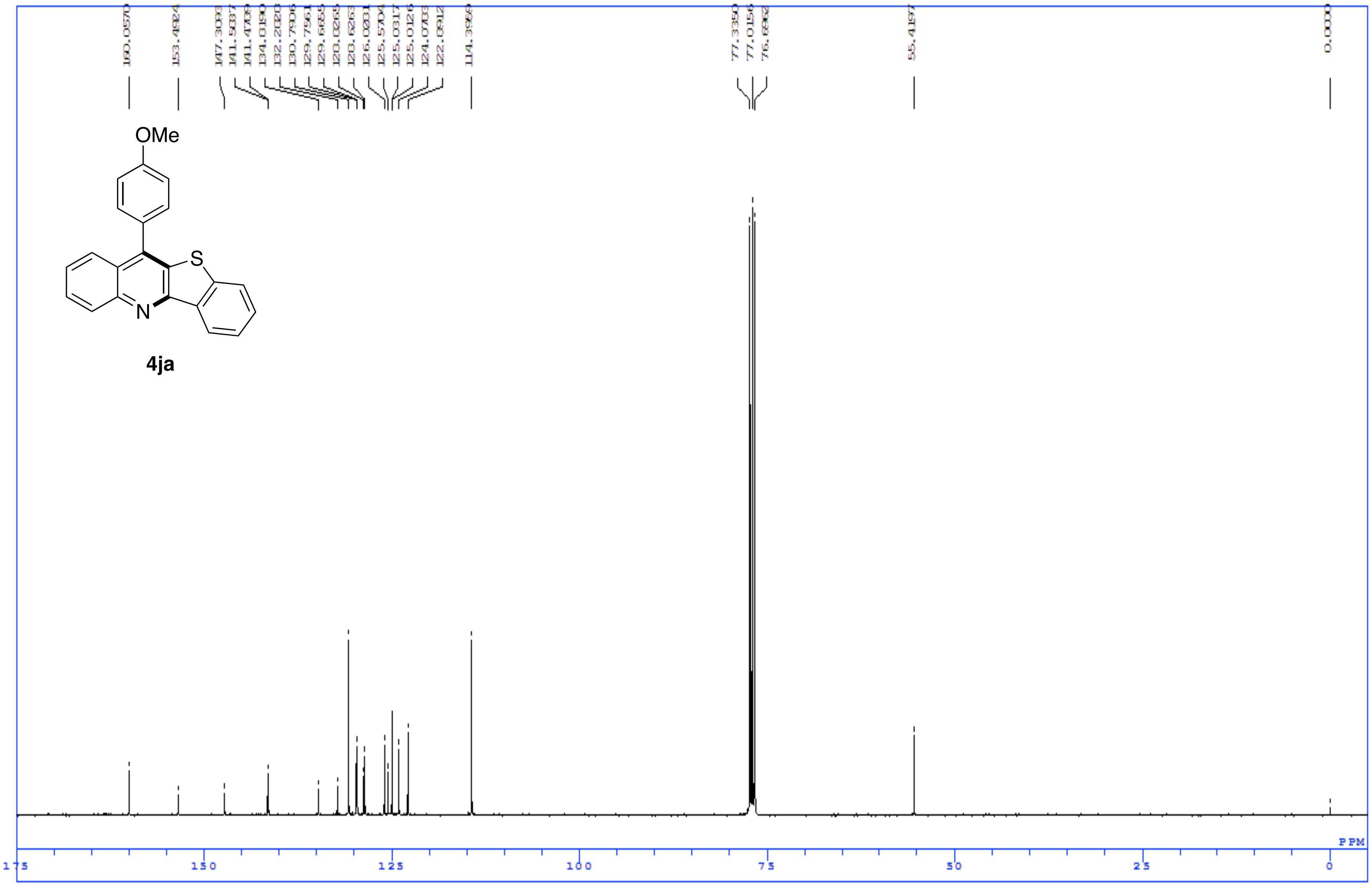

Figure S25. ^{13}C NMR spectrum of compound **4ja** in $CDCl_3$.

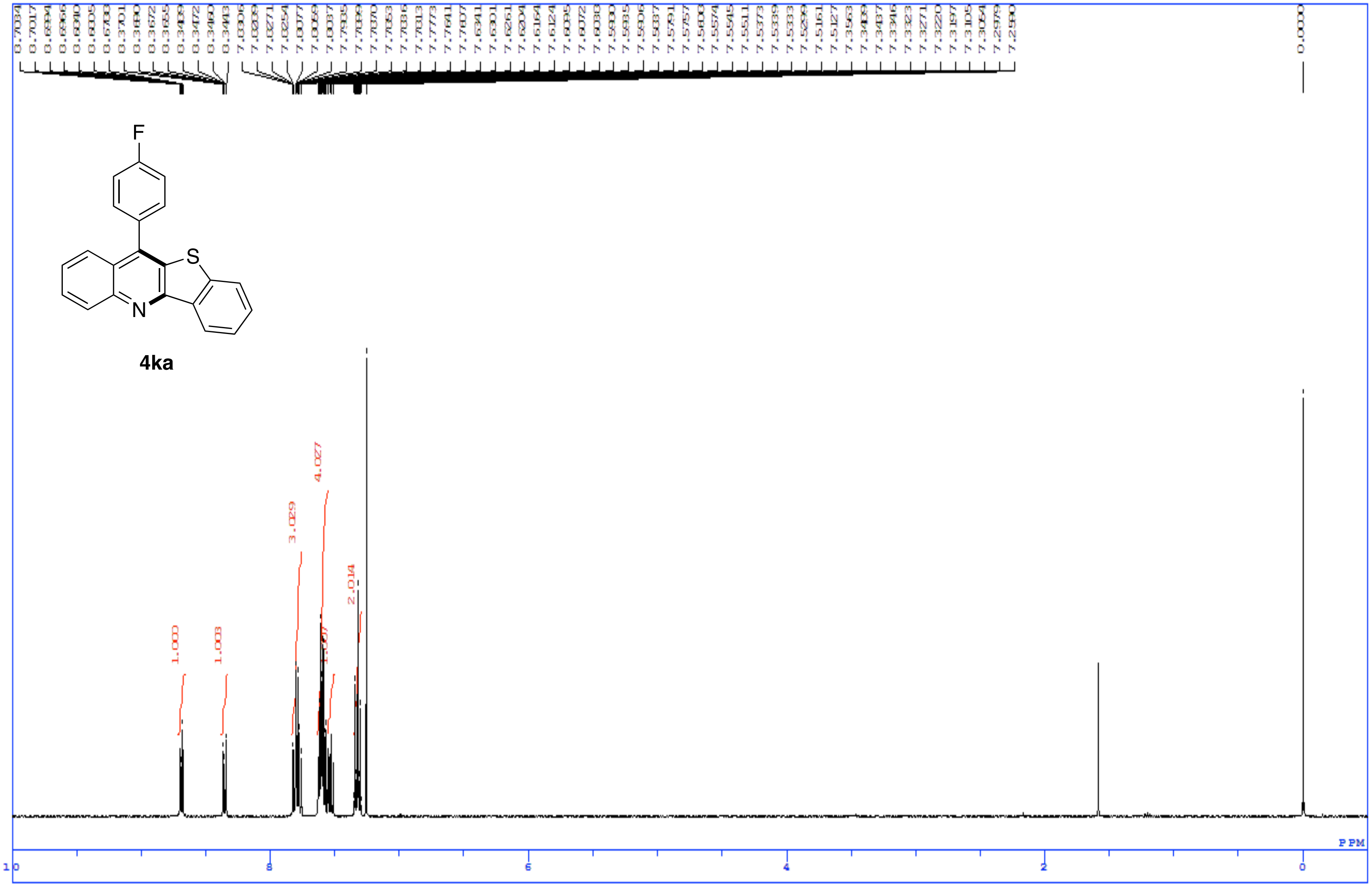

Figure S26. ^{1}H NMR spectrum of compound **4ka** in $CDCl_3$.

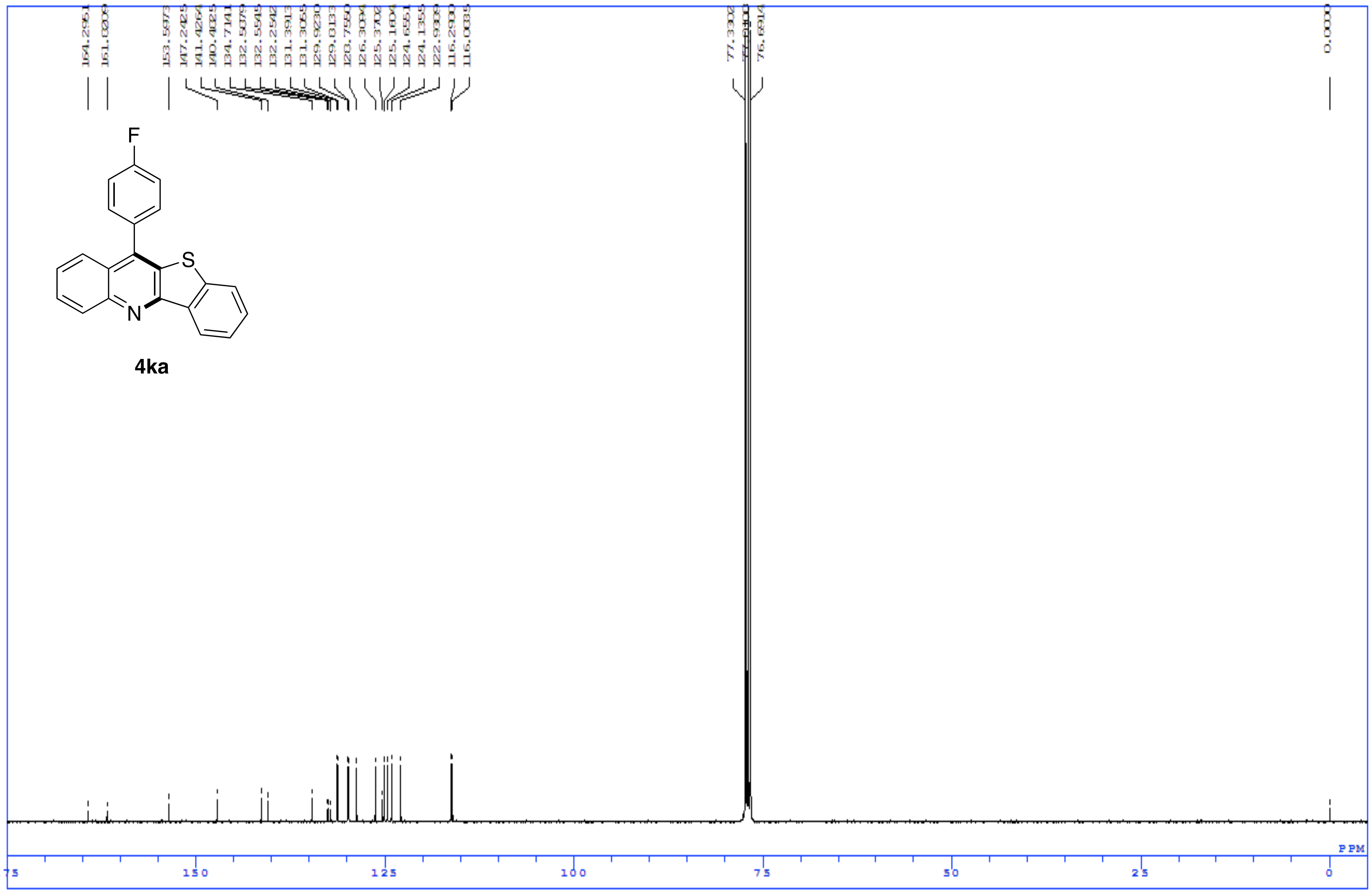

4ka

Figure S27. ^{13}C NMR spectrum of compound **4ka** in $CDCl_3$.

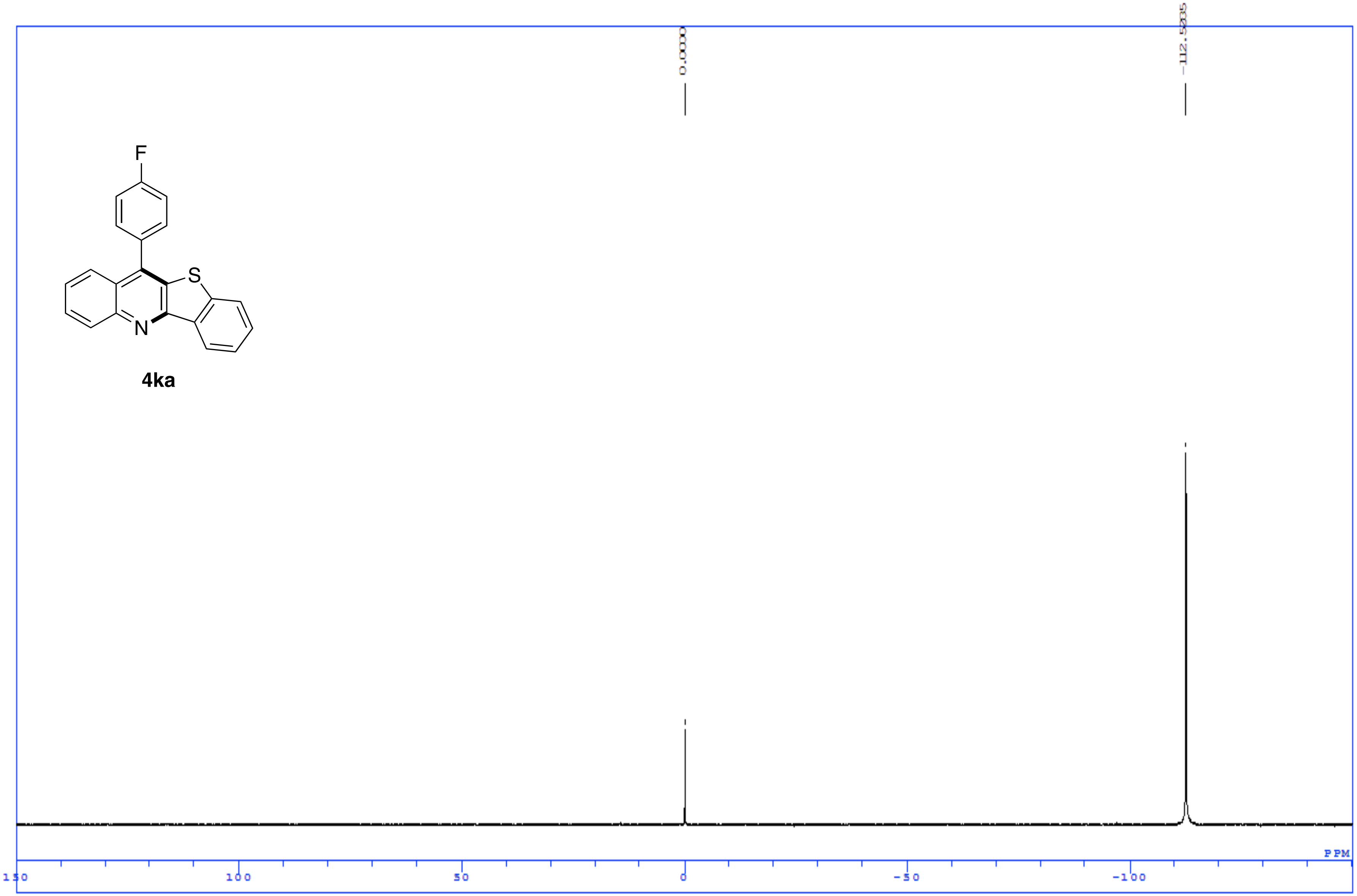

Figure S28. ^{19}F NMR spectrum of compound **4ka** in $CDCl_3$.

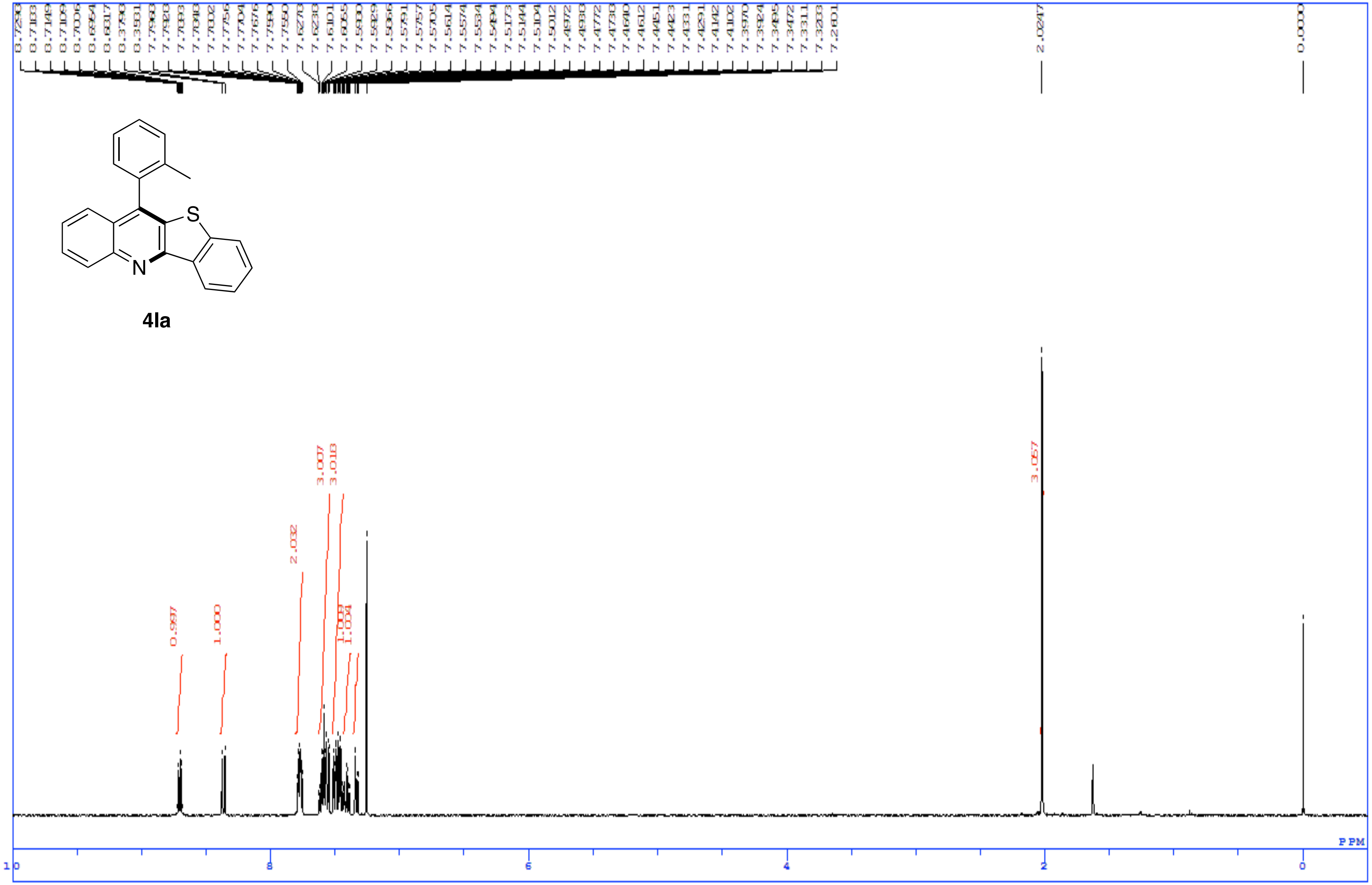

Figure S29. ^{1}H NMR spectrum of compound **4la** in $CDCl_3$.

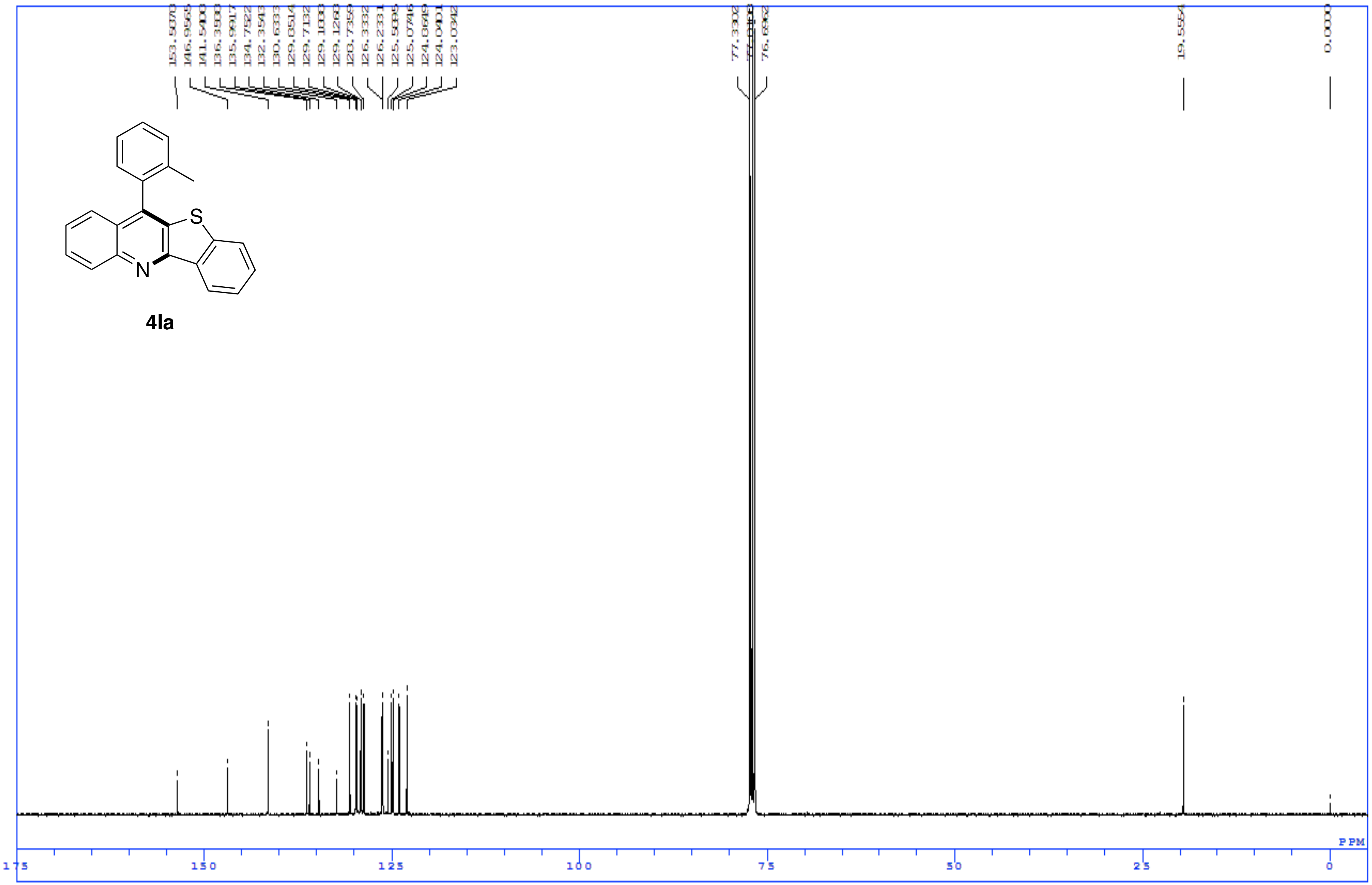

Figure S30. ^{13}C NMR spectrum of compound **4la** in $CDCl_3$.

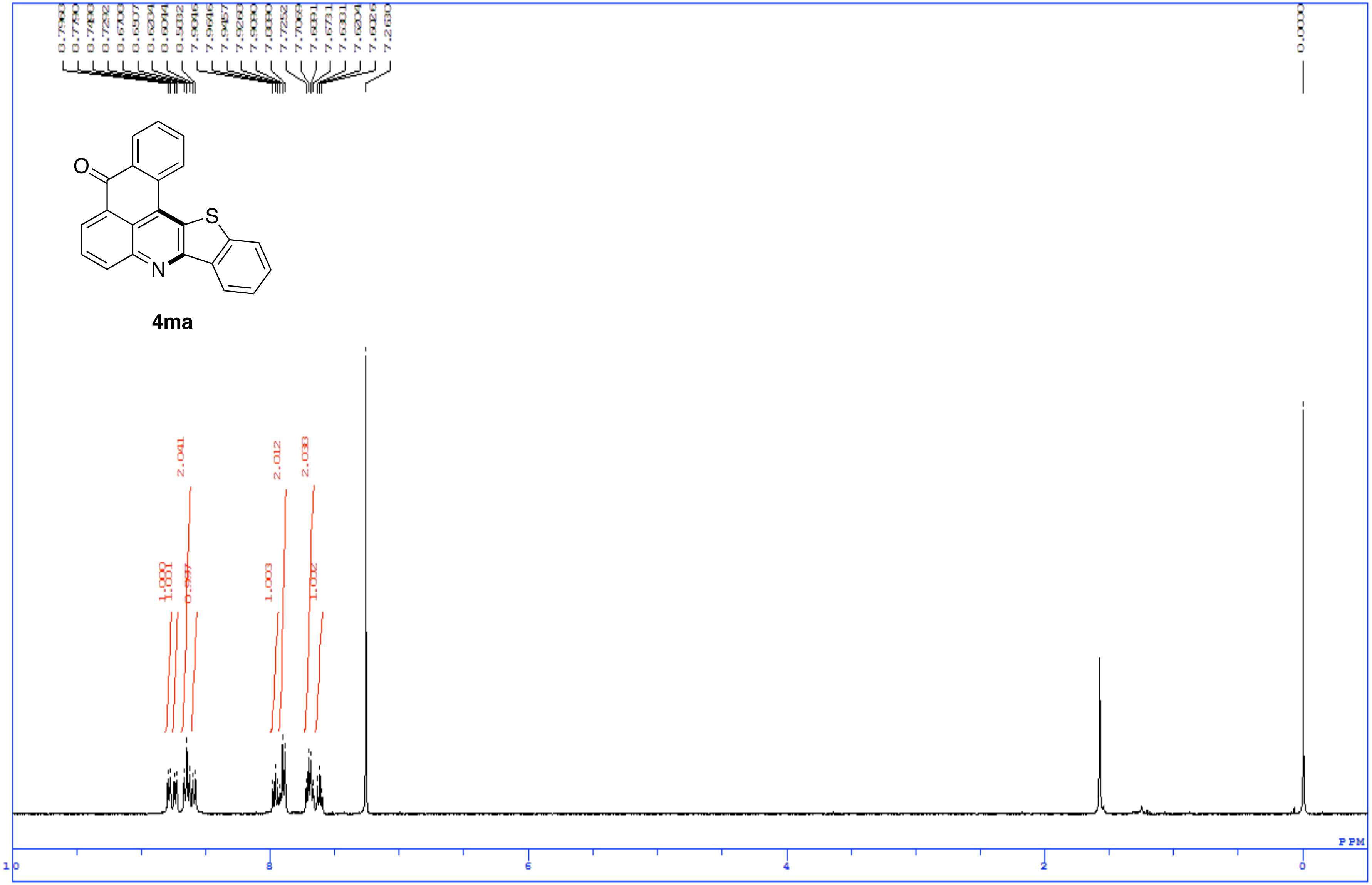

Figure S31. ^{1}H NMR spectrum of compound **4ma** in $CDCl_3$.

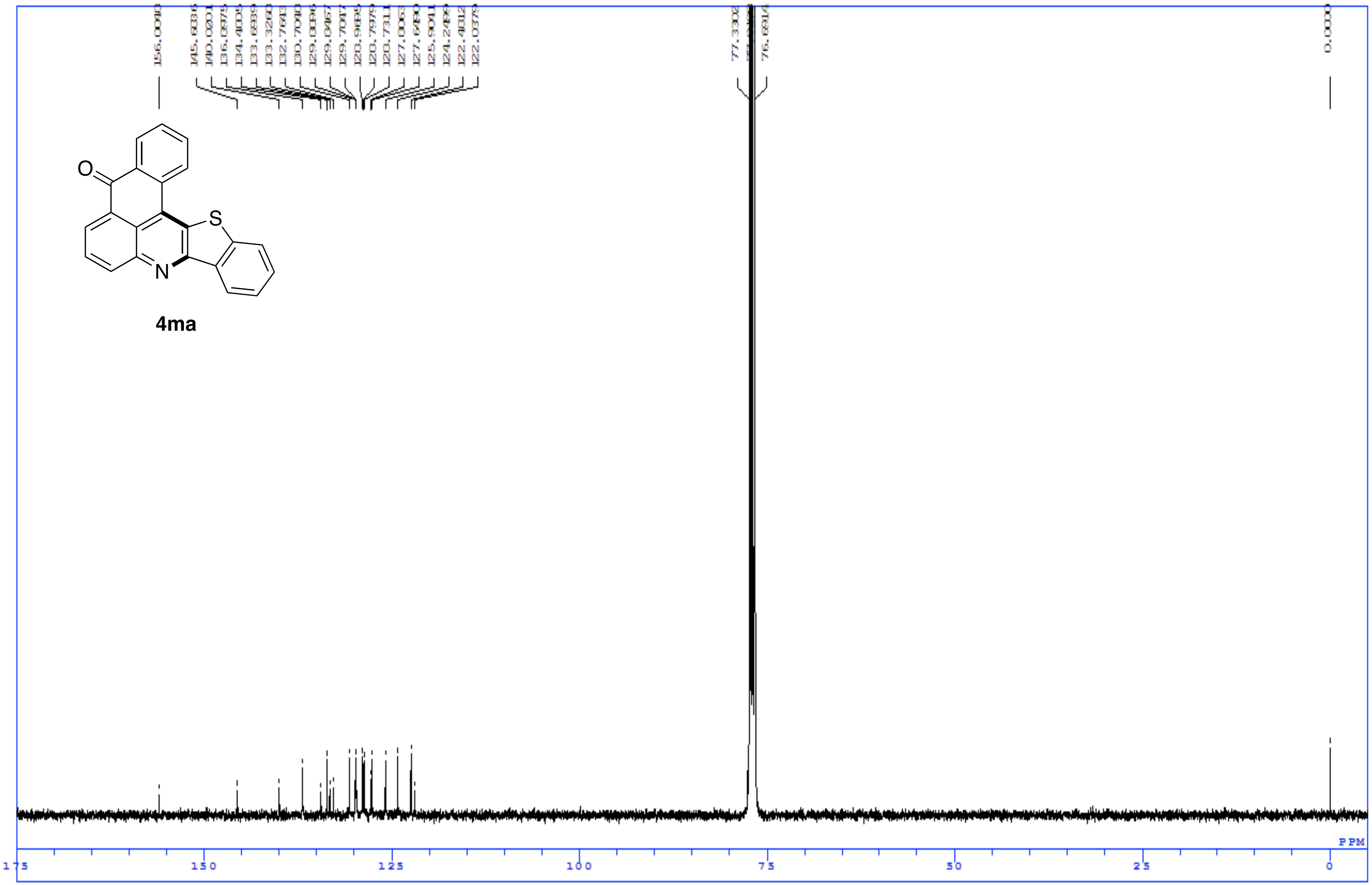

Figure S32. ^{13}C NMR spectrum of compound **4ma** in $CDCl_3$.

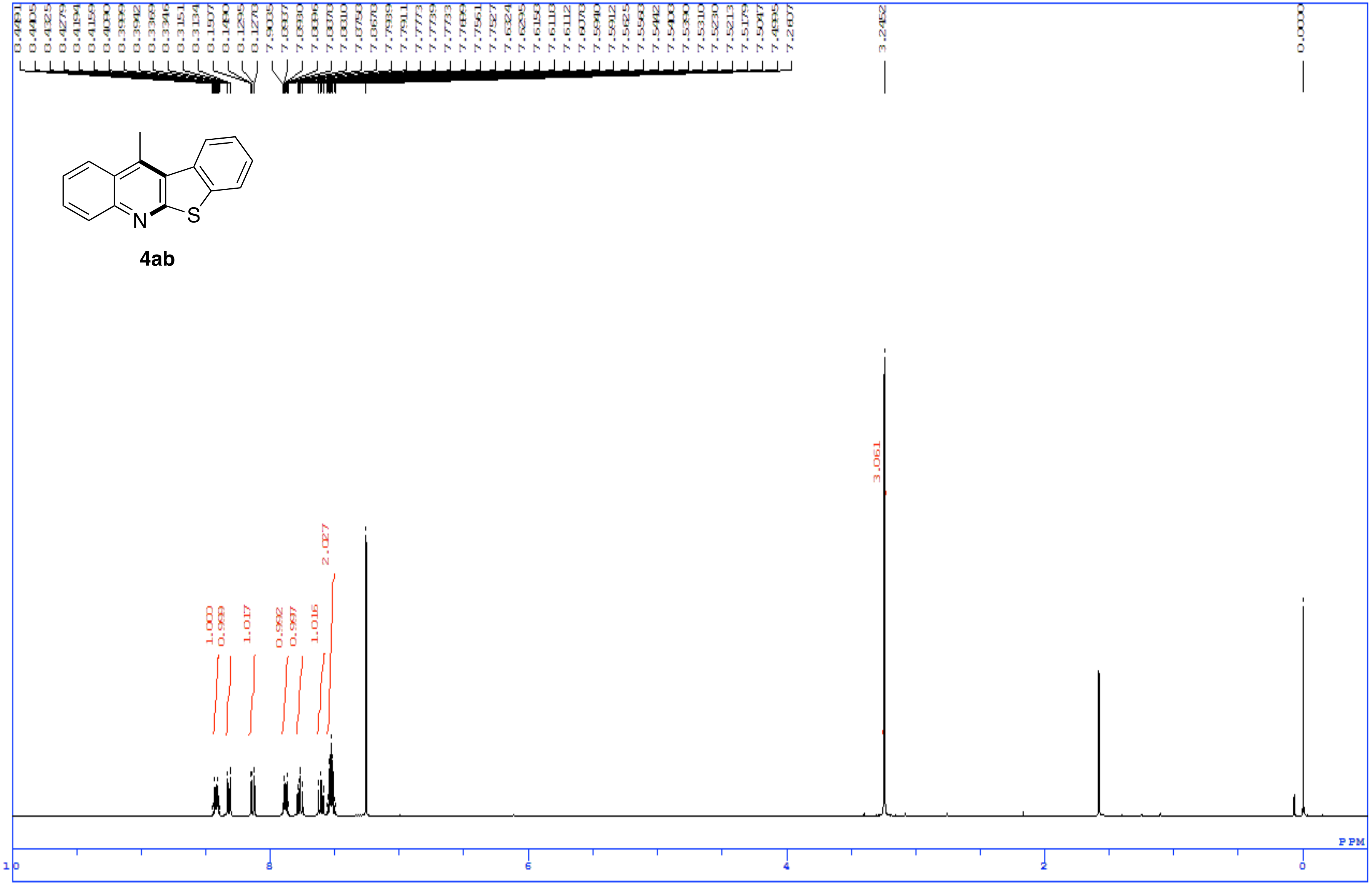

Figure S33. ^{1}H NMR spectrum of compound **4ab** in $CDCl_3$.

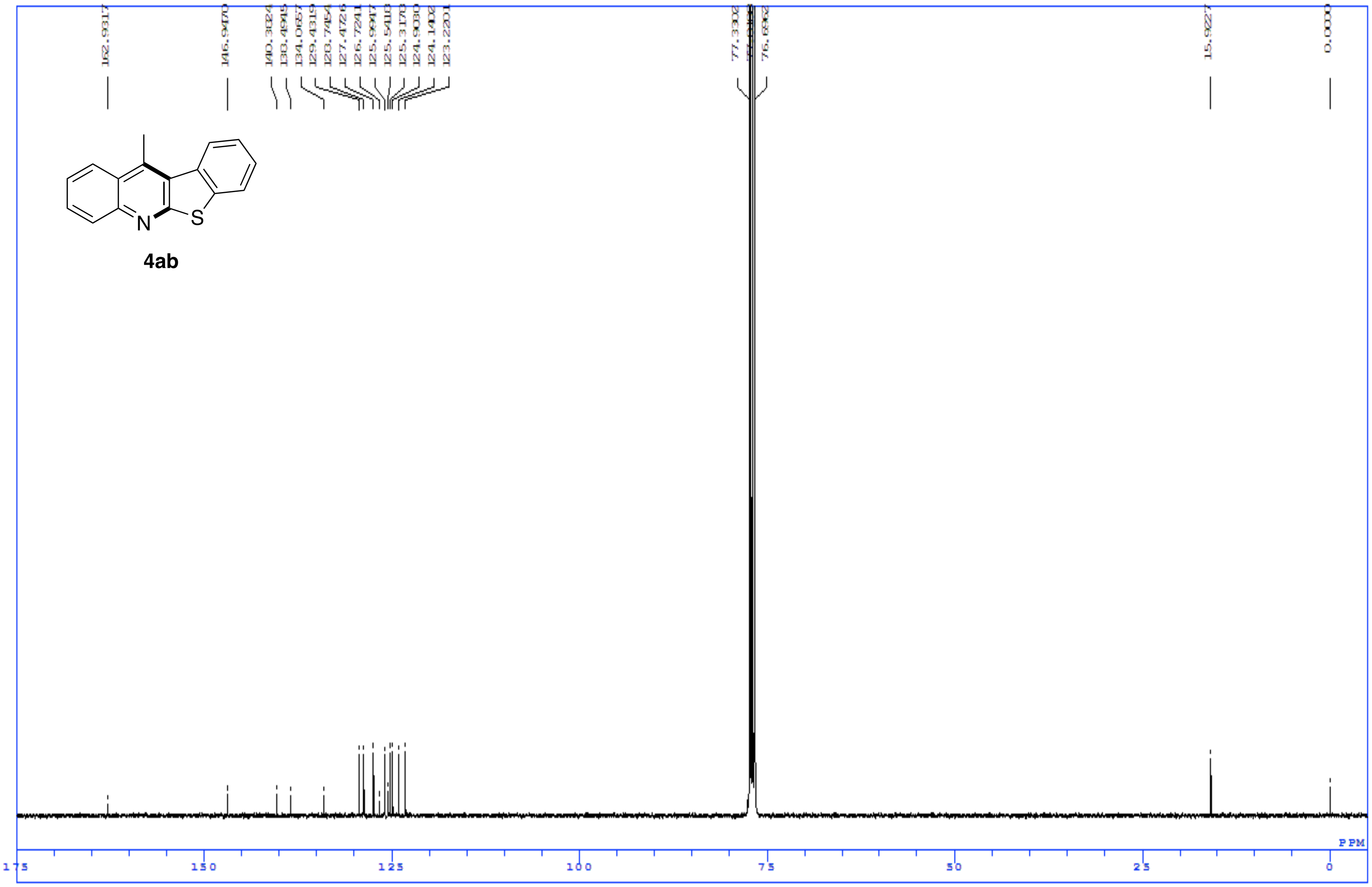

Figure S34. ^{13}C NMR spectrum of compound **4ab** in $CDCl_3$.

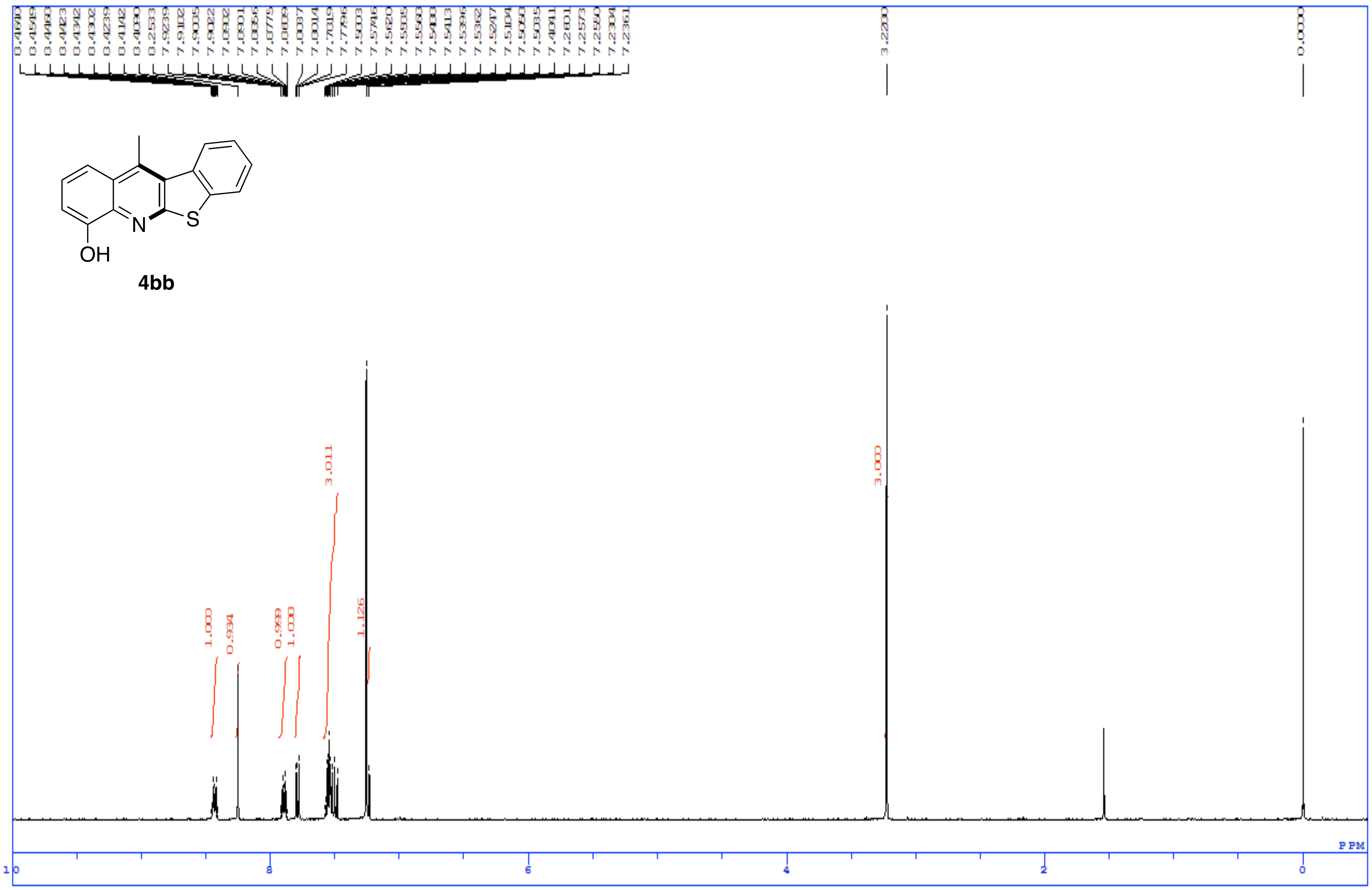

Figure S35. ^{1}H NMR spectrum of compound **4bb** in $CDCl_3$.

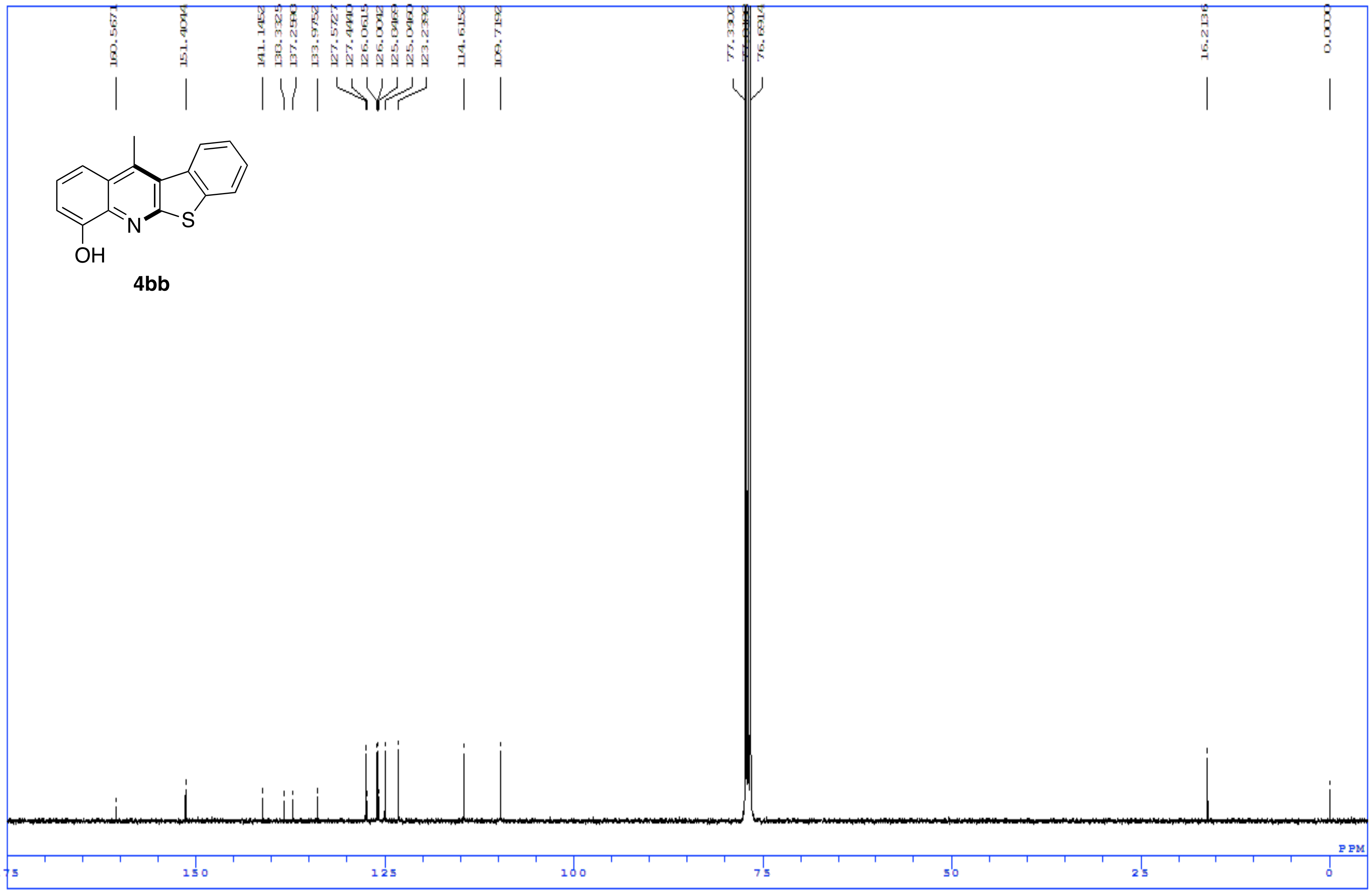

Figure S36. ^{13}C NMR spectrum of compound **4bb** in $CDCl_3$.

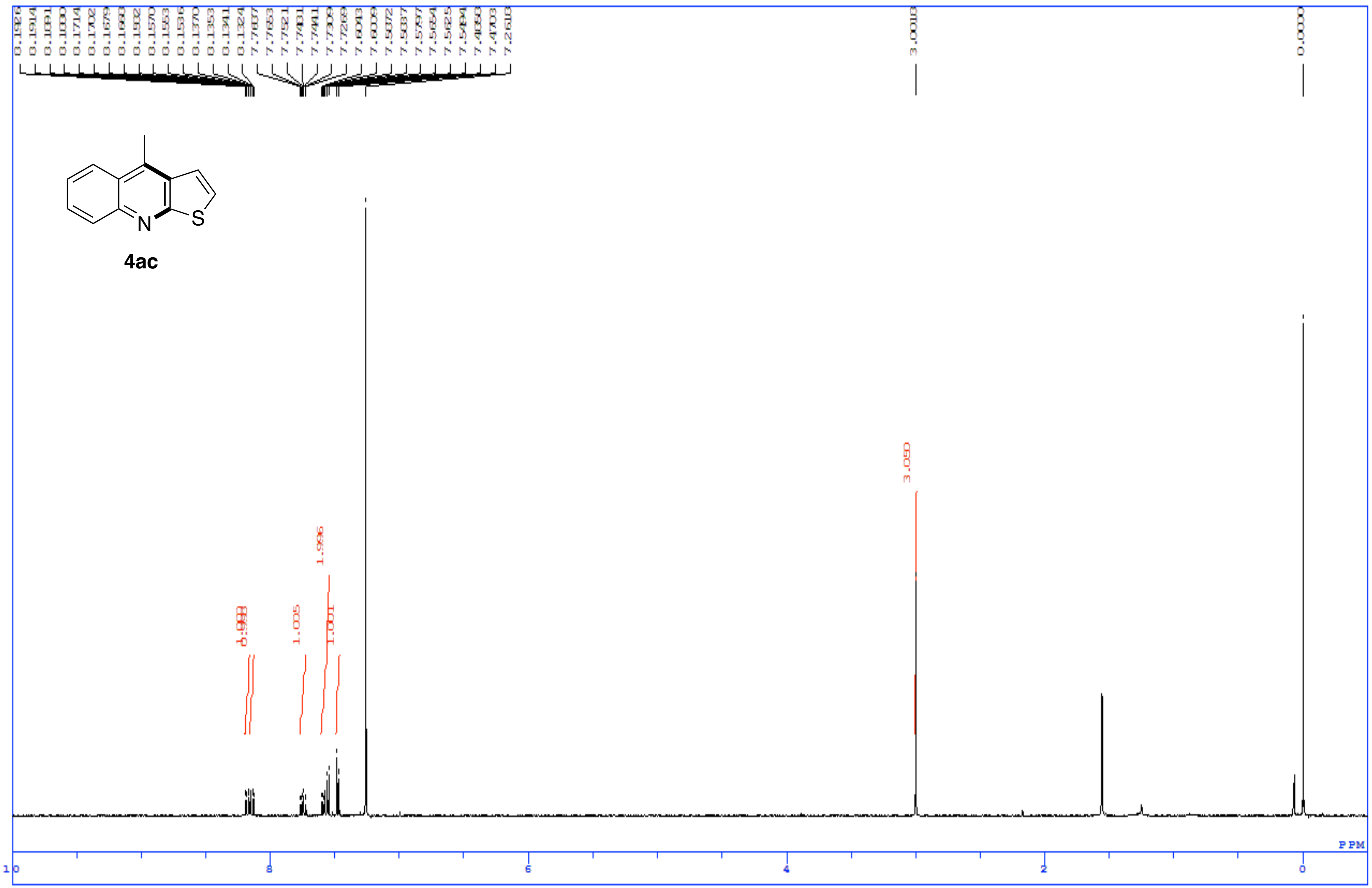

Figure S37. ^{1}H NMR spectrum of compound **4ac** in $CDCl_3$.

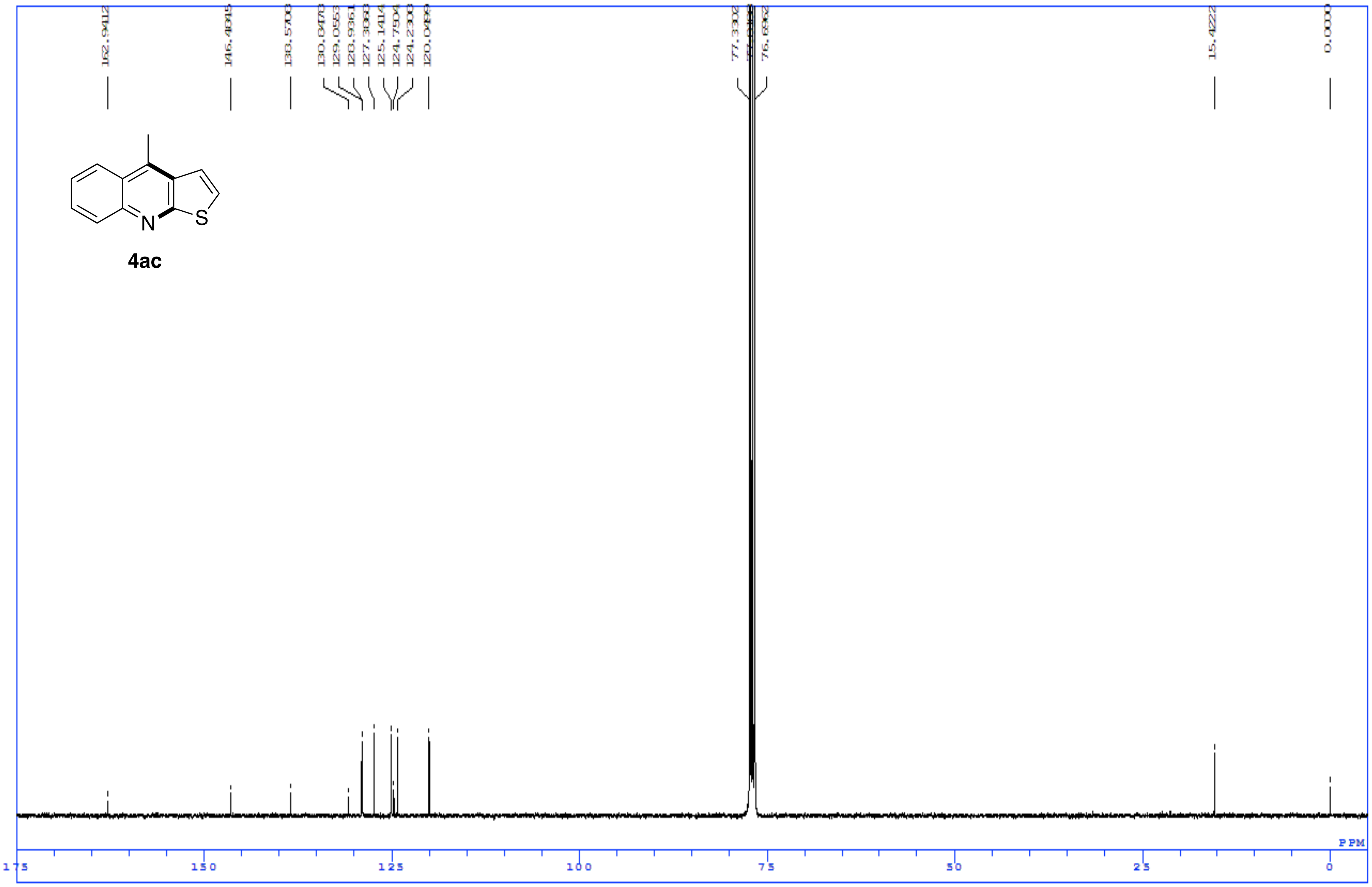

Figure S38. ^{13}C NMR spectrum of compound **4ac** in $CDCl_3$.

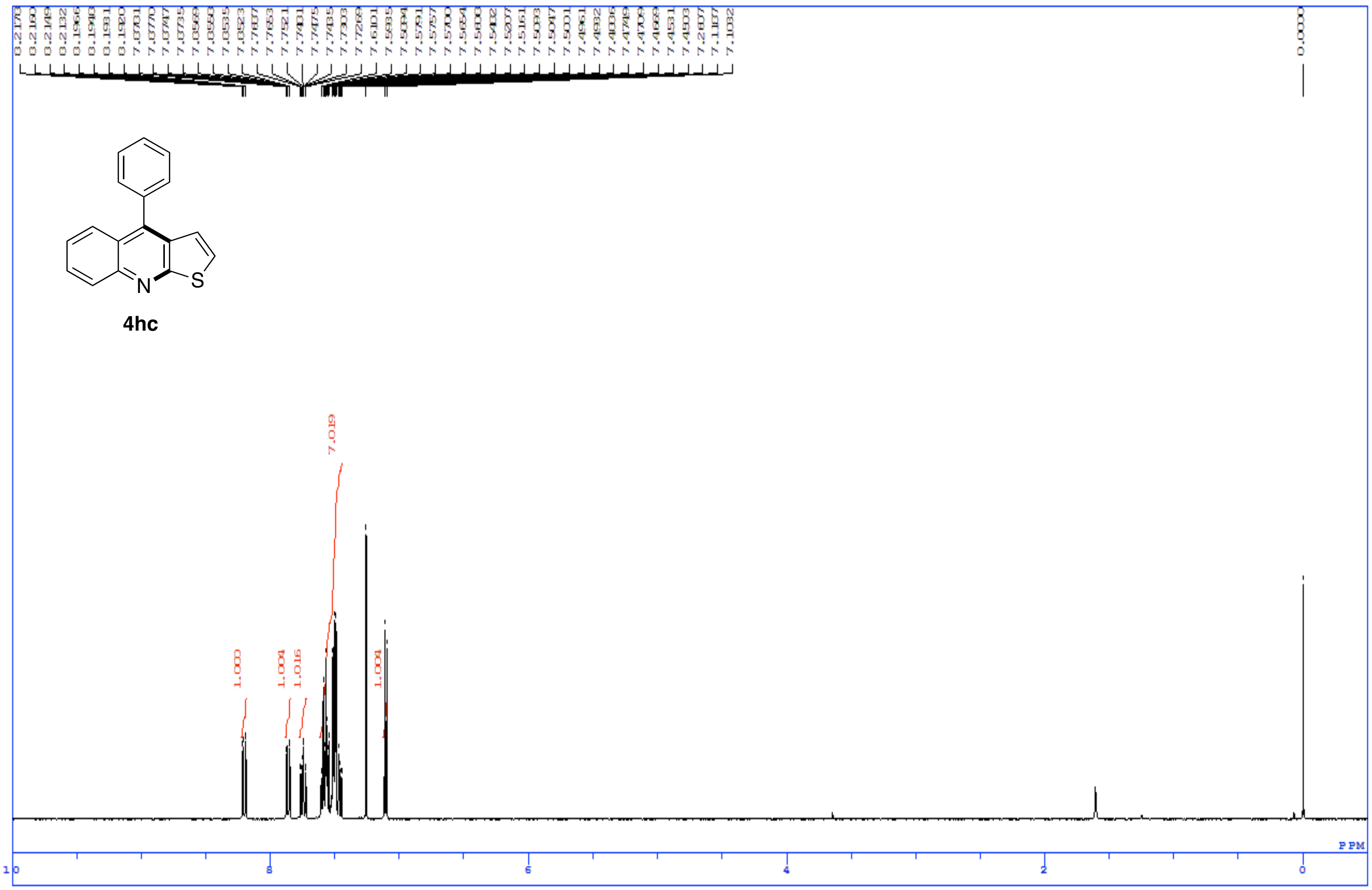

Figure S39. ^{1}H NMR spectrum of compound **4hc** in $CDCl_3$.

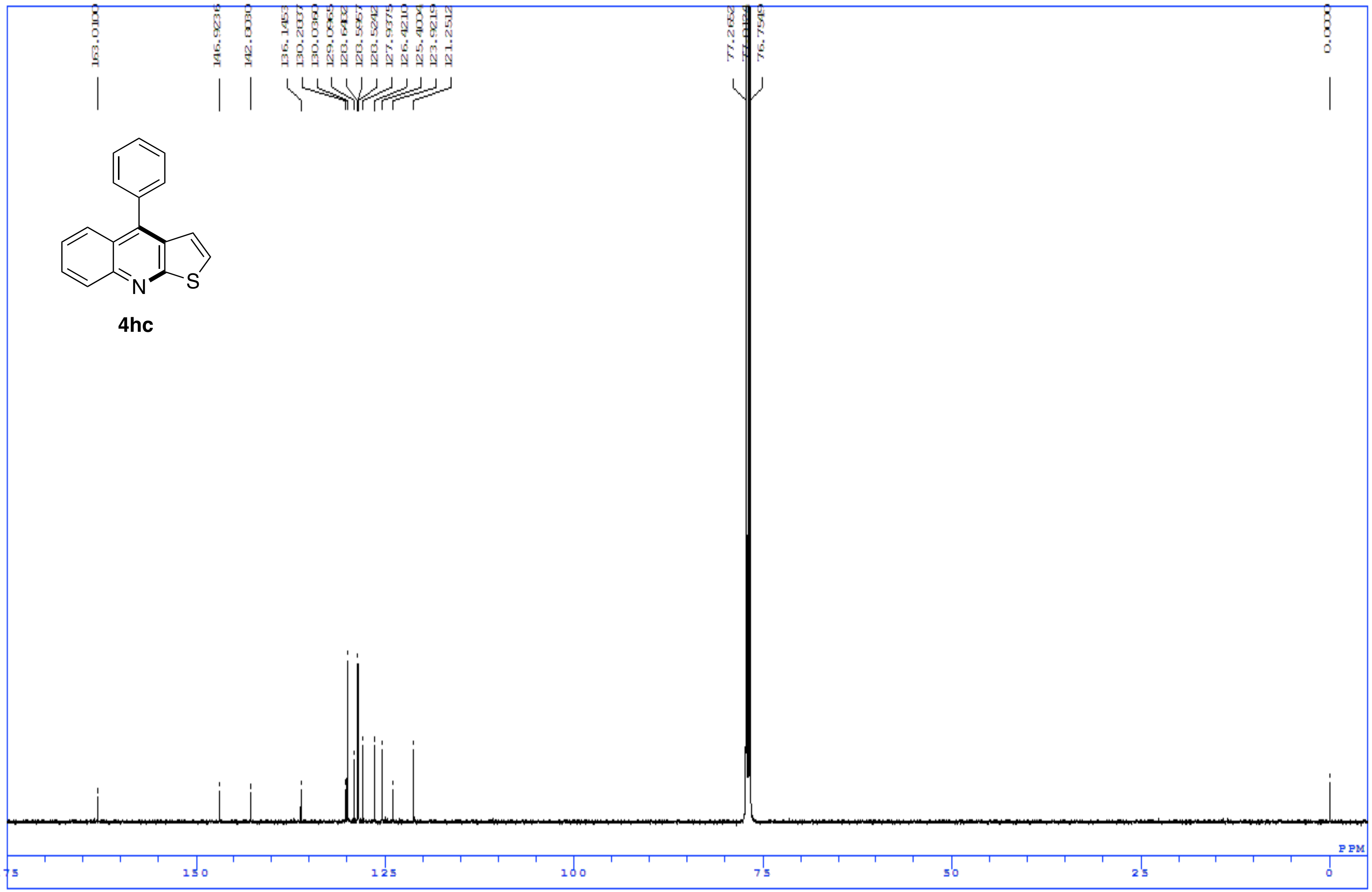

Figure S40. ^{13}C NMR spectrum of compound **4hc** in $CDCl_3$.

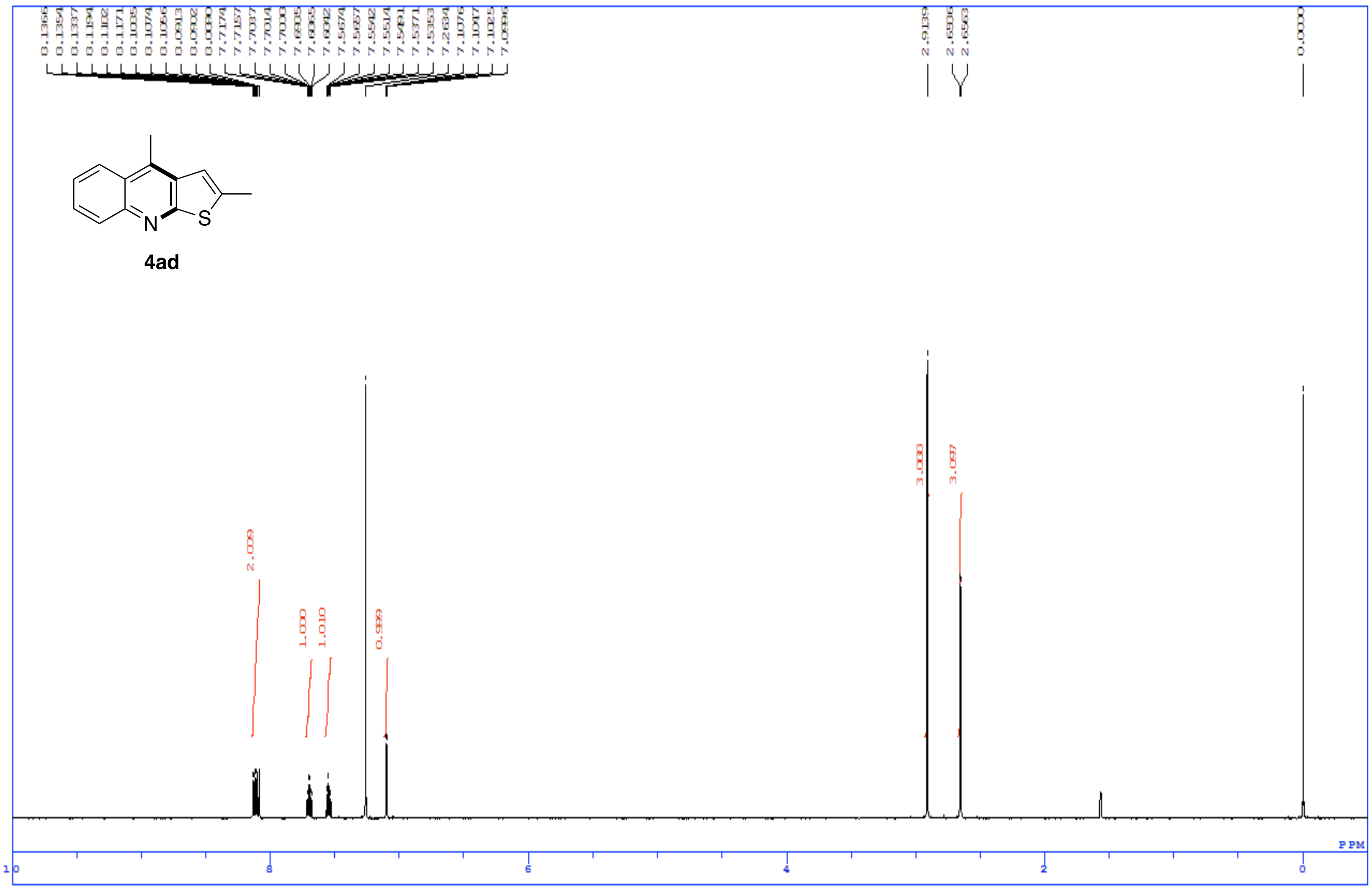

Figure S41. ^{1}H NMR spectrum of compound **4ad** in $CDCl_3$.

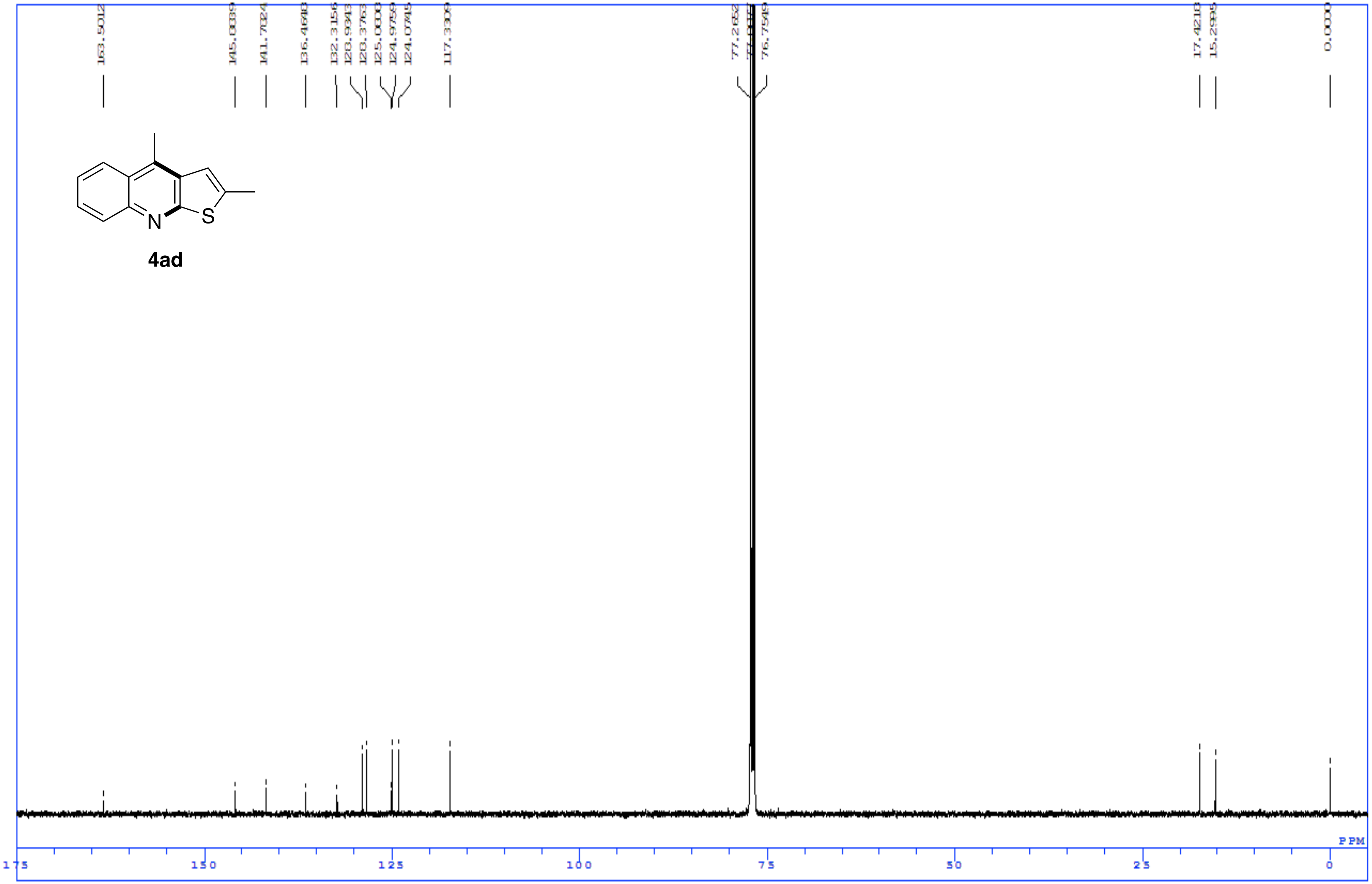

Figure S42. ^{13}C NMR spectrum of compound **4ad** in $CDCl_3$.

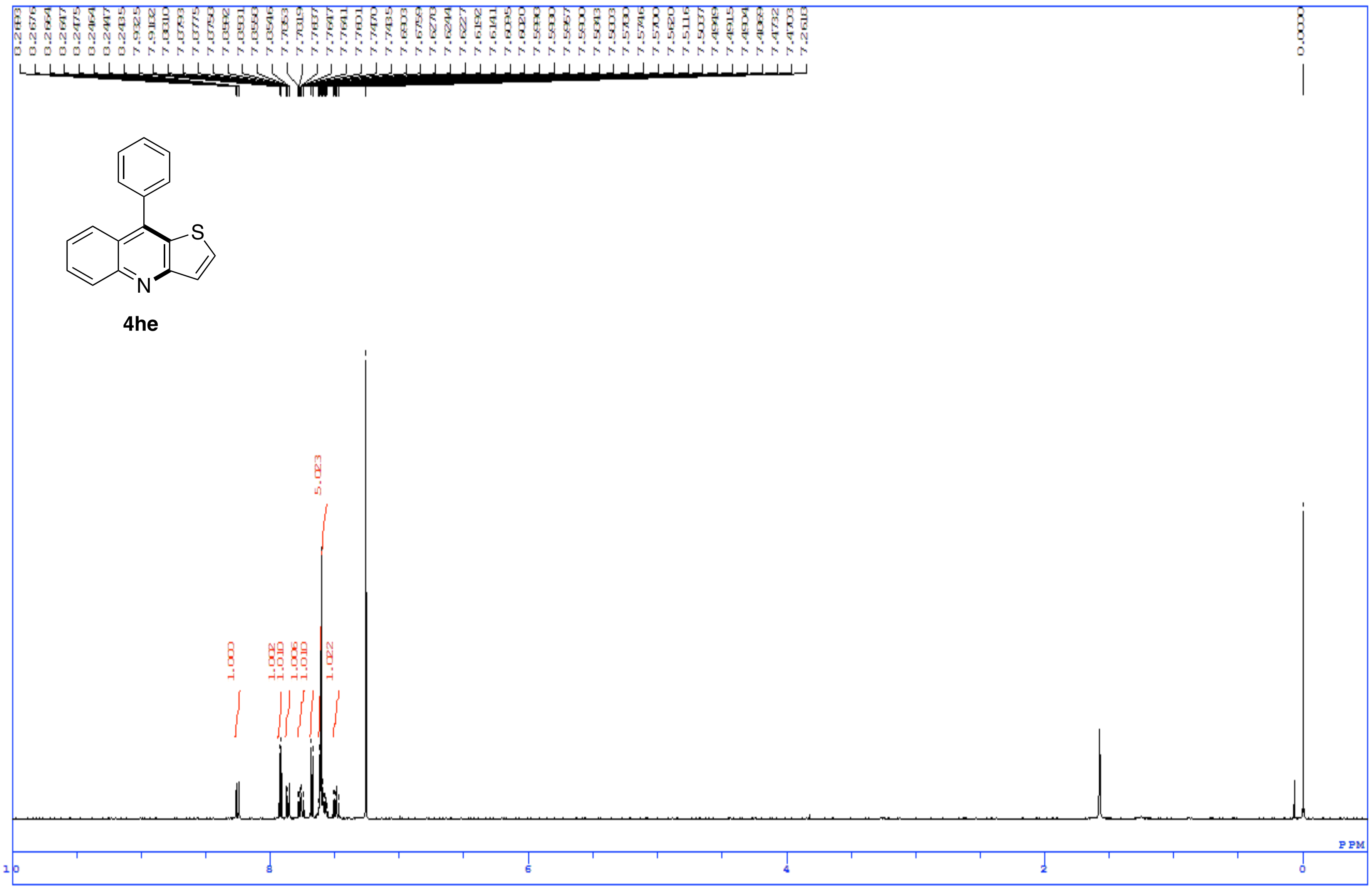

Figure S43. ^{1}H NMR spectrum of compound **4he** in $CDCl_3$.

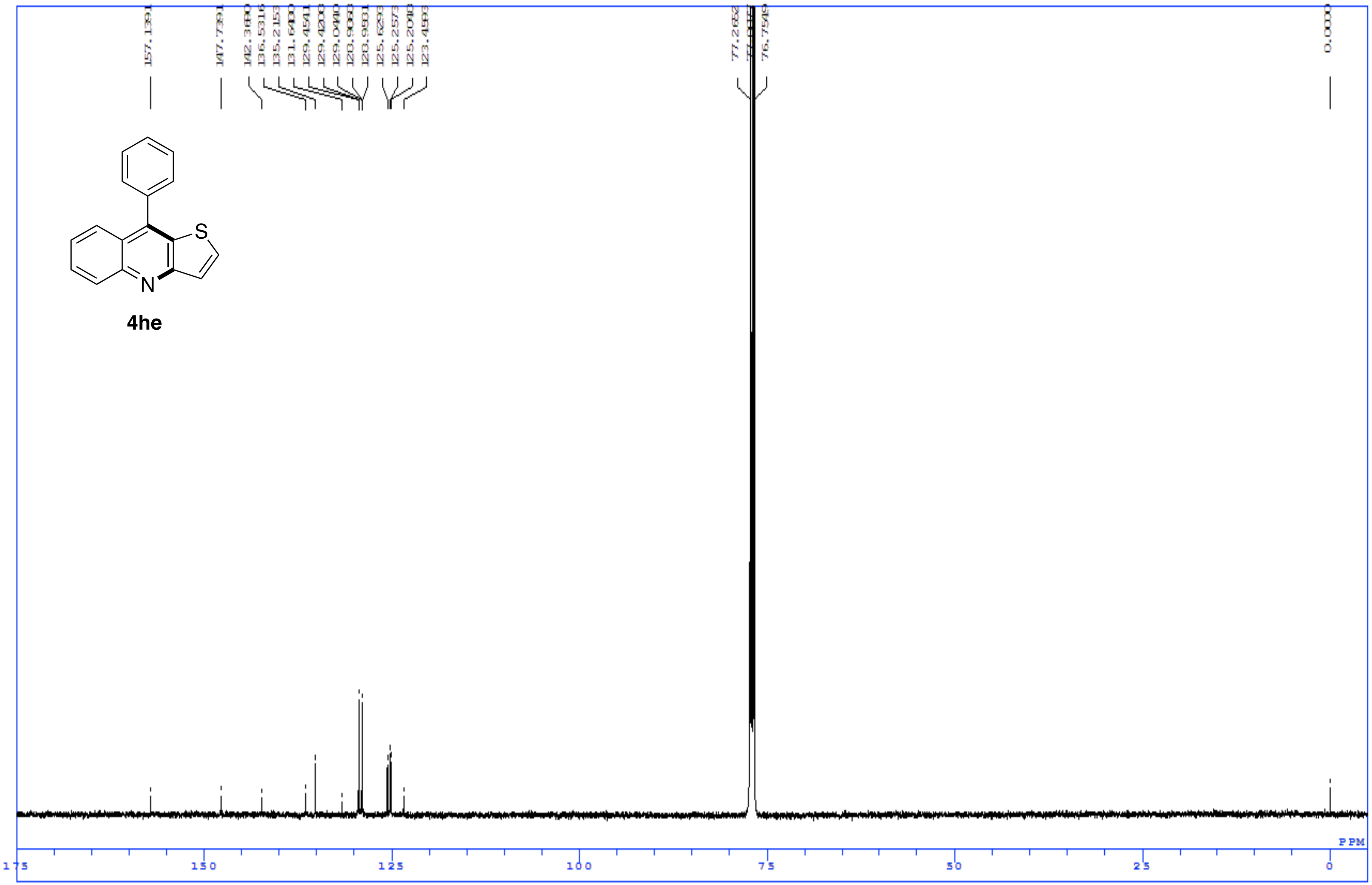

Figure S44. ^{13}C NMR spectrum of compound **4he** in $CDCl_3$.

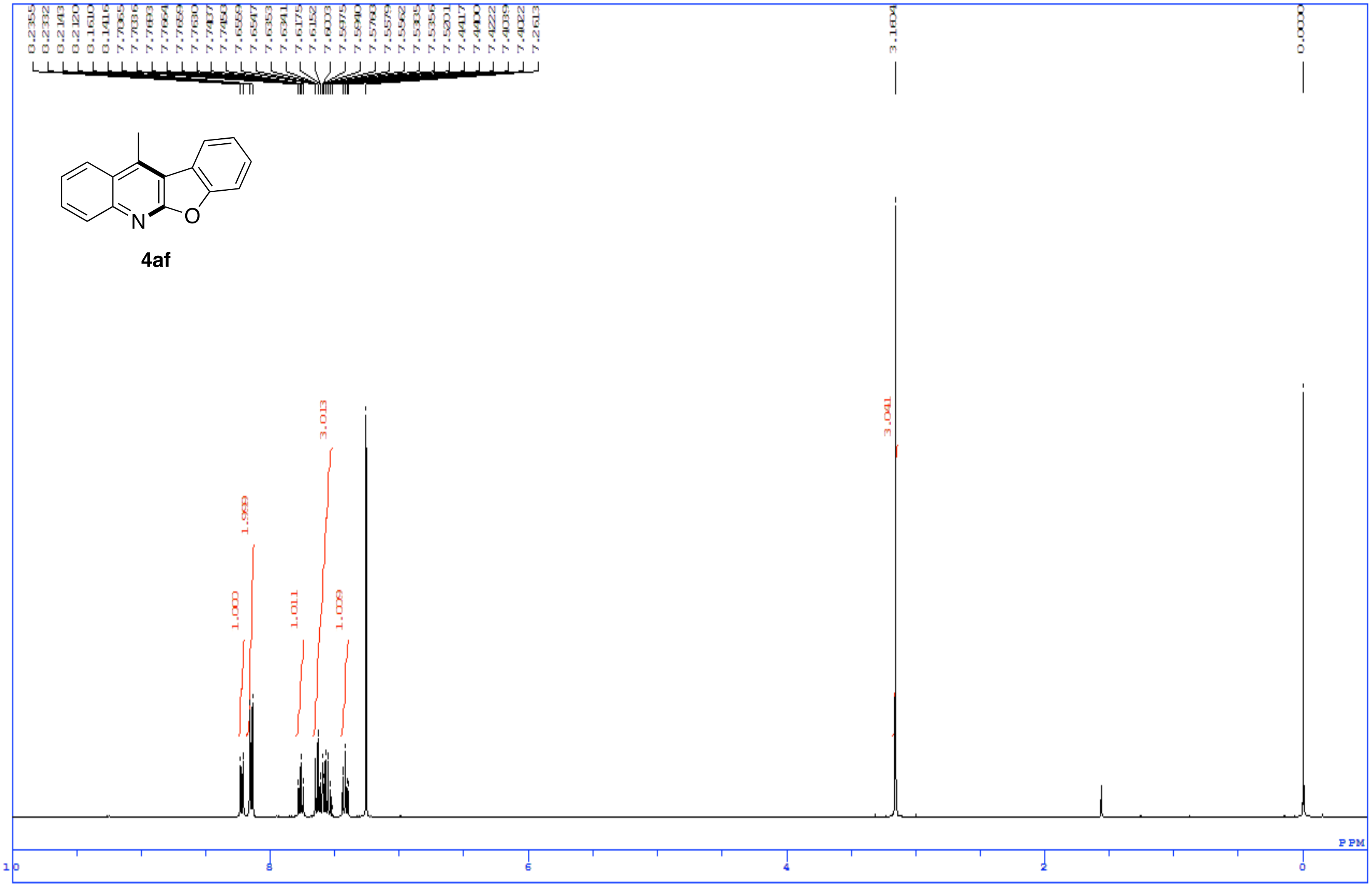

Figure S45. ^{1}H NMR spectrum of compound **4af** in $CDCl_3$.

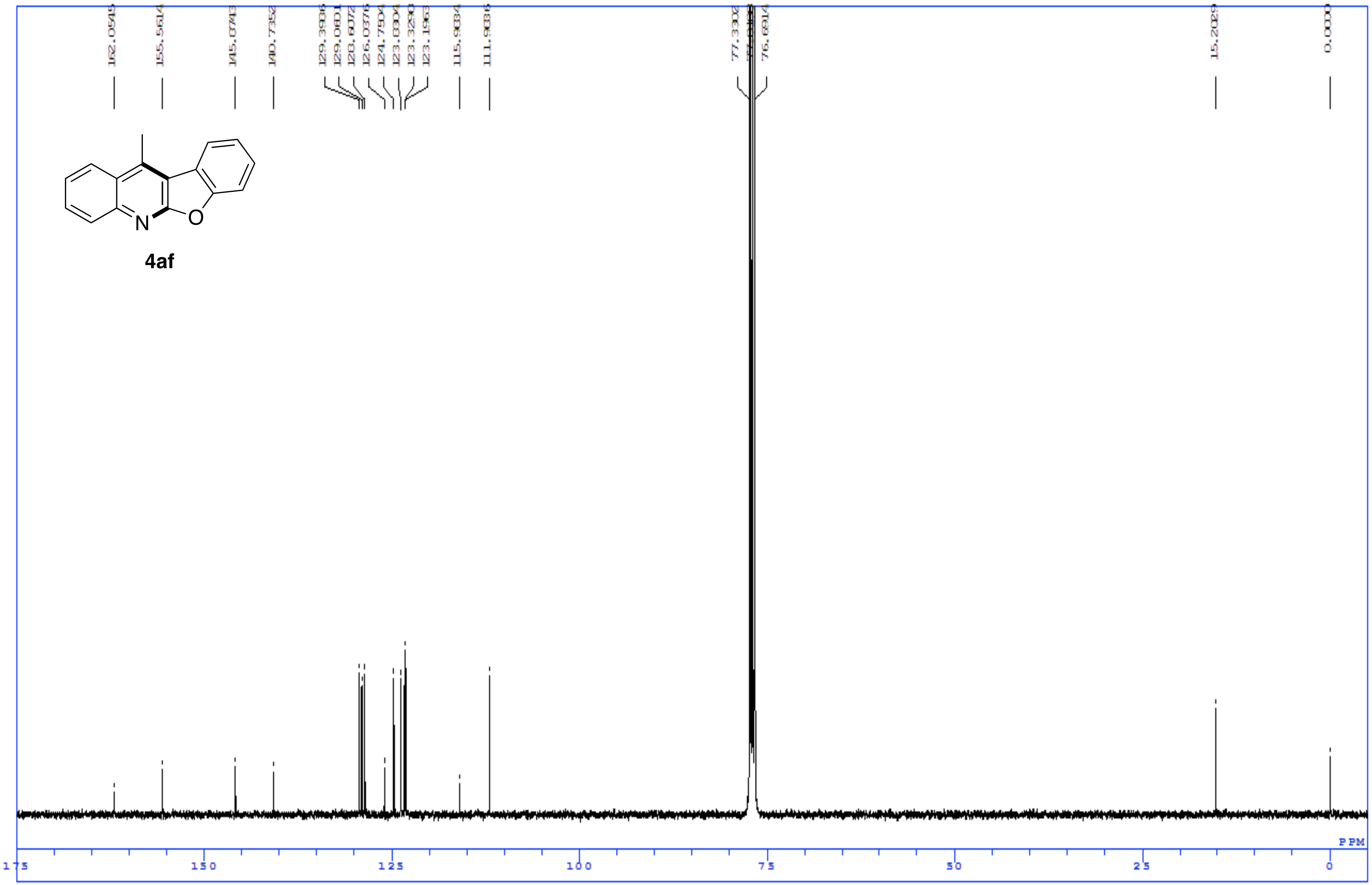

Figure S46. ^{13}C NMR spectrum of compound **4af** in $CDCl_3$.

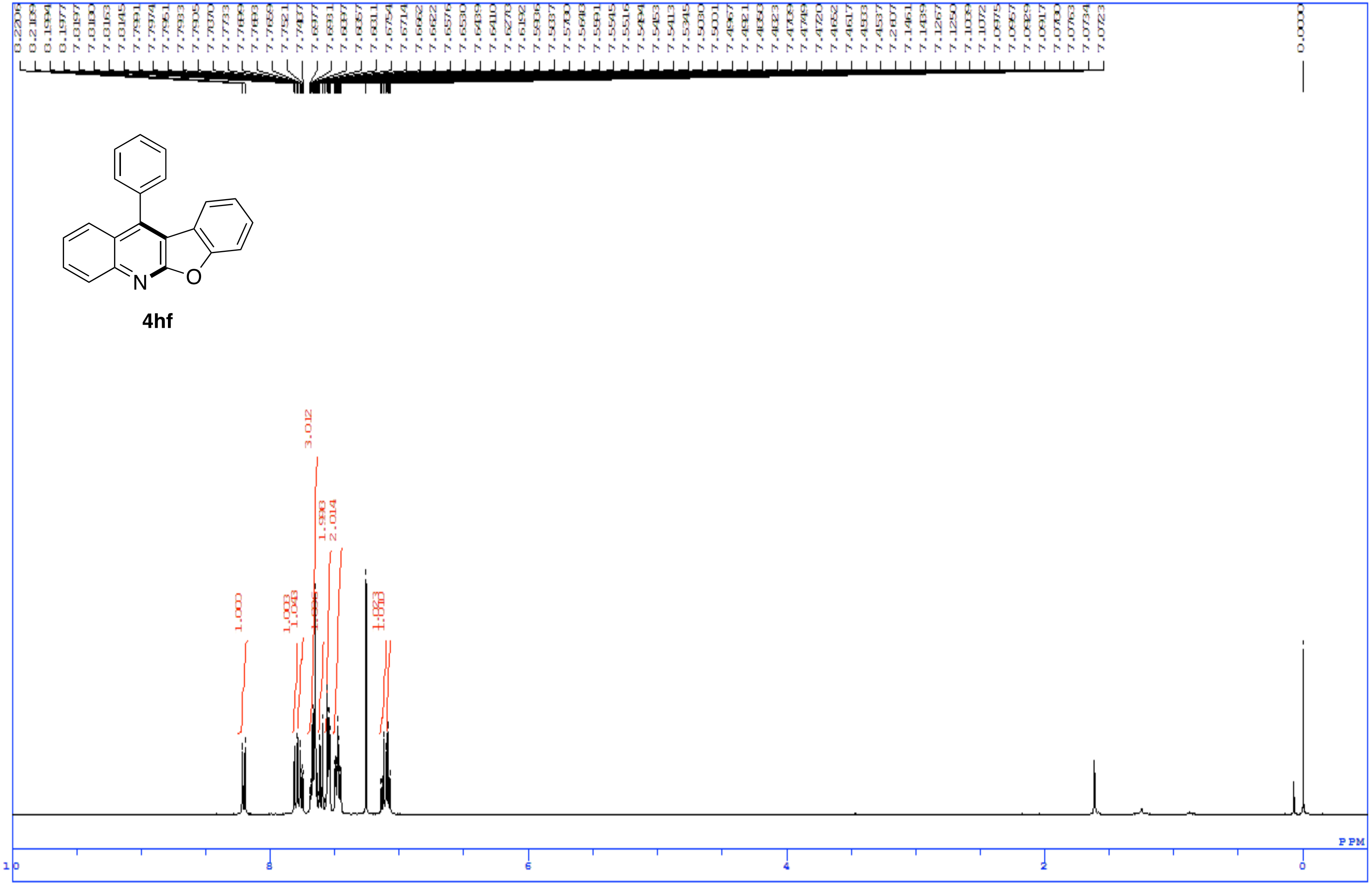

4hf

Figure S47. ^{1}H NMR spectrum of compound **4hf** in $CDCl_3$.

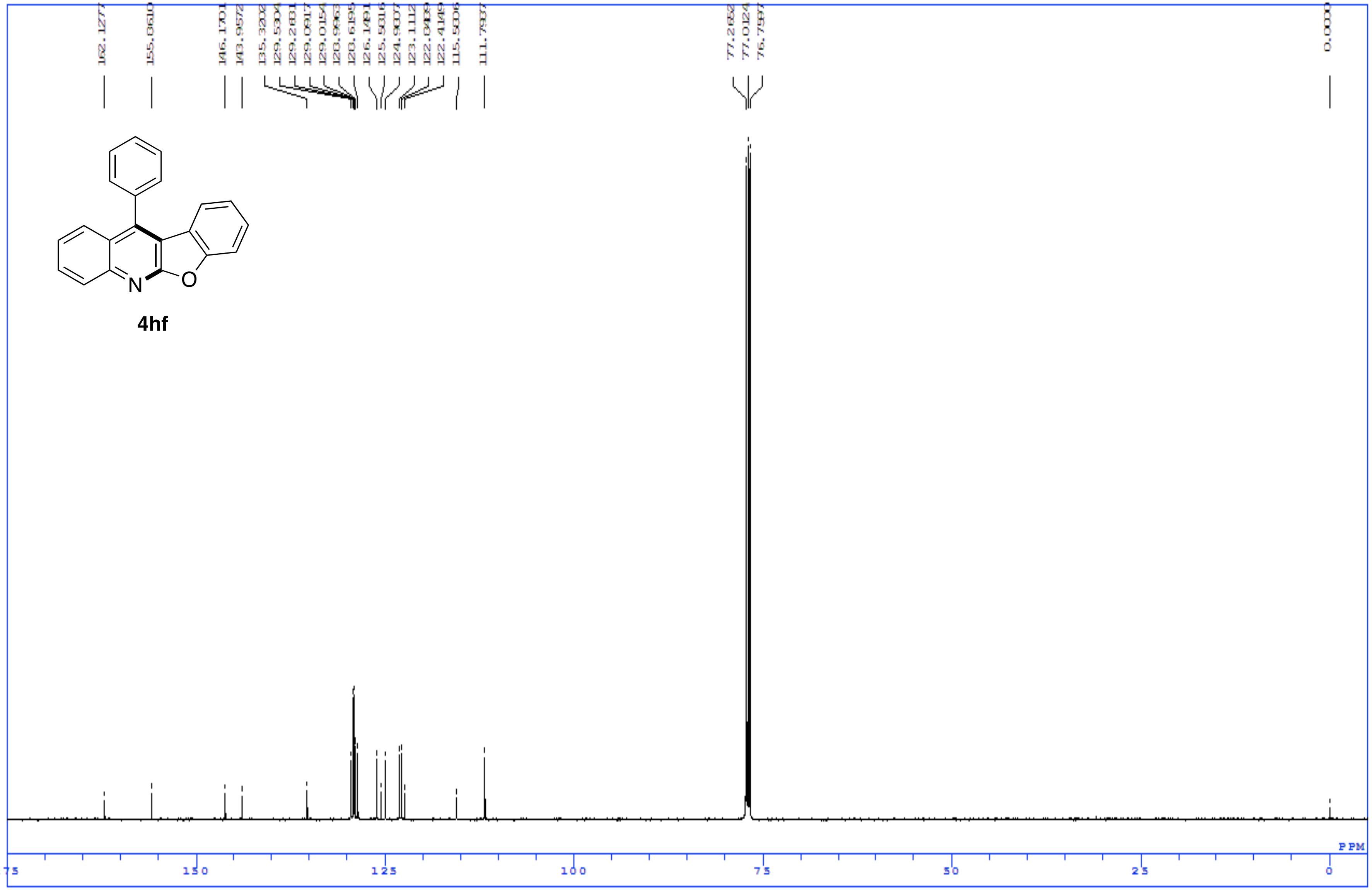

Figure S48. ^{13}C NMR spectrum of compound **4hf** in $CDCl_3$.

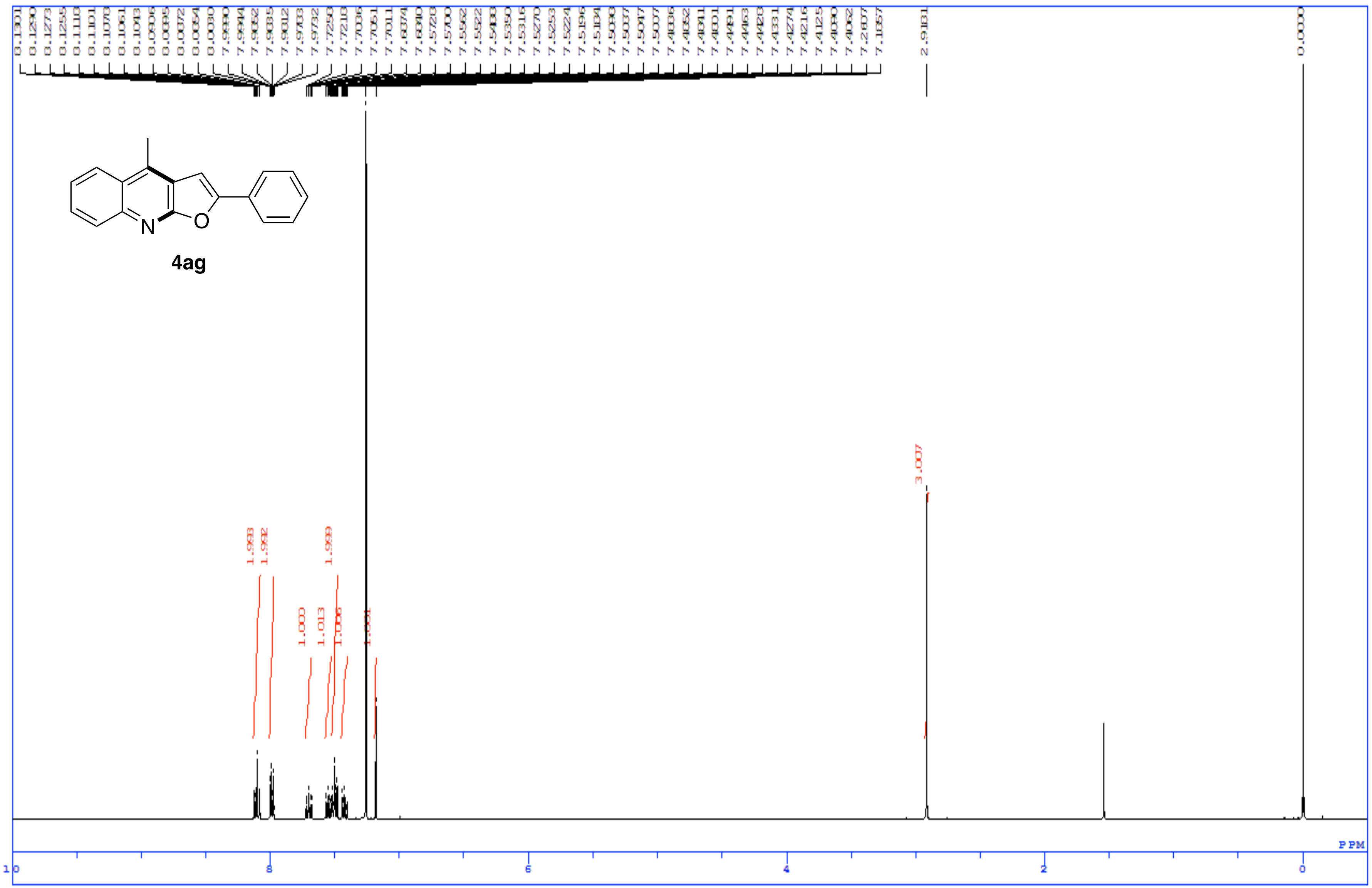

Figure S49. ^{1}H NMR spectrum of compound **4ag** in $CDCl_3$.

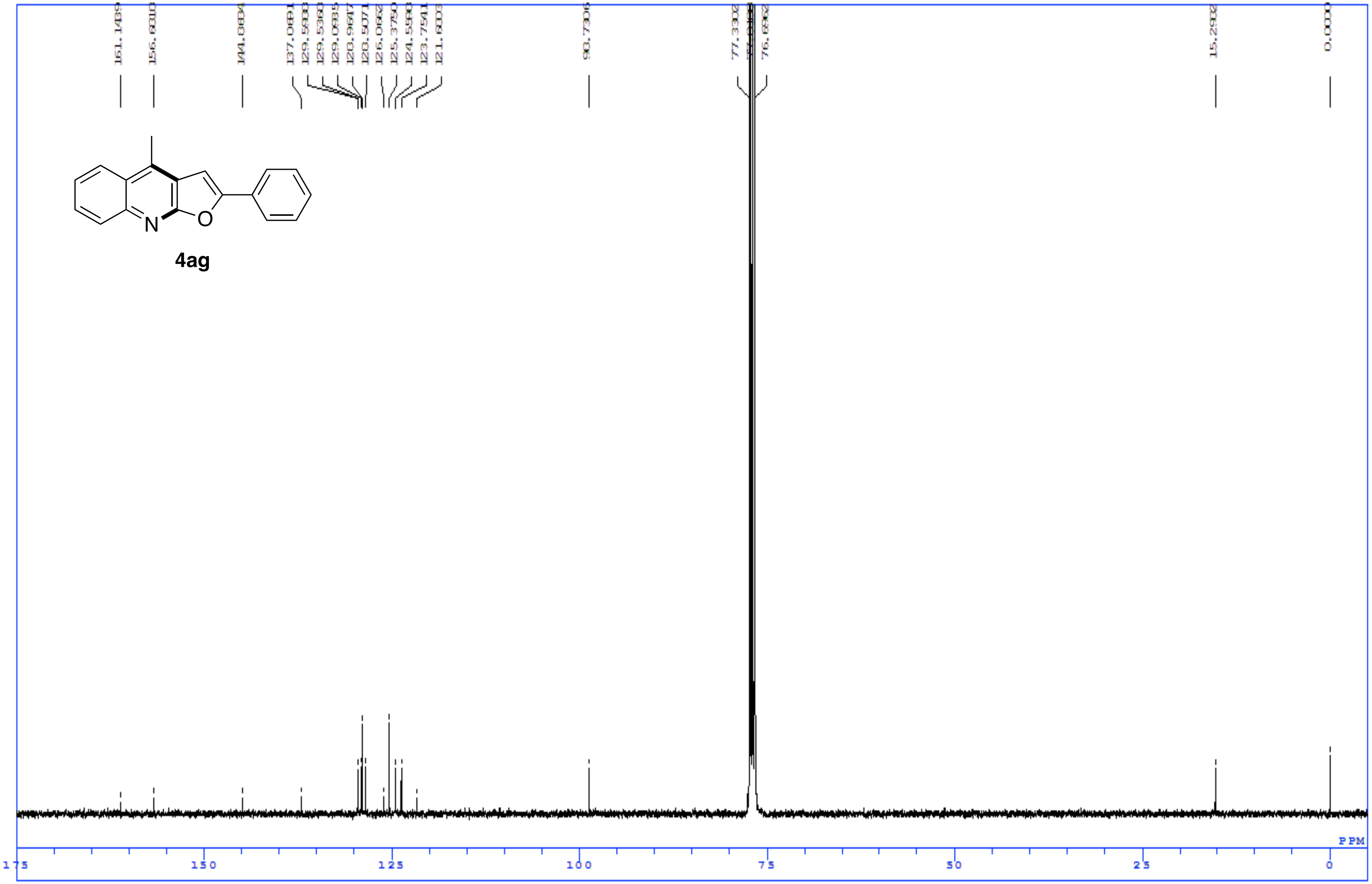

Figure S50. ^{13}C NMR spectrum of compound **4ag** in $CDCl_3$.

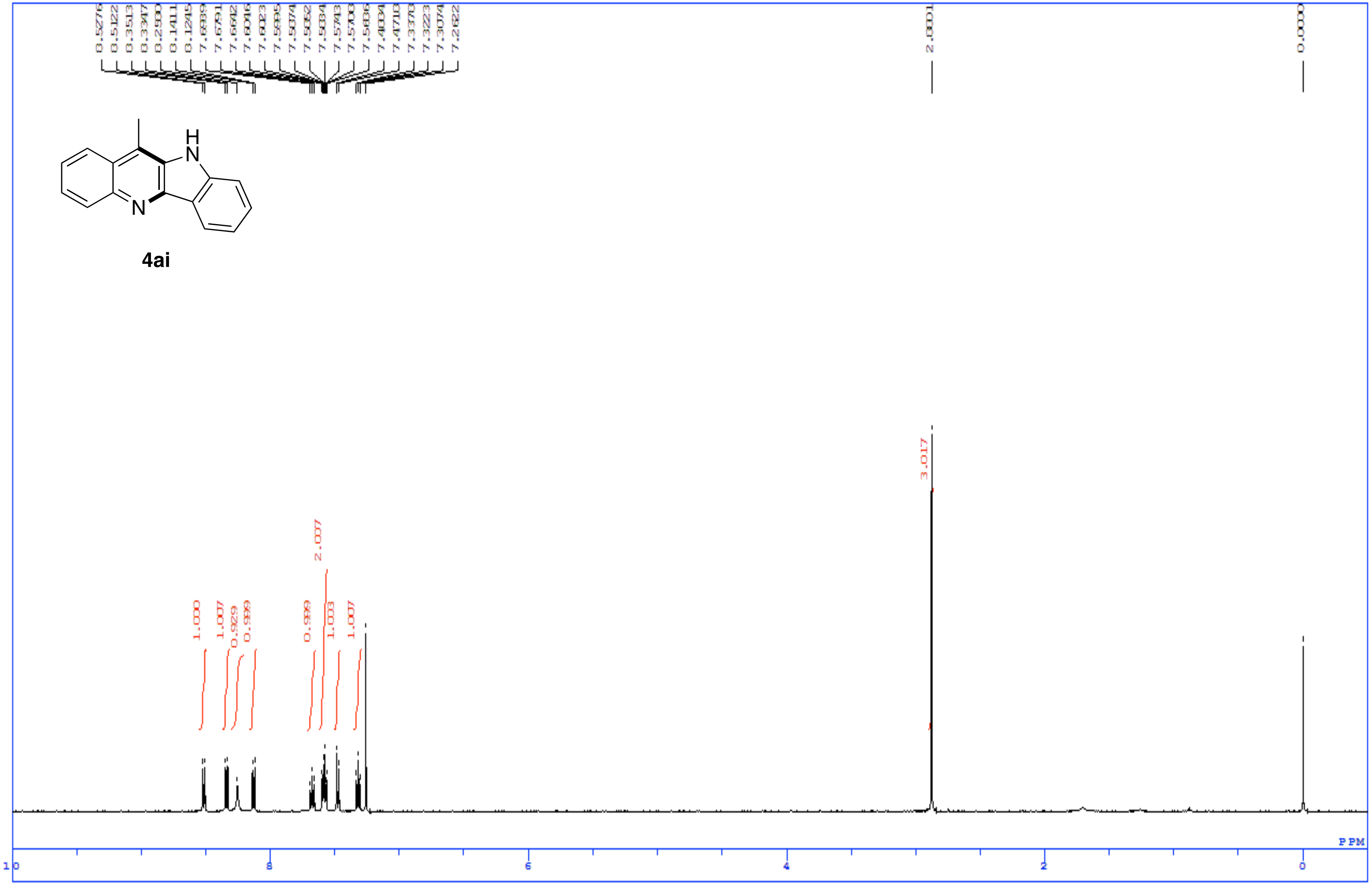

Figure S51. ^{1}H NMR spectrum of compound **4ai** in $CDCl_3$.

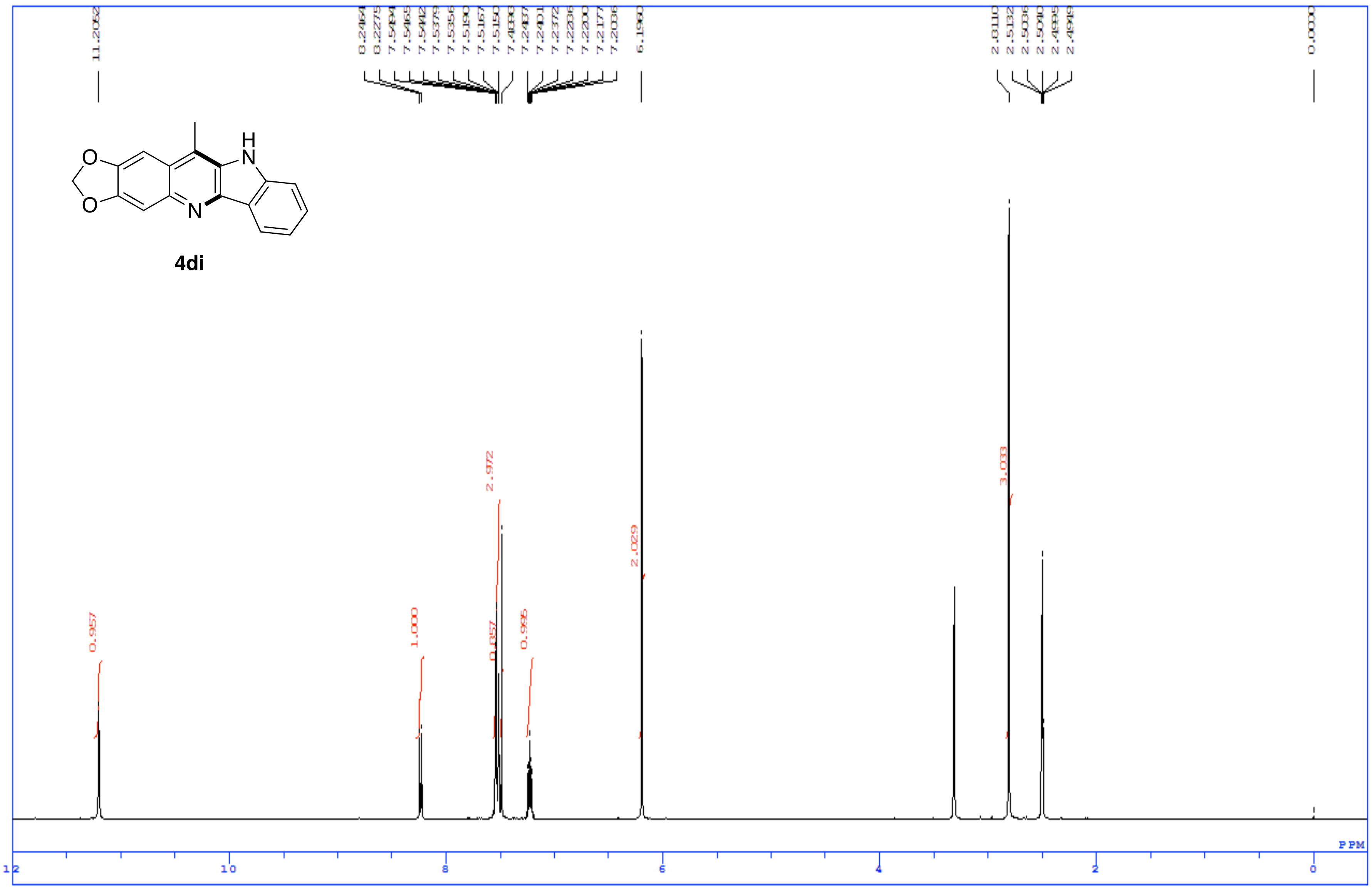

Figure S52. ^{1}H NMR spectrum of compound **4di** in dimethyl sulfoxide-d_6.

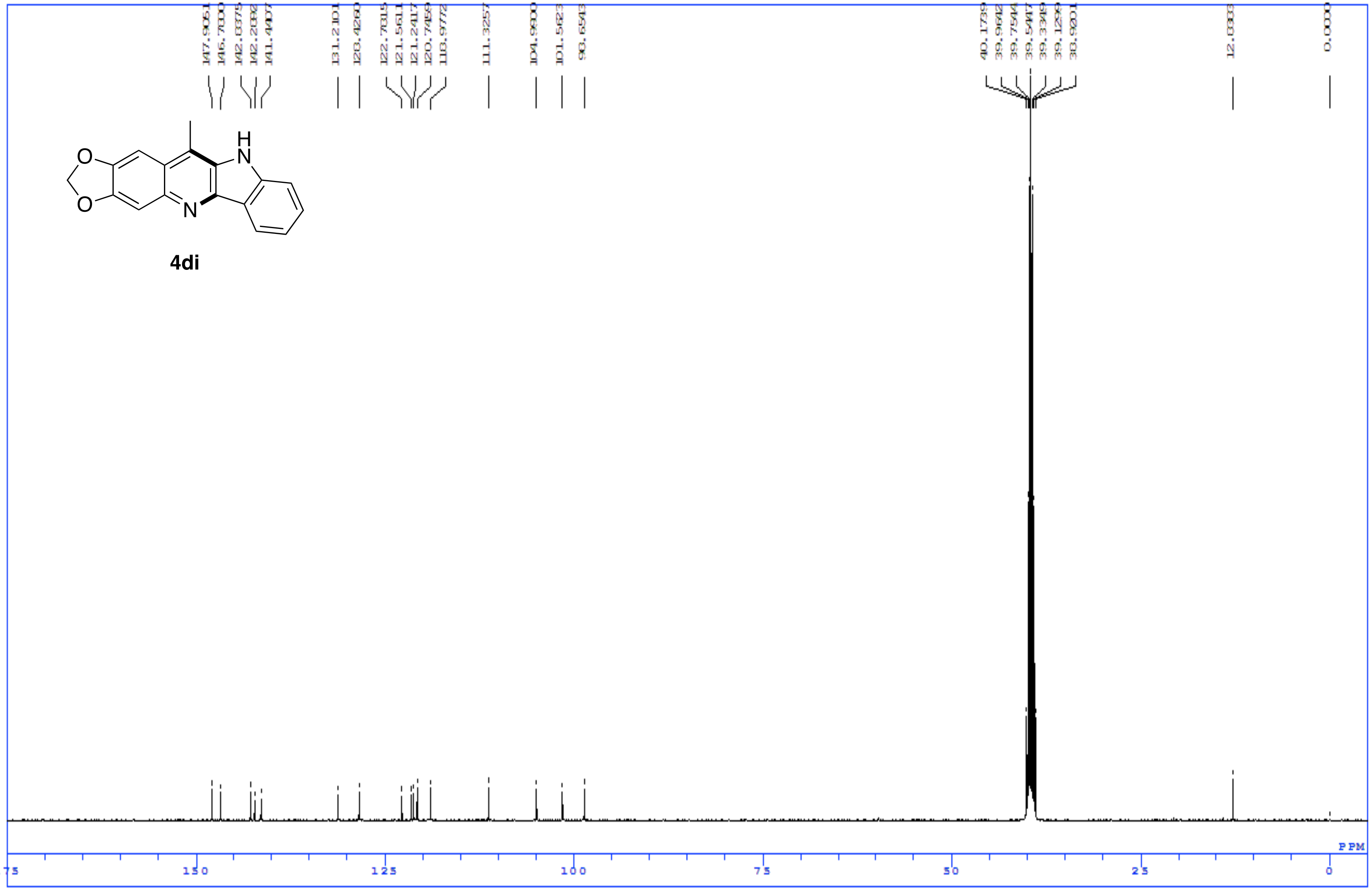

Figure S53. ^{13}C NMR spectrum of compound **4di** in dimethyl sulfoxide-d_6.

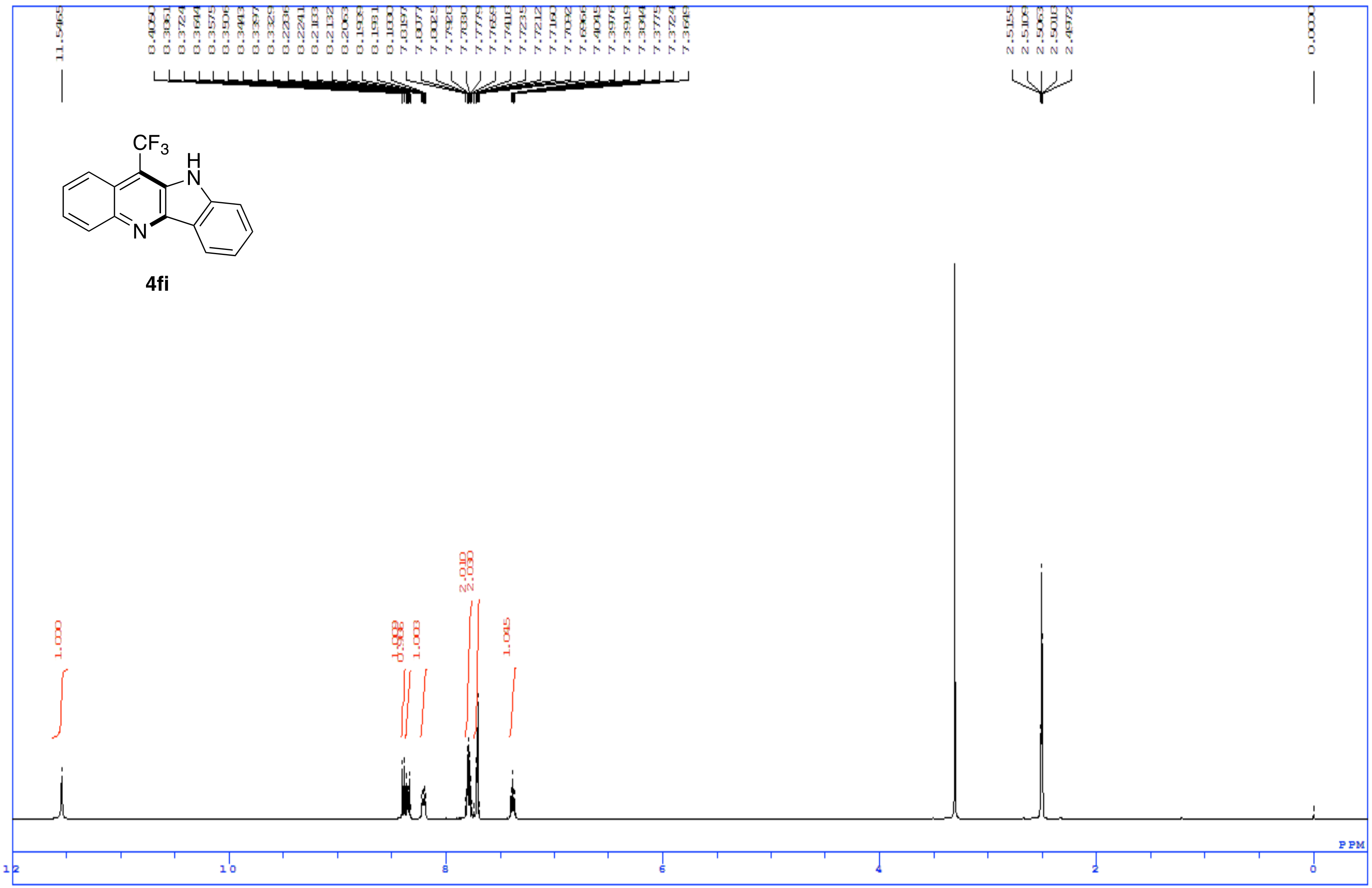

Figure S54. ^{1}H NMR spectrum of compound **4fi** in dimethyl sulfoxide-d_6.

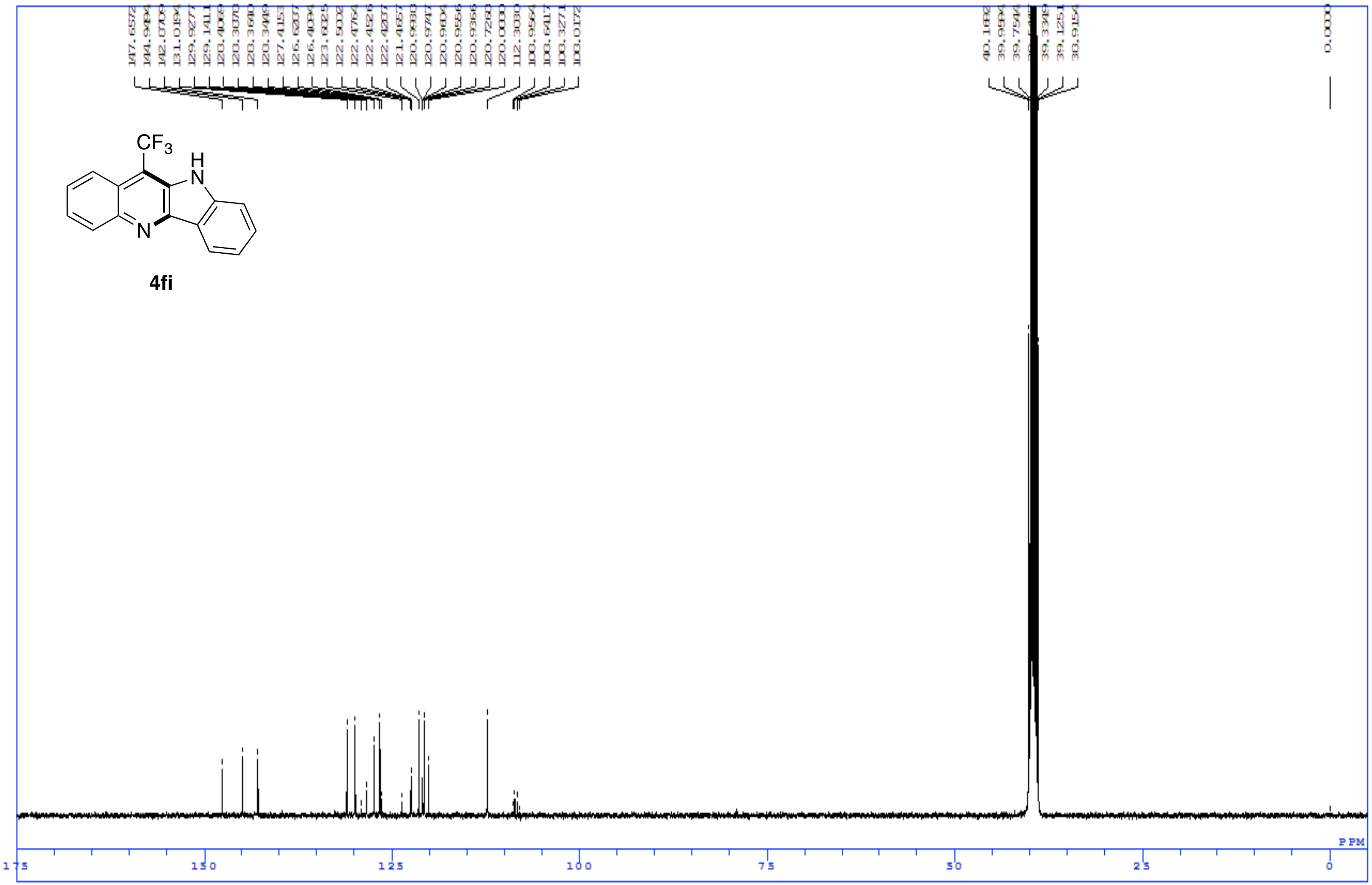

Figure S55. ^{13}C NMR spectrum of compound **4fi** in dimethyl sulfoxide-d_6.

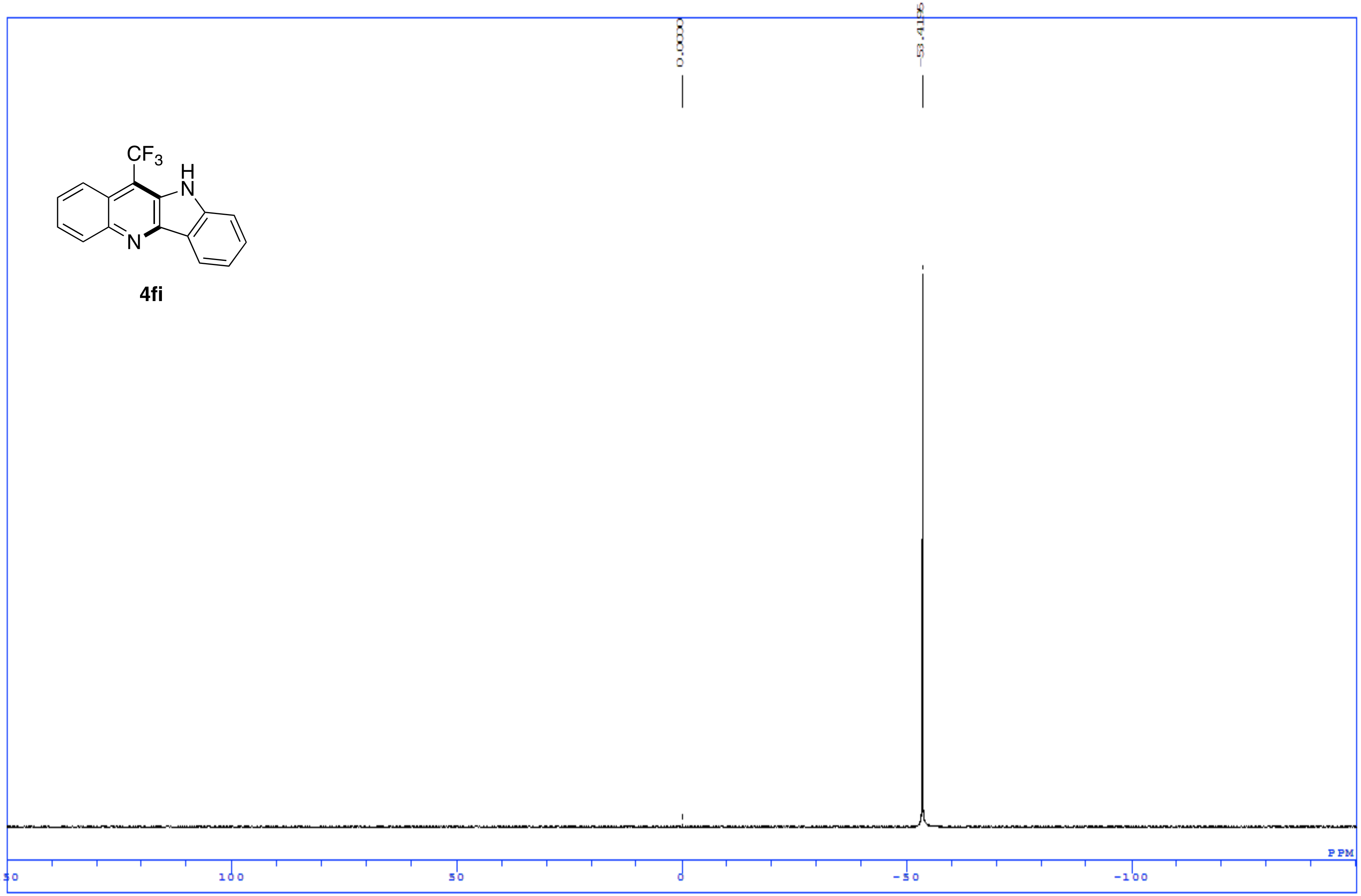

Figure S56. ^{19}F NMR spectrum of compound **4fi** in dimethyl sulfoxide-d_6.

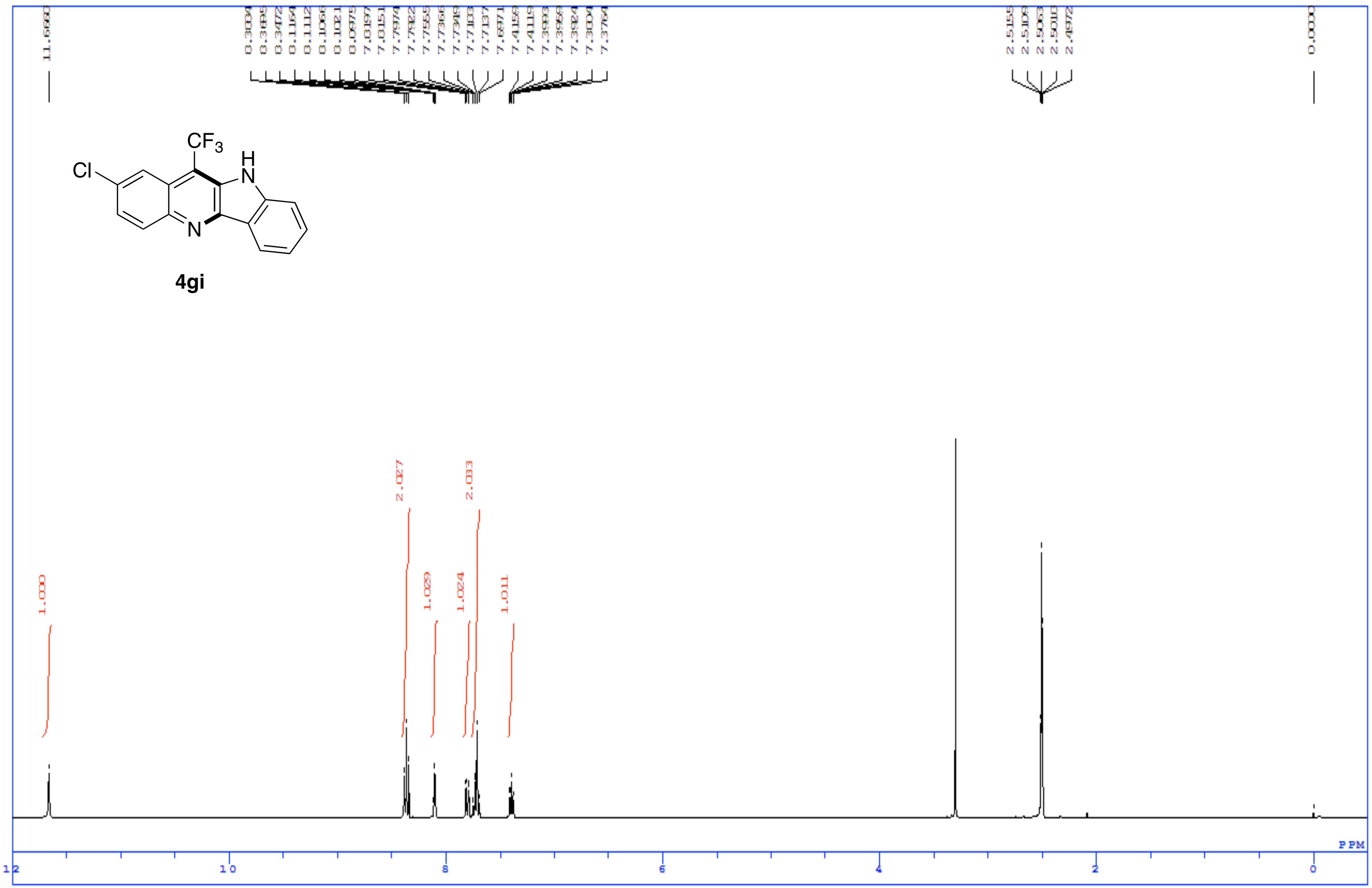

Figure S57. ^{1}H NMR spectrum of compound **4gi** in dimethyl sulfoxide-d_6.

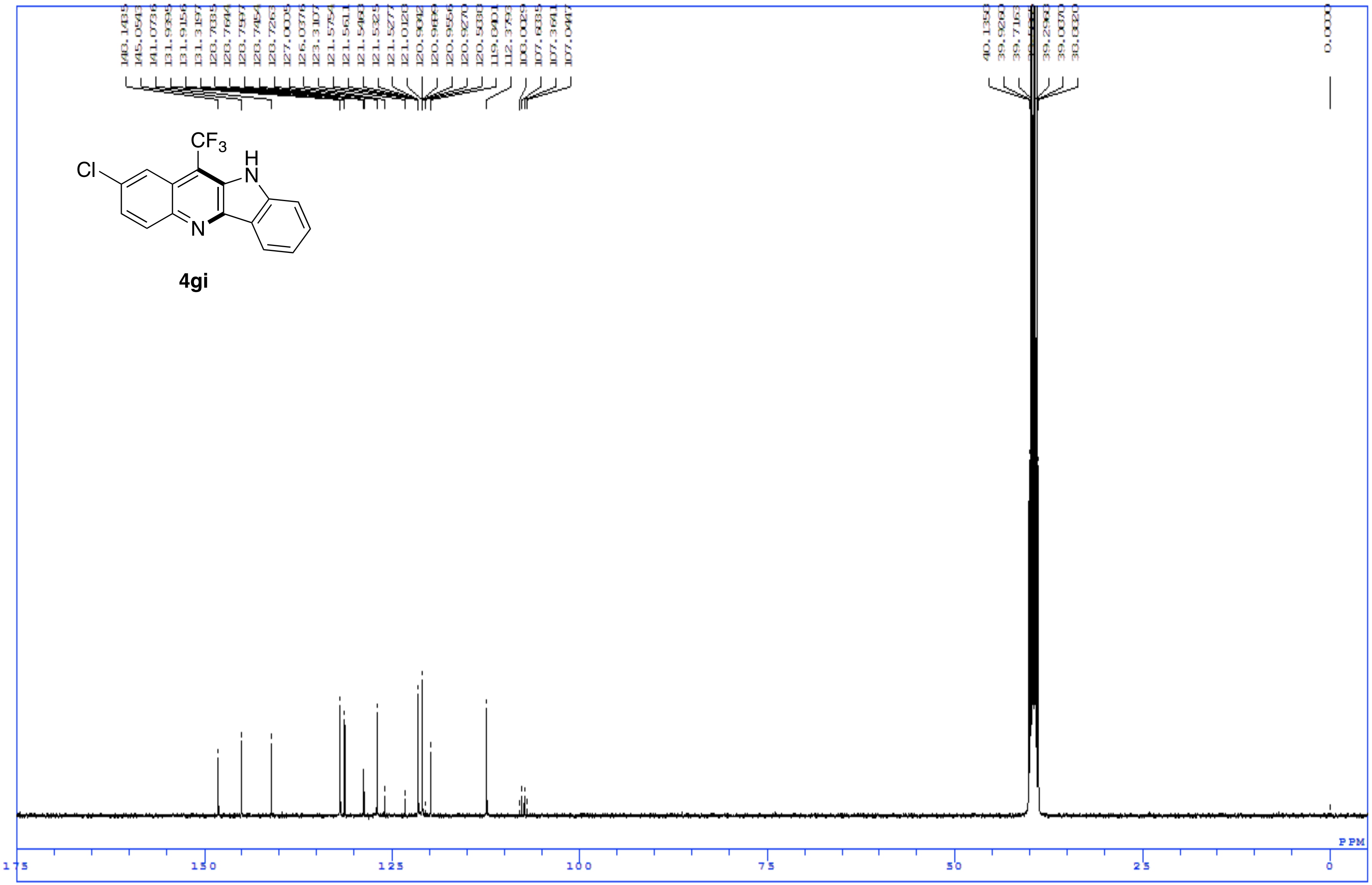

Figure S58. ^{13}C NMR spectrum of compound **4gi** in dimethyl sulfoxide-d_6.

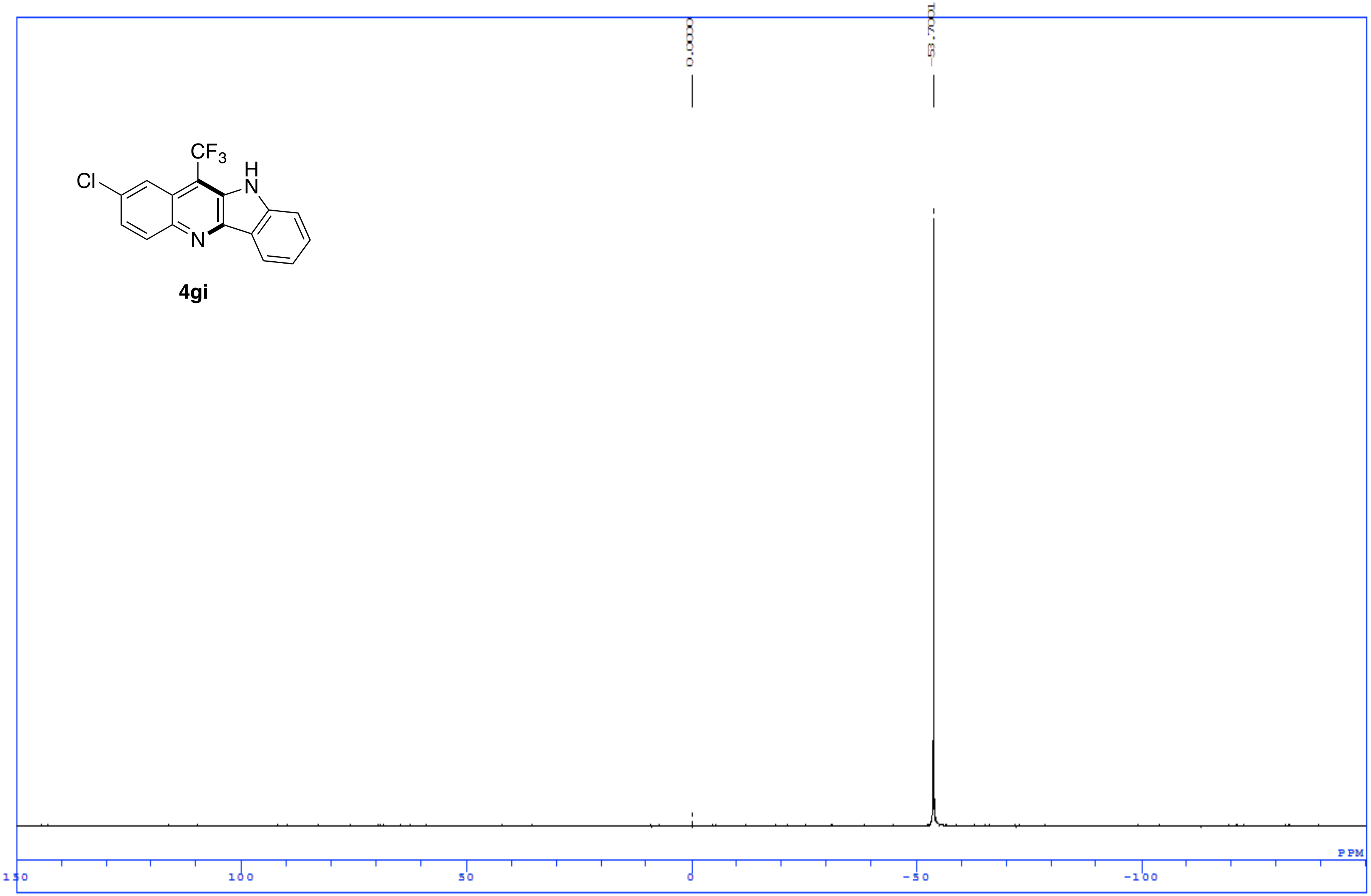

Figure S59. ^{19}F NMR spectrum of compound **4gi** in dimethyl sulfoxide-d_6.

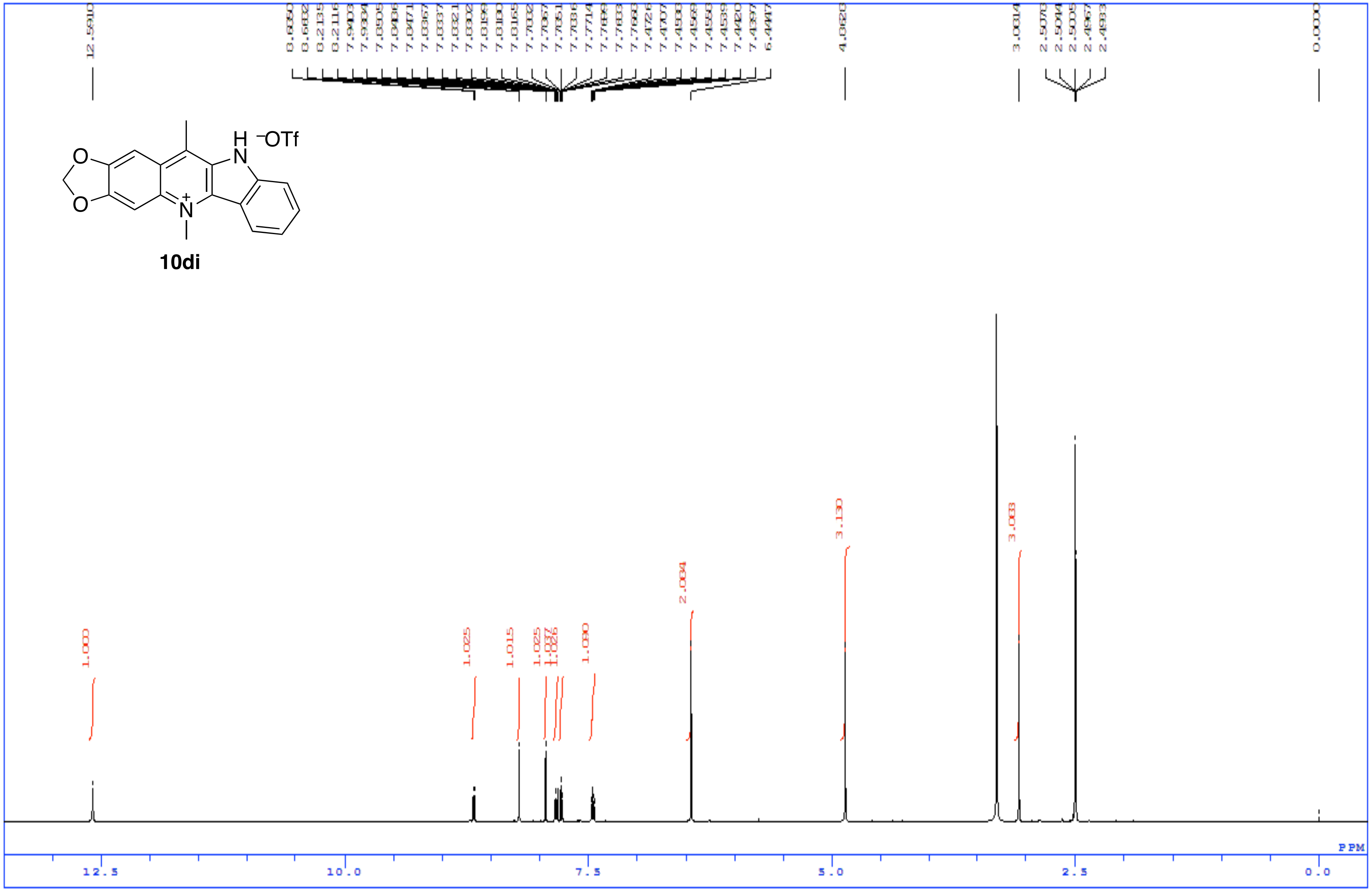

Figure S60. ^{1}H NMR spectrum of compound **10di** in dimethyl sulfoxide-d_6.

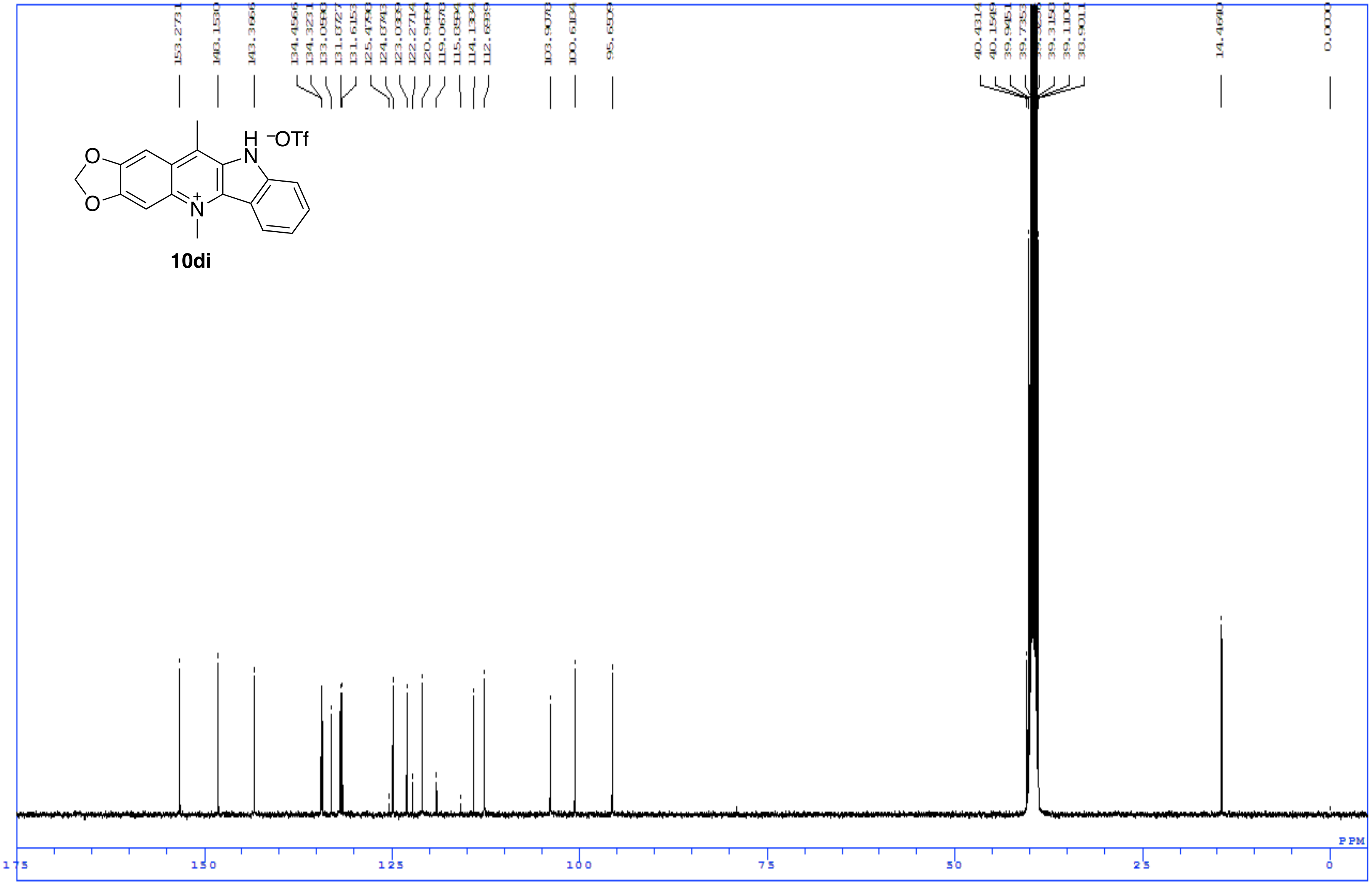

Figure S61. ^{13}C NMR spectrum of compound **10di** in dimethyl sulfoxide-d_6.

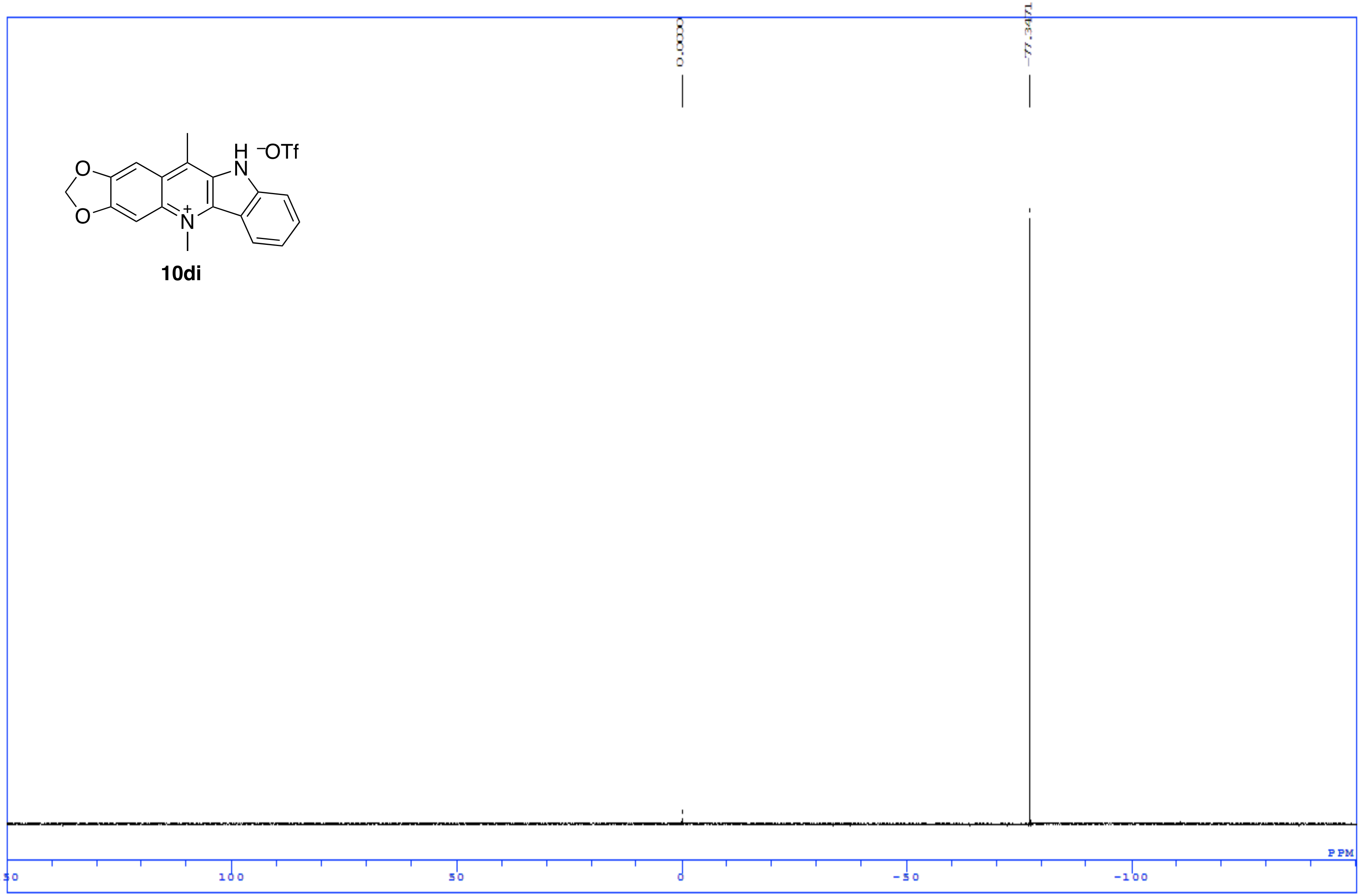

Figure S62. ^{19}F NMR spectrum of compound **10di** in dimethyl sulfoxide-d_6.

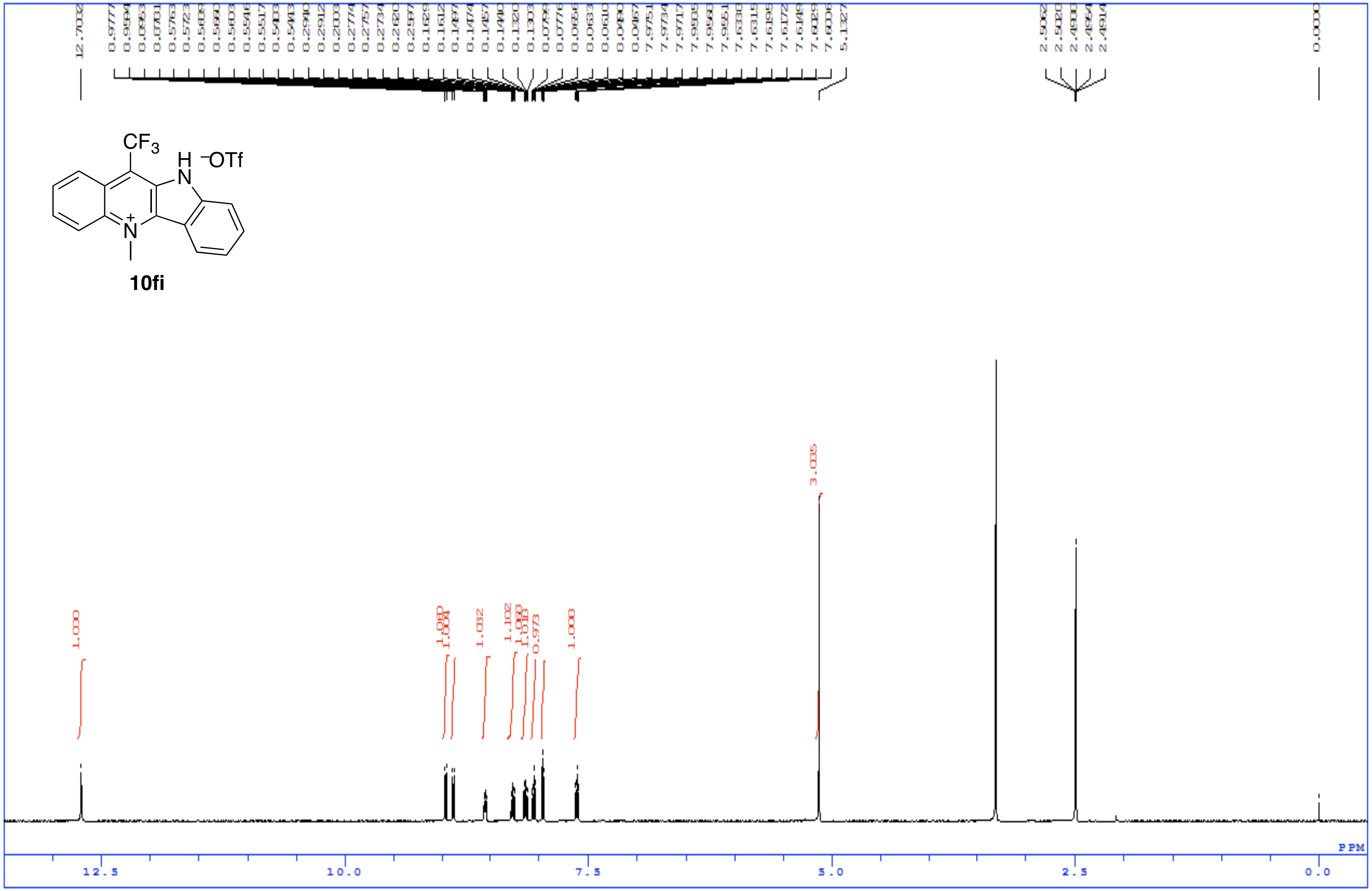

Figure S63. ^{1}H NMR spectrum of compound **10fi** in dimethyl sulfoxide-d_6.

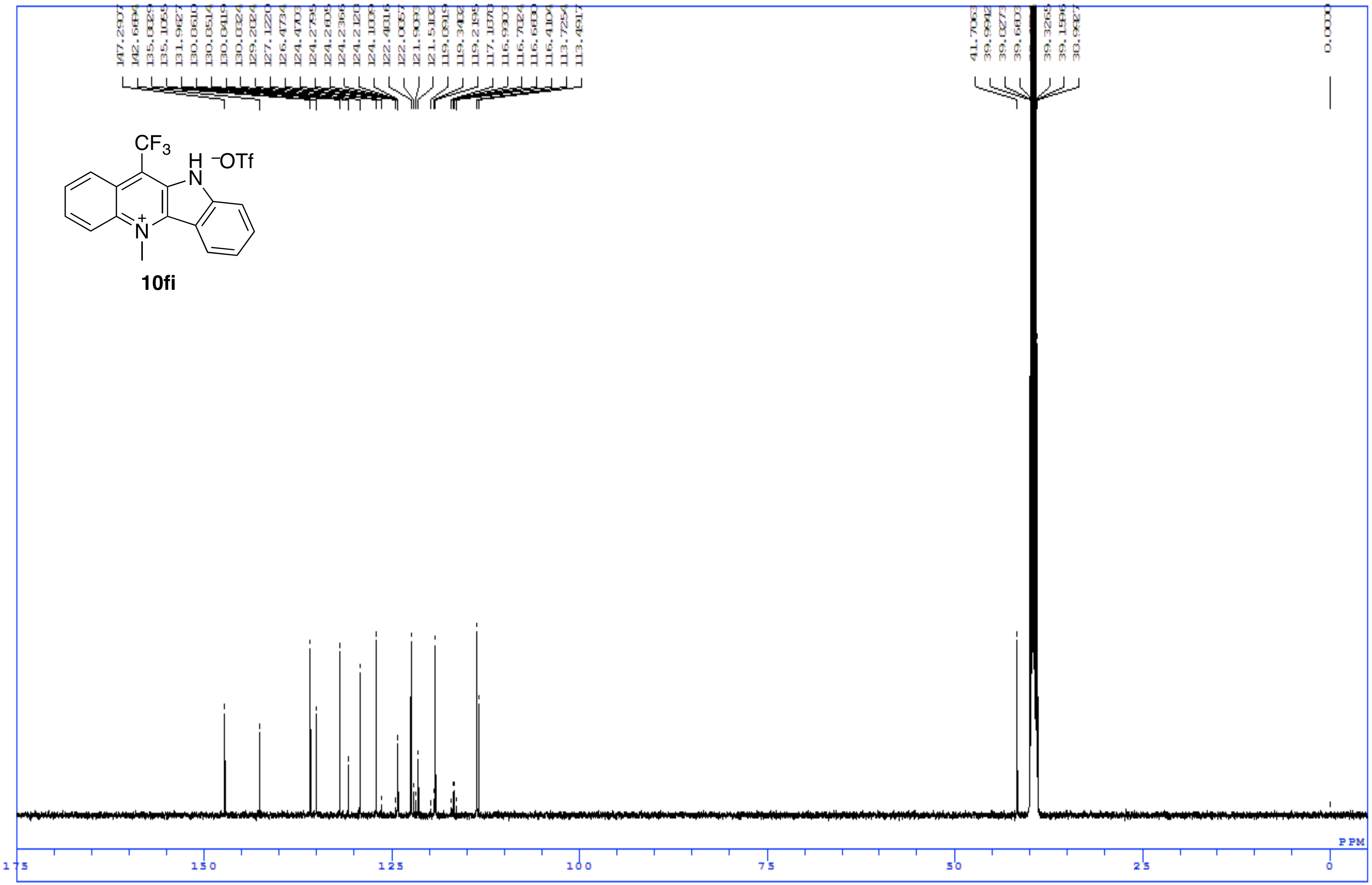

Figure S64. ^{13}C NMR spectrum of compound **10fi** in dimethyl sulfoxide-d_6.

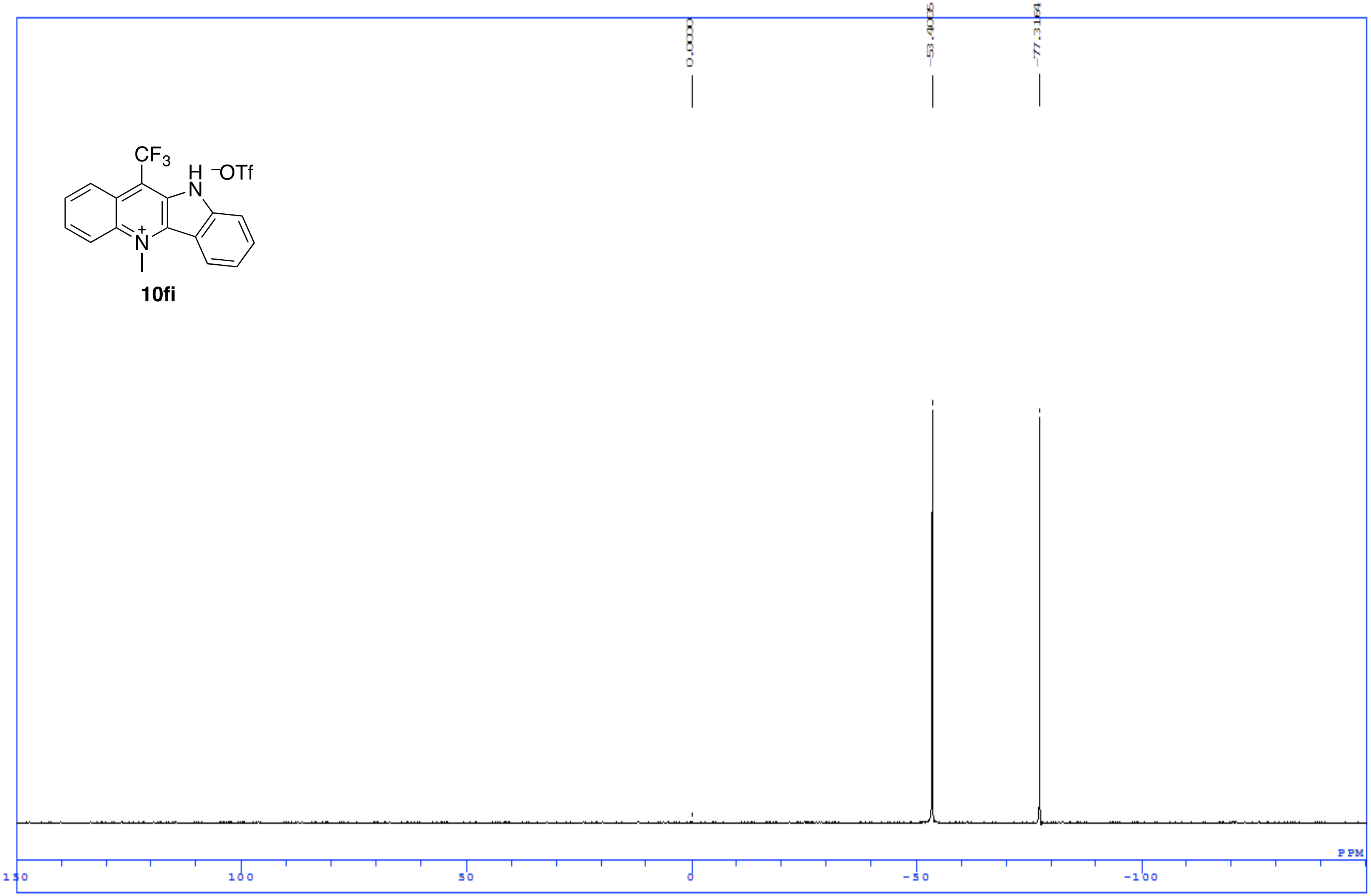

Figure S65. ^{19}F NMR spectrum of compound **10fi** in dimethyl sulfoxide-d_6.

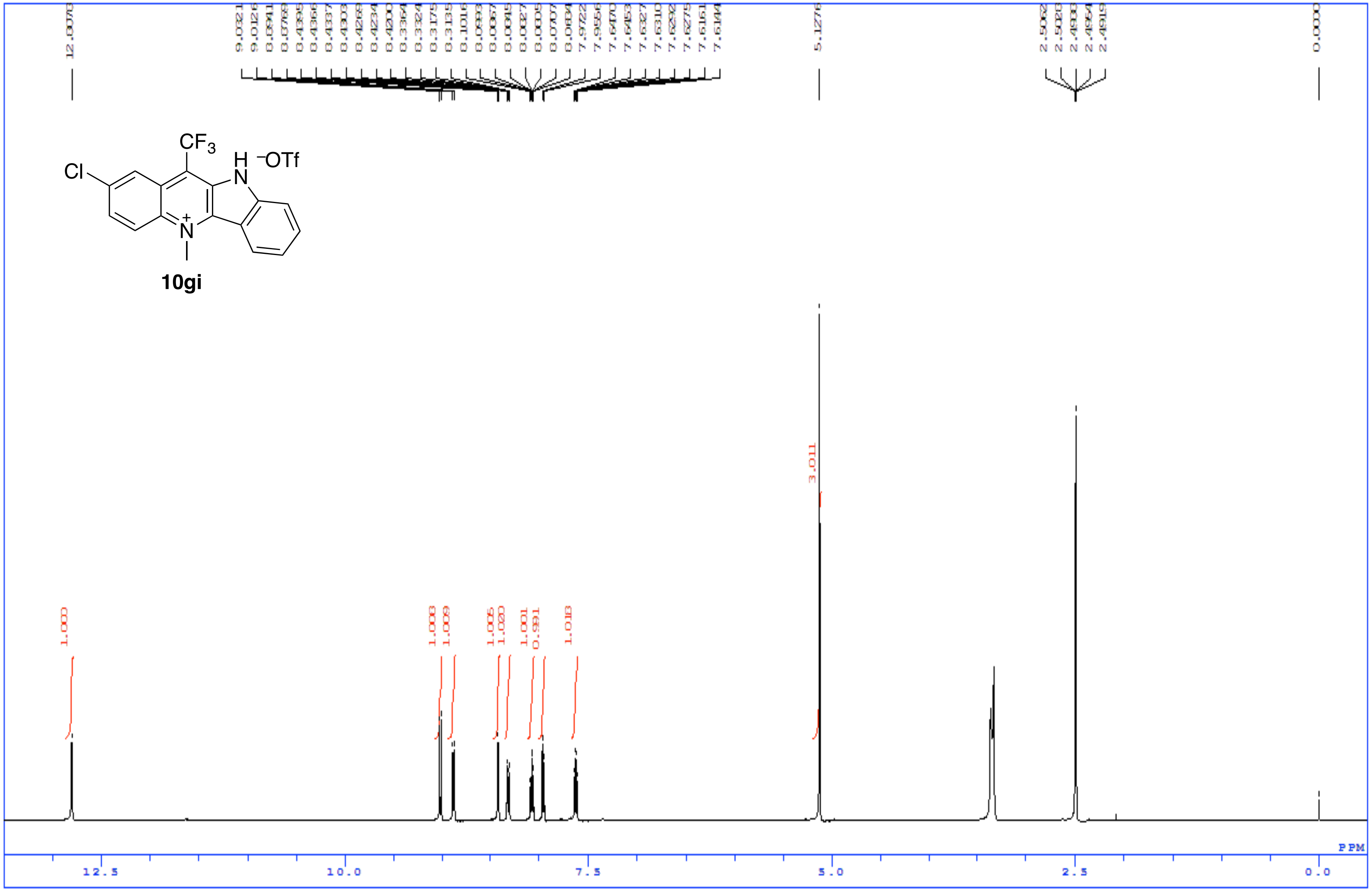

Figure S66. ^{1}H NMR spectrum of compound **10gi** in dimethyl sulfoxide-d_6.

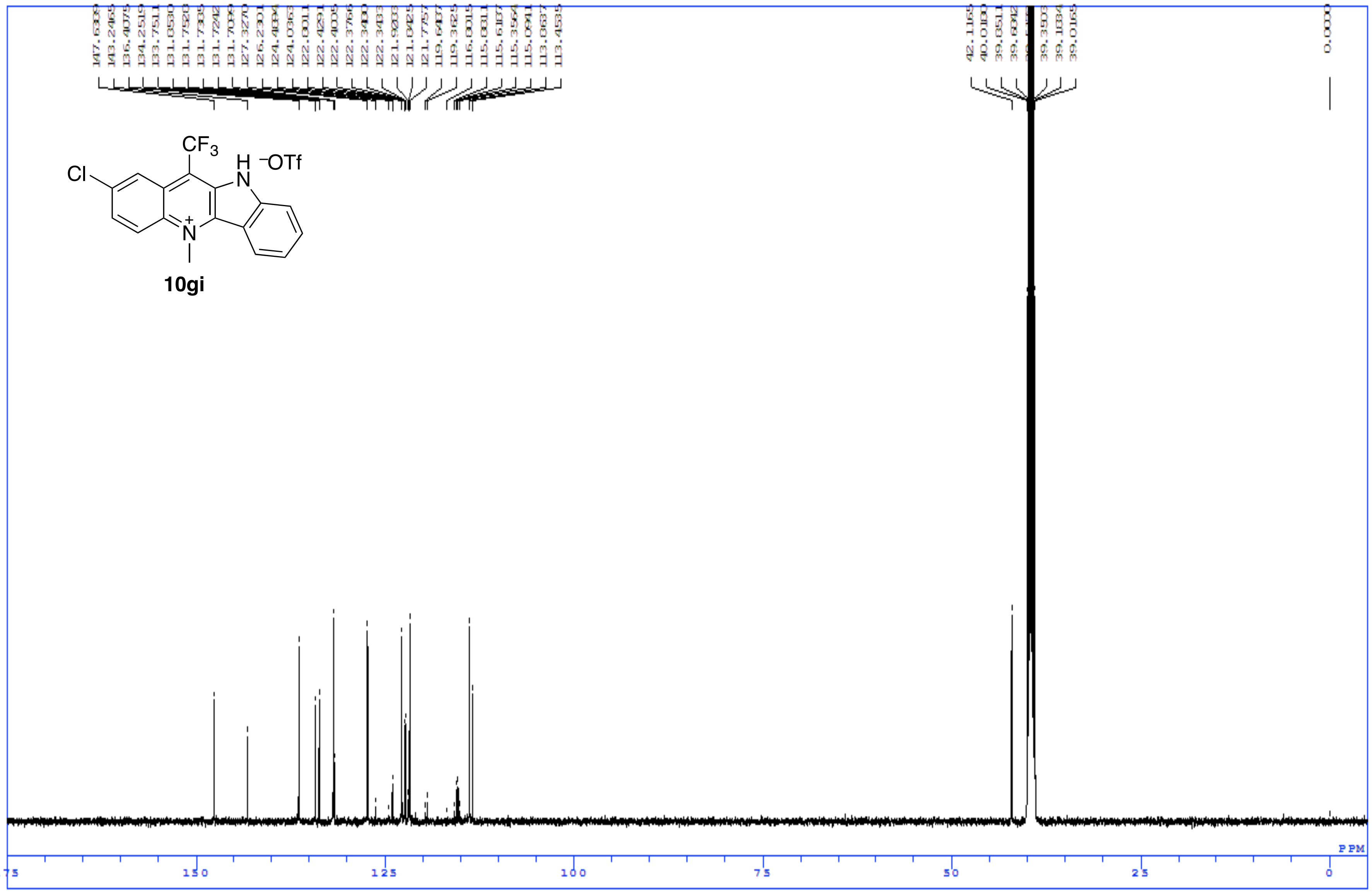

Figure S67. ^{13}C NMR spectrum of compound **10gi** in dimethyl sulfoxide-d_6.

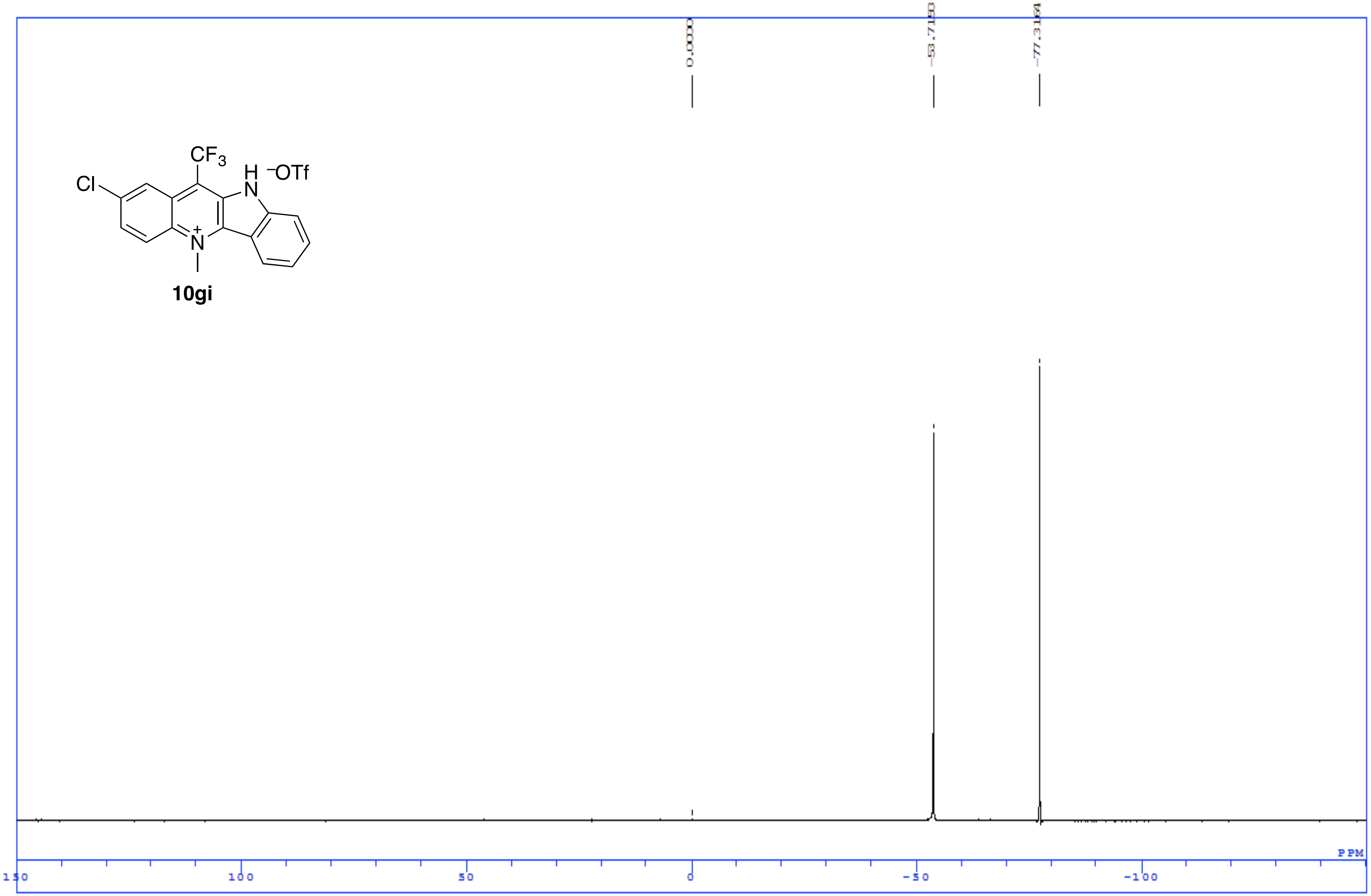

Figure S68. ^{19}F NMR spectrum of compound **10gi** in dimethyl sulfoxide-d_6.

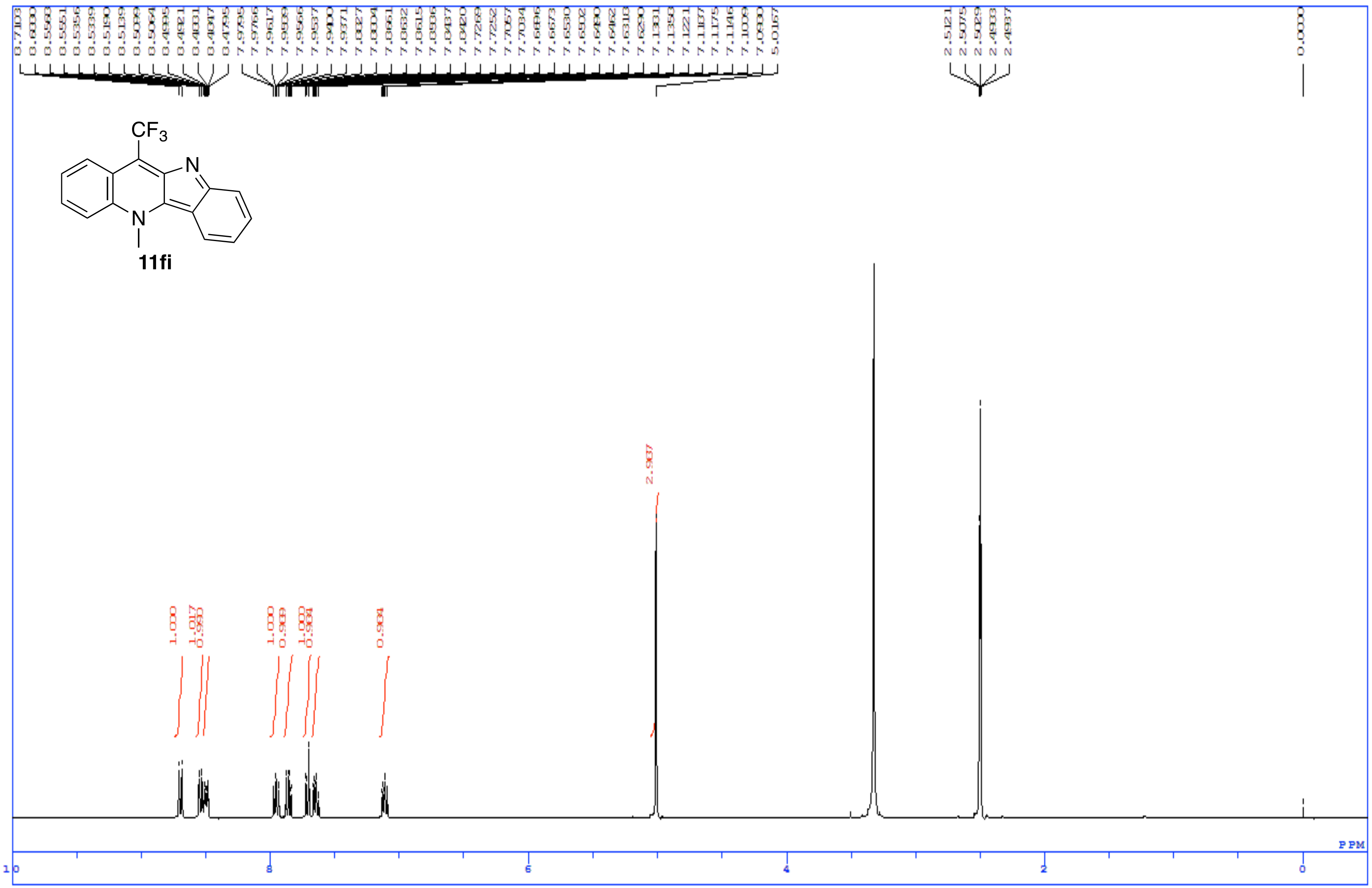

Figure S69. ^{1}H NMR spectrum of compound **11fi** in dimethyl sulfoxide-d_6.

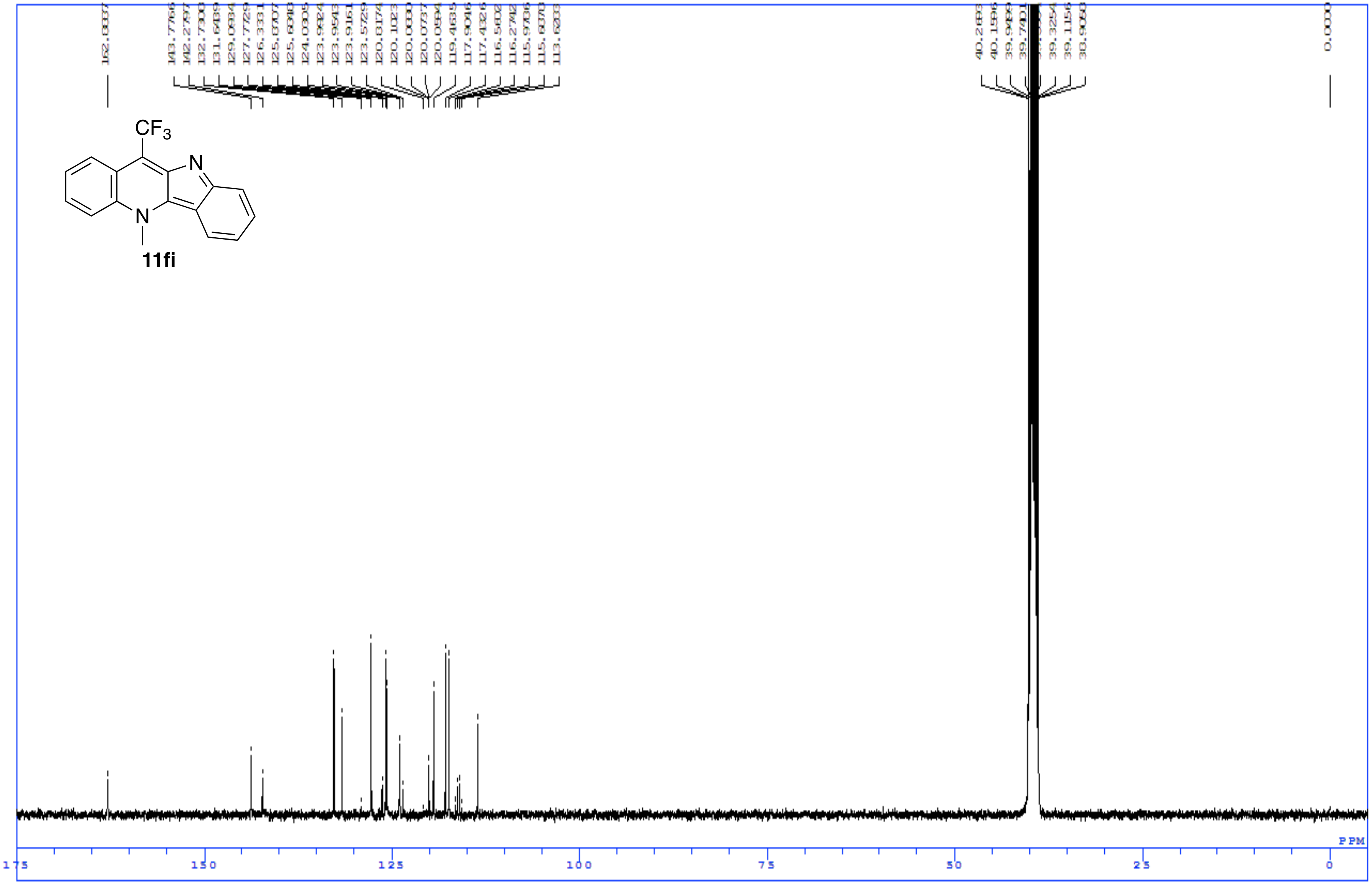

Figure S70. ^{13}C NMR spectrum of compound **11fi** in dimethyl sulfoxide-d_6.

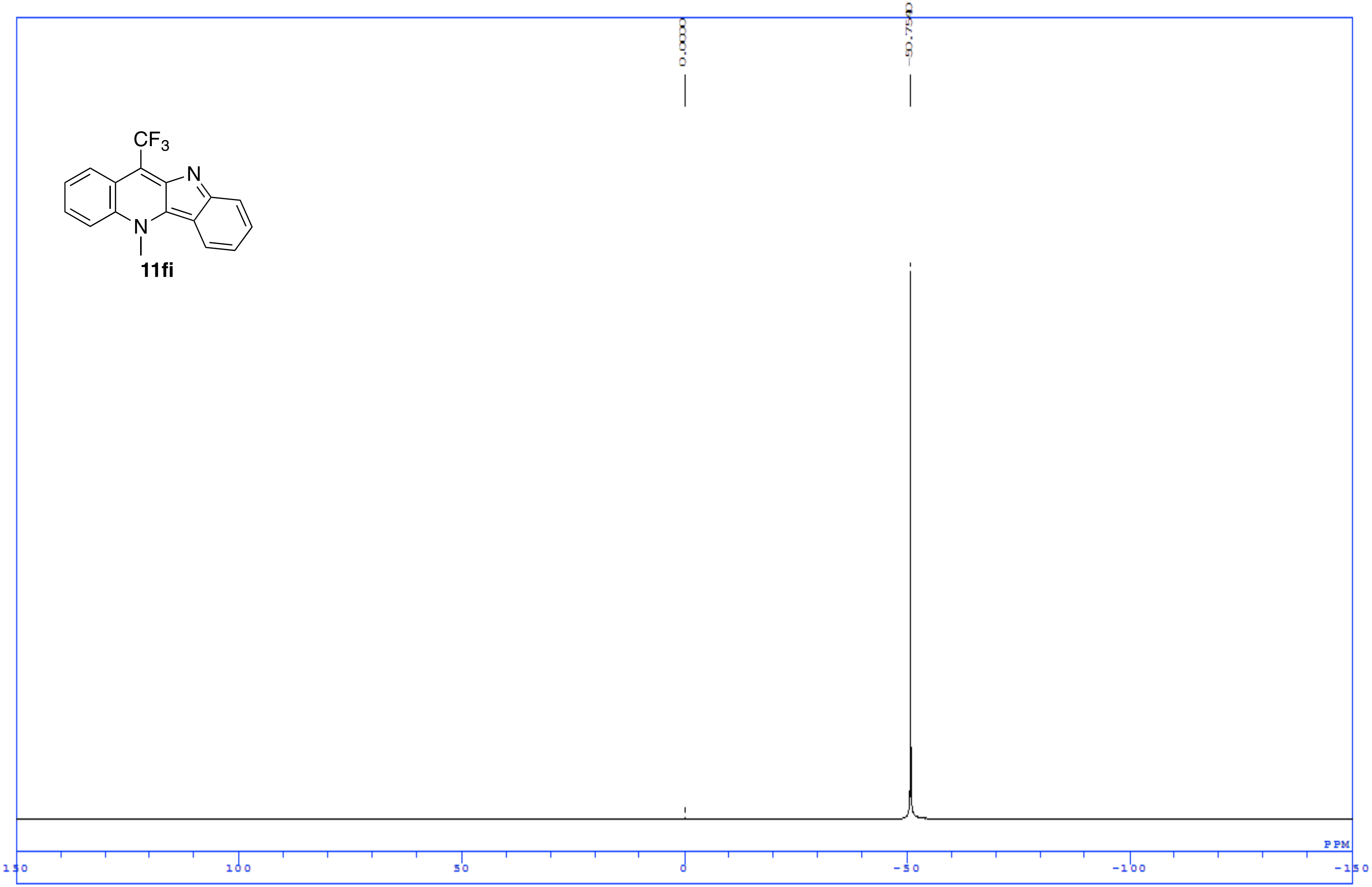

Figure S71. ^{19}F NMR spectrum of compound **11fi** in dimethyl sulfoxide-d_6.

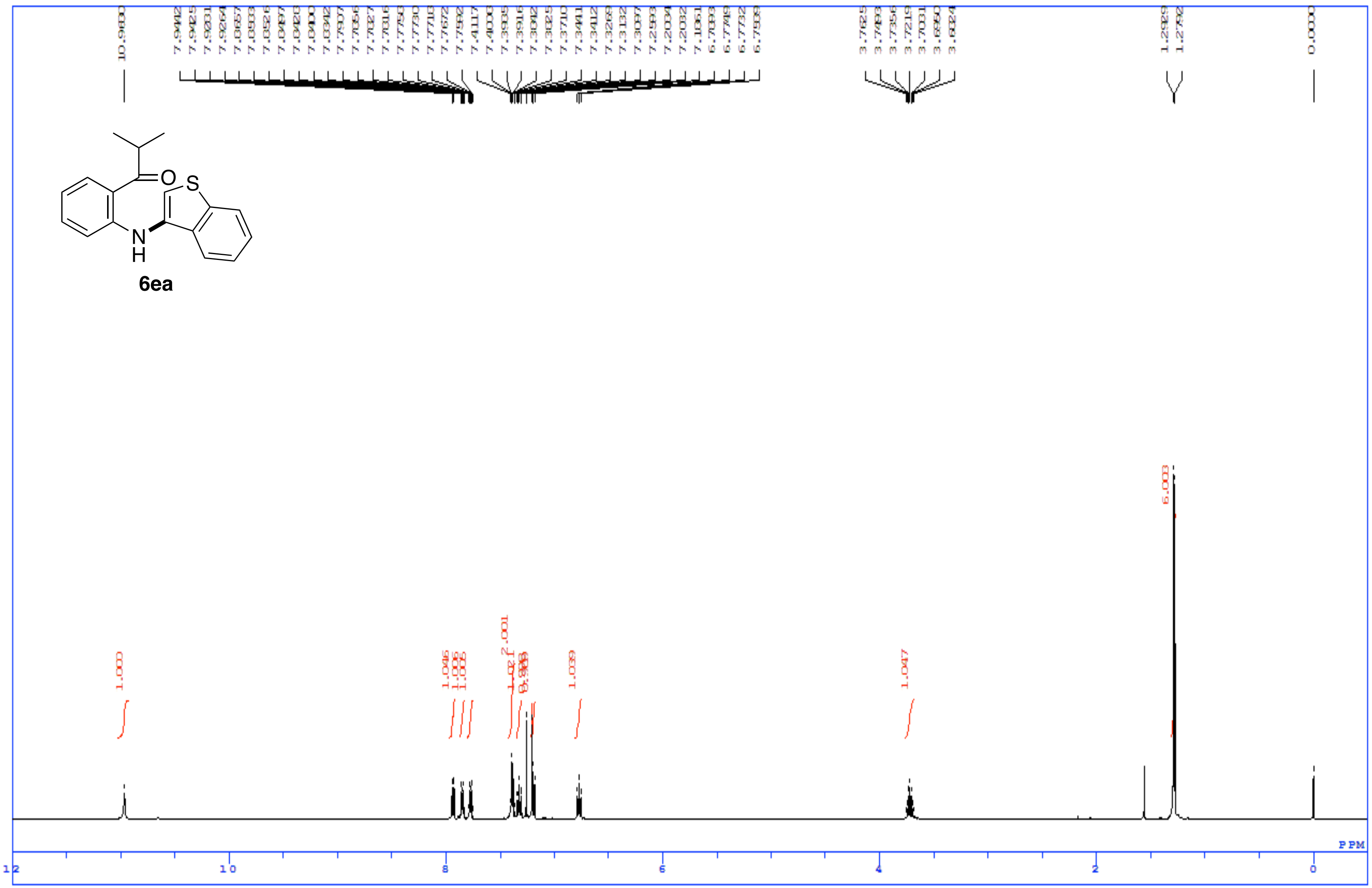

Figure S72. ^{1}H NMR spectrum of compound **6ea** in $CDCl_3$.

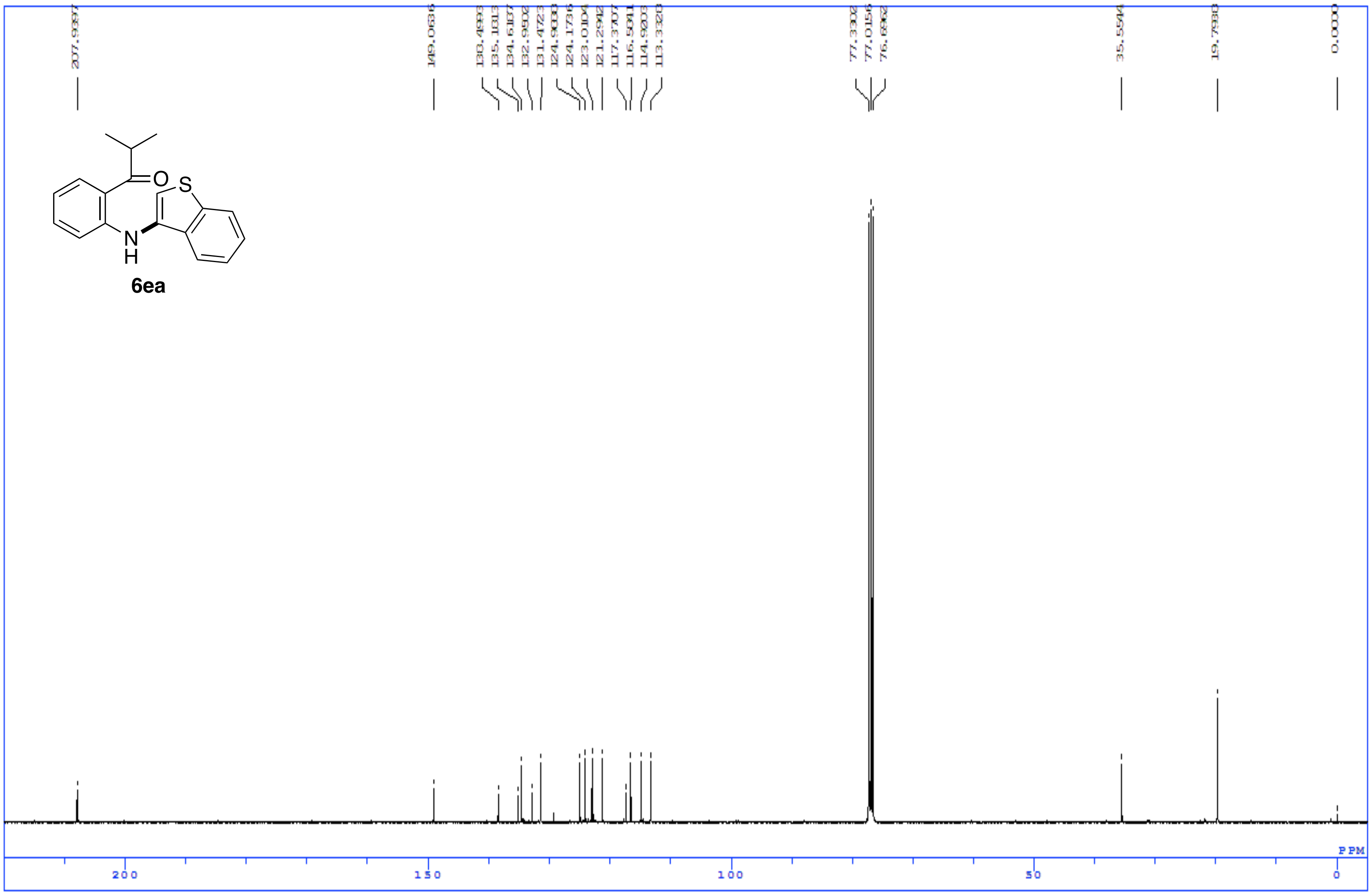

6ea

Figure S73. ^{13}C NMR spectrum of compound **6ea** in $CDCl_3$.
